# NADPH-producing enzymes restrict the formation of pancreatic precancerous lesions

**DOI:** 10.1038/s42255-026-01496-x

**Published:** 2026-04-01

**Authors:** Megan D. Radyk, Barbara S. Nelson, Mariana Tannus Ruckert, Christopher J. Halbrook, Mengrou Shan, Jonathan M. Alektiar, Brooke L. Lavoie, Lucie Salvatore, Wei Yan, Matthew D. Perricone, Kathryn Buscher, Alexander Wood, Hanna S. Hong, Peter Sajjakulnukit, Li Zhang, Gabriel Corfas, Filip Bednar, Timothy L. Frankel, Marina Pasca di Magliano, Justin A. Colacino, Yatrik M. Shah, Howard C. Crawford, Costas A. Lyssiotis

**Affiliations:** 1 Department of Molecular and Integrative Physiology, University of Michigan, Ann Arbor, MI USA; 2 Graduate Program in Cancer Biology, University of Michigan, Ann Arbor, MI USA; 3 Department of Molecular Biology and Biochemistry, University of California Irvine, Irvine, CA USA; 4 University of California Irvine Chao Family Comprehensive Cancer Center, Orange, CA USA; 5 Department of Surgery, University of Michigan, Ann Arbor, MI USA; 6 Graduate Program in Immunology, University of Michigan, Ann Arbor, MI USA; 7 Kresge Hearing Research Institute and Dept. of Otolaryngology, Head and Neck Surgery, University of Michigan, Ann Arbor, MI USA; 8 Rogel Cancer Center, University of Michigan, Ann Arbor, MI USA; 9 Rogel and Blondy Center for Pancreatic Cancer, Rogel Cancer Center, University of Michigan, Ann Arbor, MI USA; 10 Department of Cell and Developmental Biology, University of Michigan, Ann Arbor, MI USA; 11 Department of Environmental Health Sciences, School of Public Health, University of Michigan, Ann Arbor, MI USA; 12 Department of Nutritional Sciences, School of Public Health, University of Michigan, Ann Arbor, MI USA; 13 Program in the Environment, College of Literature, Sciences, and the Arts, University of Michigan, Ann Arbor, MI USA; 14 Department of Internal Medicine, Division of Gastroenterology and Hepatology, University of Michigan, Ann Arbor, MI USA; 15 Department of Surgery, Henry Ford Pancreatic Cancer Center, Henry Ford Health System, Detroit, MI USA; 16 Department of Pharmacology and Toxicology, Michigan State University, East Lansing, MI USA; 17 Present Address: Department of Pharmacology and Therapeutics, Roswell Park Comprehensive Cancer Center, Buffalo, NY USA

**Keywords:** Cancer metabolism, Pancreatic cancer, Mechanisms of disease, Metabolism

## Abstract

Acinar-to-ductal metaplasia (ADM) is a reversible cell state that facilitates pancreas repair following injury. Oncogenic *KRAS* mutations can progress ADM to pancreatic intraepithelial neoplasia (PanIN) and pancreatic ductal adenocarcinoma (PDAC). However, the metabolic alterations in these precancerous lesions are understudied. Here, we identify global changes in central carbon metabolism genes and metabolites during ADM formation. In particular, NRF2-target genes are significantly induced in ADM. Among these, we focus on genes encoding NADPH-producing enzymes glucose-6-phosphate dehydrogenase (G6PD) and malic enzyme 1 (ME1), which participate in the regulation of oxidative stress. In mouse models of pancreatic tumourigenesis, G6PD deficiency or *Me1* loss increases reactive oxygen species and lipid peroxidation, which is accompanied by accelerated formation of ADM and PanIN lesions. Notably, *Me1* loss, but not G6PD deficiency, promotes faster PDAC progression. We demonstrate that oxidative stress is required for ADM, as pharmacological antioxidant treatment attenuates ADM progression in vivo and ex vivo. Conversely, depleting the antioxidant glutathione promotes precancerous lesions in primary human acinar cells and in mice. Together, our findings shed light on metabolic reprogramming in the precancerous pancreas.

## Abstract (web summary)

Temporal profiling of central carbon metabolism during *Kras*^G12D^-driven acinar-to-ductal metaplasia reveals that disruptions in NADPH-producing enzymes accelerate the formation of pancreatic precancerous lesions.

## Main

Oncogenic *KRAS* mutations are initiating events in PDAC^1^. PDAC has a low 5-year survival rate owing to few early detection methods, a paucity of effective therapies and late-stage diagnosis^2^. PDAC tumourigenesis progresses stepwise, beginning with ADM, followed by PanIN lesions. ADM is a reversible, healing response after pancreatic injury or inflammation, in which acinar cells transdifferentiate into ductal progenitor-like cells, proliferate and restore damaged tissue^3–5^. Upon healing, ADM cells redifferentiate into acinar cells; however, oncogenic *KRAS* blocks this process, leading to persistent ADM and PanIN formation. PanIN lesions are common in otherwise healthy tissue^6,7^, underscoring the need to define mechanisms that control precancer formation and progression^8^.

Pancreatic cancer cells require biosynthetic precursors and energy to sustain growth^9–11^. Although metabolic reprogramming in PDAC is well characterized, the role of metabolism in driving precancerous lesions remains underexplored. Requirements for acetyl-CoA, cholesterol metabolism and tight regulation of reactive oxygen species (ROS) have been demonstrated in precancer formation and progression^12–14^. MYC and NFE2L2 (NRF2) are key transcription factors facilitating metabolic changes during cancer initiation^15–18^.

NADPH supports biosynthesis (fatty acids, cholesterol, deoxyribonucleotides) and antioxidant systems that control ROS^19^. The primary source of NADPH is G6PD, the first and rate-limiting enzyme in the oxidative pentose phosphate pathway (PPP). G6PD deficiency is an X-linked disorder and the most common human enzyme deficiency^20^, often causing haemolytic anaemia^21^. We previously showed that oncogenic *KRAS* activates the nonoxidative branch of the PPP by enhanced glycolysis and transcriptional upregulation of *Rpia* and *Rpe*^22–24^. As such, pancreatic cancer cells rely on NADPH produced by ME1 (refs. ^23,25^). Loss of TIGAR, which supports oxidative PPP activity, delays PanIN formation but enhances metastasis^14^, highlighting complex regulation of PPP flux, NADPH production and ROS control during cancer initiation and progression.

Here, we use *Kras*^G12D^-driven mouse models to define metabolic pathways supporting precancer development. G6PD deficiency accelerates ADM and PanIN formation, while pancreas-specific *Me1* loss accelerates early lesions and promotes PDAC progression. Loss of these NADPH-generating enzymes increases ROS and lipid peroxidation; antioxidant supplementation rescues accelerated lesion formation in mice and primary cultures. Inhibition of glutathione biosynthesis similarly promotes ADM in healthy human acinar cells and mice. Integrated RNA sequencing (RNA-seq) and metabolomics analyses further define metabolic changes during cancer initiation and establish the energetic demands of pancreatic precancerous lesions.

### Metabolic changes accompany ADM

**Fig. 1:**
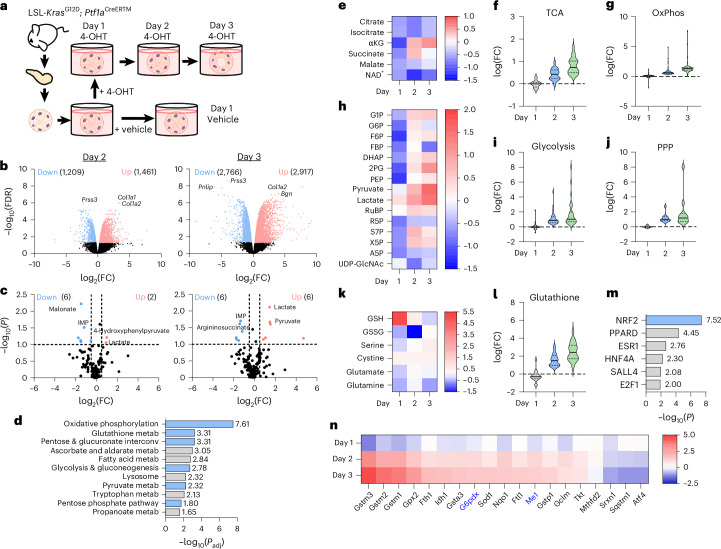
Metabolic pathways are altered during ADM. **a**, Scheme of the experimental model. Pancreata were dissected from LSL-*Kras*^G12D^; *Ptf1a*^Cre^^ERTM^ mice (*n* = 3) and acinar clusters were isolated for primary, ex vivo culture. Acinar clusters were grown free-floating in media and were treated with vehicle or 2 μM 4-OHT to induce mutant *Kras*. Cells were collected after 1, 2 or 3 days in culture. For each timepoint, cells from one well were divided for RNA extraction and metabolite analysis. **b**, Volcano plots of differentially expressed genes at day 2 and day 3 following 4-OHT treatment compared to day 1 vehicle. Significantly upregulated genes are red; significantly downregulated genes are blue. False discovery rate (FDR) < 0.05 was considered significant, log_2_(fold change (FC)) > 0.5 was considered upregulated andlog_2_(FC) < –0.5 was considered downregulated. Differential gene expression was estimated using the quasi-likelihood negative binomial generalized log-linear approach in edgeR. **c**, Volcano plots of differentially abundant metabolites when comparing day 2 and day 3 following 4-OHT treatment compared to day 1 vehicle. Significantly increased intracellular metabolites are red; decreased metabolites are blue. *P* < 0.1 was considered significant, log_2_(FC) > 0.5 was considered increased and log_2_(FC) < –0.5 was considered decreased. *P* values were calculated using a Student’s *t*-test (unpaired, two-tailed). **d**, Significant metabolism-related pathways at day 2, identified by gene set enrichment analysis (GSEA) from KEGG (Kyoto Encyclopedia of Genes and Genomes) pathways. Blue bars highlight pathways of interest. **e**, Heatmap of detected metabolites in the TCA cycle. Scale represents log_2_(FC) of median normalized abundance relative to vehicle. **f**, Violin plot of the leading-edge genes in the TCA cycle KEGG pathway. The *y* axis represents log(FC) relative to vehicle. Lines inside the plots represent the quartiles and median. **g**, Violin plot of the leading-edge genes in the oxidative phosphorylation (OxPhos) KEGG pathway. The *y* axis represents log(FC) relative to vehicle. Lines inside the plots represent the quartiles and median. **h**, Heatmap of metabolites related to glycolysis and the PPP. Scale represents log_2_(FC) of median normalized abundance relative to vehicle. **i**, Violin plot of the leading-edge genes in the glycolysis and gluconeogenesis KEGG pathway. The *y* axis represents log(FC) relative to vehicle. Lines inside the plots represent the quartiles and median. **j**, Violin plot the leading-edge genes in the PPP KEGG pathways. The *y* axis represents log(FC) relative to vehicle. Lines inside the plots represent the quartiles and median. **k**, Heatmap of metabolites related to glutathione metabolism. Scale represents log_2_(FC) of median normalized abundance relative to vehicle. GSSG, oxidized glutathione. **l**, Violin plot of leading-edge genes in the glutathione metabolism KEGG pathway. The *y* axis represents log(FC) relative to vehicle. Lines inside the plots represent the quartiles and median. **m**, Transcription factor enrichment analysis generated using Enrichr from ENCODE and ChEA consensus transcription factors. The top 500 upregulated genes from day 2 were analysed. The blue bar highlights a transcription factor of interest. Enrichr was used to identify differentially enriched pathways and calculate *P* values with Fisher’s exact test. **n**, Heatmap showing log(FC) of significantly differentially expressed NRF2-target genes. Genes under investigation are indicated with blue text. Source data

**Extended Data Fig. 1:**
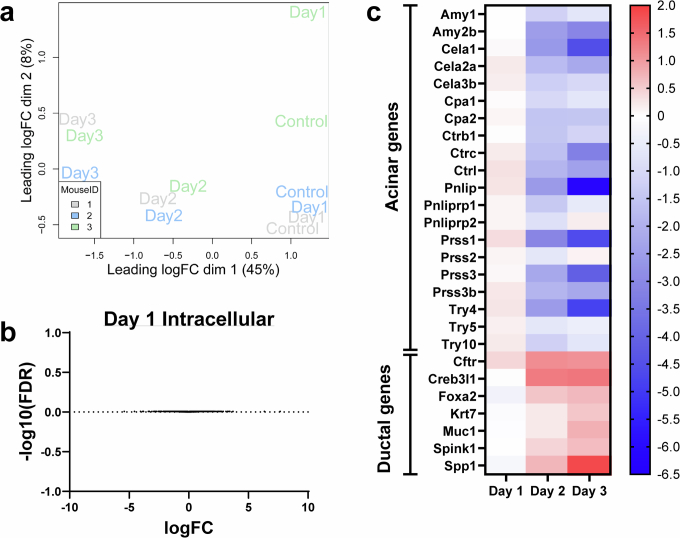
Continued summary of RNA-sequencing data. **a**. Multidimensional scaling plot of RNA-sequencing data from Vehicle (Control), Day 1, Day 2, and Day 3 cells treated with 4-OHT. Cells from each mouse are identified by color. **b**. Volcano plot of genes expressed in 4-OHT-treated Day 1 cells compared to Vehicle. There are no significantly differentially expressed genes. **c**. Heatmap of acinar and ductal gene expression. Scale represents log2FC in expression relative to Vehicle. Source data

**Extended Data Fig. 2:**
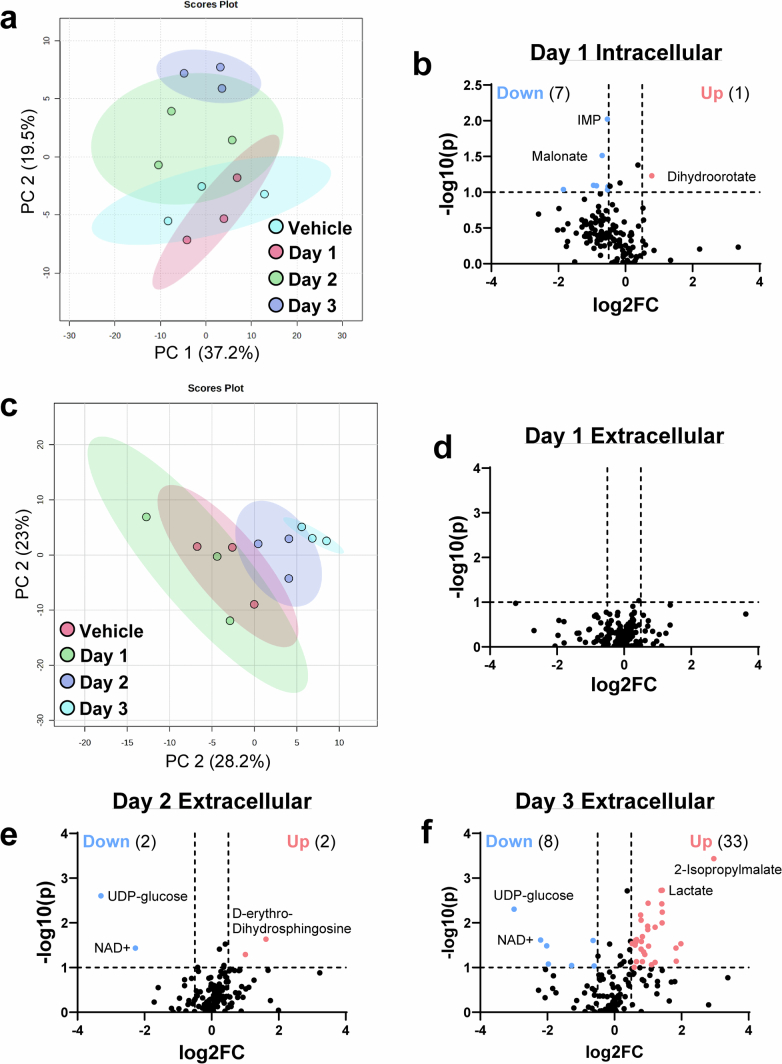
Continued summary of metabolomics data. **a**. Principal component analysis (PCA) plot of intracellular metabolites collected from Vehicle, or 4-OHT-treated Day 1, Day 2, and Day 3 cells generated using MetaboAnalyst. Cells from each mouse are identified by color. **b**. Volcano plots of differentially abundant intracellular metabolites when comparing Day 1 (4-OHT treated) to Vehicle. **c**. PCA plot of extracellular metabolites collected from Vehicle, Day 1, Day 2, or Day 3 4-OHT-treated cells generated using MetaboAnalyst. Cells from each mouse are identified by color. **d**. Volcano plots of differentially abundant extracellular metabolites when comparing Day 1 (4-OHT treated) to Vehicle. **e**. Volcano plots of differentially abundant extracellular metabolites when comparing Day 2 (4-OHT treated) to Vehicle. **f**. Volcano plots of differentially abundant extracellular metabolites when comparing Day 3 (4-OHT treated) to Vehicle. P-values for **b**, **d**, **e**, and **f** were calculated using a Student’s t-test (unpaired, two-tailed). P-value < 0.1 was considered significant, log2FC > 0.5 was considered increased, and log2FC < -0.5 was considered decreased. Significantly increased metabolites are labeled in red; decreased metabolites are labeled in blue. Source data

To investigate *Kras*^G12D^*-*driven metabolic changes during pancreatic cancer initiation, we performed RNA-seq and targeted liquid chromatography–tandem mass spectrometry (LC–MS/MS)-based metabolomics on primary, ex vivo cultured acinar cells undergoing ADM. We isolated acinar cells from LSL-*Kras*^G12D^; *Ptf1a*^CreERTM^ mice and treated them with vehicle or 4-hydroxytamoxifen (4-OHT) to induce mutant *Kras*^G12D^ (Fig. 1a). Cells were collected 1 day after vehicle treatment and 1, 2 and 3 days after 4-OHT treatment (days 1, 2 and 3). Multidimensional scaling of RNA-seq data demonstrated temporal transcriptional changes (Extended Data Fig. 1a). No differential gene expression was observed at day 1 of 4-OHT treatment compared to vehicle (Extended Data Fig. 1b), but a significant increase in upregulated and downregulated genes were observed on days 2 and 3 (Fig. 1b). Across the time course, a decrease in acinar genes with a concurrent increase in ductal genes was detected (Extended Data Fig. 1c). Principal component analysis of intracellular metabolites paralleled the transcriptomics changes (Extended Data Fig. 2a). Notably, the abundance of eight metabolites was significantly different at day 1 (Extended Data Fig. 2b), with a larger set of metabolites altered at days 2 and 3 (Fig. 1c). Metabolomics on culture media during the time course revealed complementary findings, with pronounced metabolic changes at days 2 and 3 (Extended Data Fig. 2c–f).

**Extended Data Fig. 3:**
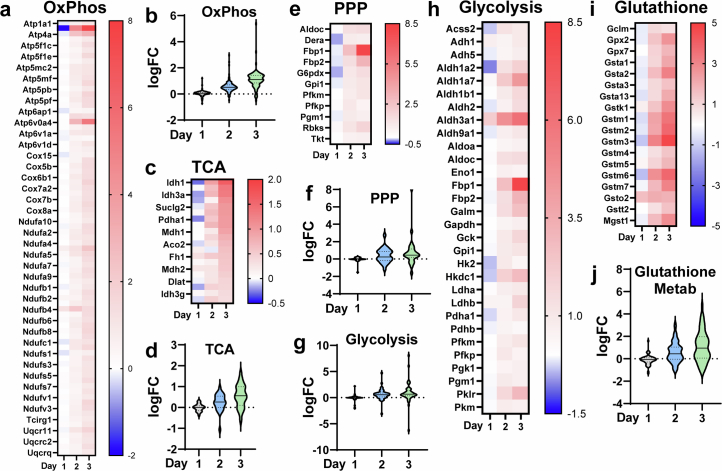
Heatmaps of leading edge genes and volcano plots of all the genes detected in respective pathways. **a**. Heatmap of the leading edge genes in the Oxidative Phosphorylation (OxPhos) KEGG pathway. Scale represents log2FC relative to Vehicle. **b**. Violin plot of the 106 detected genes in the OxPhos KEGG pathway. Y-axis represents log2FC relative to Vehicle. **c**. Heatmap of the leading edge genes in the tricarboxylic acid (TCA) cycle KEGG pathway. Scale represents log2FC relative to Vehicle. **d**. Violin plot of the 28 detected genes in the TCA cycle KEGG pathway. Y-axis represents log2FC relative to Vehicle. **e**. Heatmap of the leading edge genes in the Pentose Phosphate Pathway (PPP) KEGG pathway. Scale represents log2FC relative to Vehicle. **f**. Violin plot of the 27 detected genes in the PPP KEGG pathway. Y-axis represents log2FC relative to Vehicle. **g**. Violin plot of the 49 detected genes in the Glycolysis & Gluconeogenesis KEGG pathway. Y-axis represents log2FC relative to Vehicle. **h**. Heatmap of the edge genes in the Glycolysis & Gluconeogenesis KEGG pathway. Scale represents log2FC relative to Vehicle. **i**. Heatmap of the leading edge genes in the Glutathione Metabolism KEGG pathway. Scale represents log2FC relative to Vehicle. **j**. Violin plot of the 57 detected genes in the Glutathione Metabolism KEGG pathway. Y-axis represents log2FC relative to Vehicle. Source data

**Extended Data Fig. 4:**
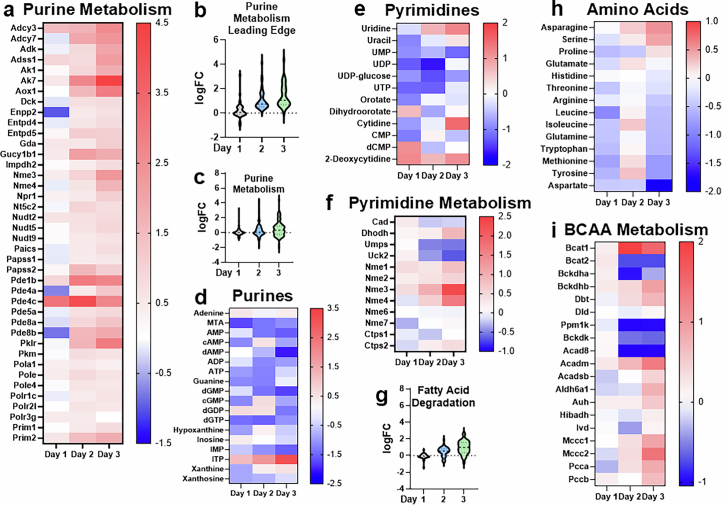
Other pathways of interest identified from RNAseq & Metabolomics. **a**. Heatmap of the leading edge genes in the Purine Metabolism KEGG pathway. Scale represents log2FC relative to Vehicle. **b**. Violin plot of the 40 leading edge genes in the Purine Metabolism KEGG pathway. Y-axis represents log2FC relative to Vehicle. **c**. Violin plot of the 93 detected genes in the Purine Metabolism KEGG pathway. Y-axis represents log2FC relative to Vehicle. **d**. Heatmap of detected purine-related metabolites. Scale represents log2FC of median normalized abundance relative to Vehicle. **e**. Heatmap of detected pyrimidine-related metabolites. Scale represents log2FC of median normalized abundance relative to Vehicle. **f**. Heatmap of genes that encode core enzymes in pyrimidine metabolism. Scale represents log2FC relative to Vehicle. **g**. Violin plot of the 38 detected genes in the Fatty Acid Degradation KEGG pathway. Y-axis represents log2FC relative to Vehicle. **h**. Heatmap of detected amino acid abundances. Scale represents log2FC of median normalized abundance relative to Vehicle. **i**. Heatmap of genes that encode core enzymes in branched-chain amino acid (BCAA) metabolism. Scale represents log2FC relative to Vehicle. Source data

To better understand transcriptional changes during ADM, we performed gene set enrichment analysis (Fig. 1d and Supplementary Figs. 1 and 2). Integration of RNA-seq and metabolomics data identified dynamic metabolic reprogramming during ADM. A decrease in tricarboxylic acid (TCA) cycle metabolites was observed at day 1 (Fig. 1e), consistent with a shift away from oxidative phosphorylation at the onset of ADM^18^. A sharp increase in alpha-ketoglutarate and succinate abundance was detected at day 2, remaining high at day 3 (Fig. 1e). Concurrently, at days 2 and 3, we saw a stepwise increase in expression of leading-edge genes included in the KEGG TCA cycle and oxidative phosphorylation gene sets (Fig. 1f,g and Extended Data Fig. 3a–d). Glycolysis and the PPP were broadly upregulated during ADM. However, ribose 5-phosphate, arabinose-5-phosphate and uridine diphosphate *N*-acetylglucosamine (UDP-GlcNAc) levels decreased in days 1–3 compared to vehicle (Fig. 1h). RNA-seq showed a stepwise increase in leading-edge genes from the PPP and glycolysis (Fig. 1i,j and Extended Data Fig. 3e–h). Extracellular metabolite profiling supported these findings, with a significant increase in lactate detected in the media (Extended Data Fig. 2f), suggesting increased glycolysis. Glutathione metabolism exhibited distinct temporal dynamics. We noted that reduced glutathione (GSH) peaked at day 1 with a reduction at days 2 and 3, whereas oxidized glutathione and other metabolites involved in glutathione synthesis increased by day 3 (Fig. 1k). There was a complementary, stepwise increase in leading-edge genes for glutathione metabolism during the time course (Fig. 1l and Extended Data Fig. 3i,j). Additionally, we observed a general upregulation of purine metabolism gene expression but an overall decrease in purine metabolites across timepoints (Extended Data Fig. 4a–d). There was variability in the levels of metabolites and expression of genes related to pyrimidine metabolism, and we observed a progressive increase in uracil, uridine and cytidine, with a decrease in uridine diphosphate, cytidine monophosphate and deoxycytidine monophosphate (Extended Data Fig. 4e,f). There was a cumulative increase in genes involved in fatty acid degradation (Extended Data Fig. 4g). Amino acid abundance exhibited a biphasic trend, generally low at days 1 and 3 but higher at day 2 (Extended Data Fig. 4h). Many genes involved in branched-chain amino acid metabolism were upregulated as ADM progressed (Extended Data Fig. 4i).

### An NRF2 pathway signature is present in mutant *Kras*-driven ADM

**Extended Data Fig. 5:**
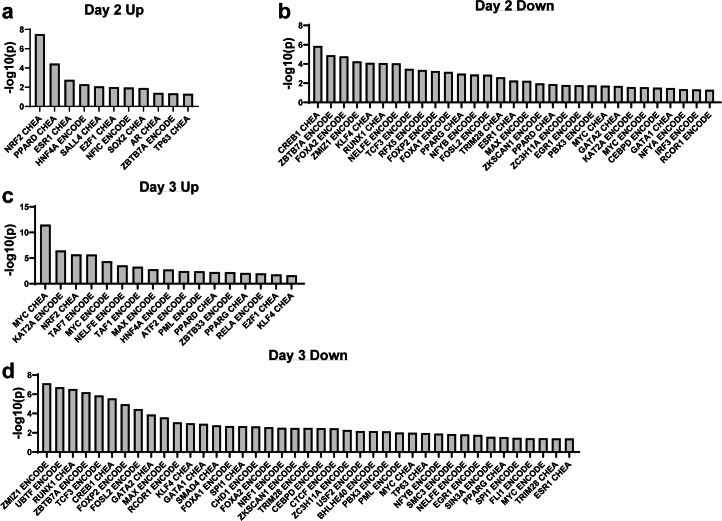
Enrichr transcription factor enrichment analysis. Transcription Factor Enrichment Analyses generated using Enrichr from ENCODE and ChEA Consensus Transcription Factors. **a**. Analysis from the top 500 upregulated genes at Day 2. **b**. Analysis from the top 500 downregulated genes at Day 2. **c**. Analysis from the top 500 upregulated genes at Day 3. **d**. Analysis from the top 500 downregulated genes at Day 3. Enrichr was used to identify differentially enriched pathways and calculate p-values with Fisher’s exact test. Source data

We performed transcription factor enrichment analysis using the Enrichr web tool^26–28^ (Fig. 1m and Extended Data Fig. 5). Consensus sites for NRF2 transcription factor binding were significantly enriched among the top 500 upregulated genes at day 2 (Fig. 1m and Extended Data Fig. 5a). NRF2 is known to have a key role in pancreatic tumourigenesis, progression and metastasis, in which it regulates expression of genes involved in ROS detoxification, cell metabolism, proliferation, invasion and migration^12,14,15,29,30^. We assayed our dataset for NRF2-target genes and found that several genes coding for NADPH-producing enzymes, such as *G6pdx* and *Me1*, increased during ADM formation (Fig. 1n and Supplementary Fig. 3a). We also observed protein expression from NRF2 targets G6PD, ME1 and NQO1 in mouse pancreas (Supplementary Fig. 3b–e).

**Extended Data Fig. 6:**
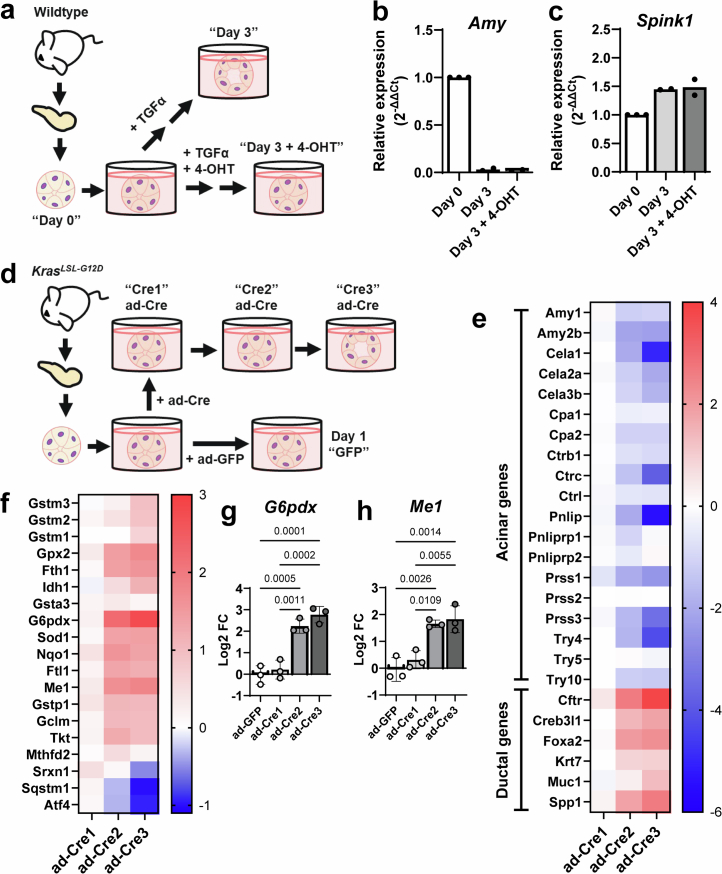
RNA analysis using additional ADM culture approaches. **a**. Scheme of the experimental model. Pancreata were dissected from Wildtype mice and acinar clusters were isolated for primary, *ex vivo* culture. Cells were collected after dissociation (Day 0) or grown free-floating in media and treated with TGFα (50 ng/mL), with or without 4-hydroxytamoxifen (4-OHT; 2 μM) for 3 days. Relative expression of acinar gene, *Amy* (**b**.) and ductal gene, *Spink1* (**c**.) using real-time quantitative PCR. Bars represent the mean of n = 3 biological replicates for Day 0, n = 2 biological replicates for Day 3 and Day 3 + 4-OHT. **d**. Scheme of the experimental model. Pancreata were dissected from LSL-Kras^G12D^ mice (n = 3) and acinar clusters were isolated for primary, *ex vivo* culture. Acinar clusters were grown free-floating in media and were treated with adenovirus expressing GFP (ad-GFP) or adenovirus expressing Cre recombinase (ad-Cre) to induce mutant *Kras*. Cells were collected after 1, 2, or 3 days in culture. **e**. Heatmap of acinar and ductal gene expression. Scale represents log2FC in expression relative to ad-GFP. **f**. Heatmap showing log2FC of key NRF2-target genes, as referenced from Fig. 1n. Individual datapoints for log2FC of gene expression of *G6pdx* (**g**.) and *Me1* (**h**.) from ADM cultures from each mouse (n = 3) after ad-Cre for 1, 2, or 3 days (relative to ad-GFP). Bars represent the mean with standard deviation. P-values were calculated using ordinary one-way ANOVA with Tukey’s multiple comparisons test. Source data

To assess whether 4-OHT treatment was nonspecifically affecting acinar cells, we cultured wild-type cells for 3 days with transforming growth factor-α (TGFα) to induce ADM, and administered 4-OHT or vehicle (Extended Data Fig. 6a). We observed that 4-OHT treatment alongside TGFα still reduced expression of *Amy* (acinar gene coding for amylase) and increased expression of *Spink1* (ductal gene coding for a trypsin inhibitor) (Extended Data Fig. 6b,c). We then checked whether an alternative ADM culture system yielded the same candidate genes. In this culture model, acinar cells from LSL-*Kras*^G12D^ mice were isolated and treated with adenovirus expressing green fluorescent protein (GFP) for 1 day or adenovirus expressing Cre recombinase for 1, 2 and 3 days (Extended Data Fig. 6d), after which cells were collected for RNA-seq^31^. We observed a concurrent decrease in acinar gene expression and an increase in ductal gene expression over the 3 days (Extended Data Fig. 6e), supporting the formation of ADM. We then assayed for the NRF2-target genes from Fig. 1n and observed the same patterns reproduced in this additional dataset (Extended Data Fig. 6f). Specifically, we saw a stepwise increase in *G6pdx* and *Me1* (Extended Data Fig. 6g,h), encoding NADPH-generating enzymes from the two major NADPH pathways in the cell. Collectively, data on ADM metabolism from these ex vivo profiling methods suggested an important role for NADPH regulatory enzymes G6PD and ME1.

### G6PD deficiency decreases oxidative PPP flux in *Kras*^G12D^-expressing acinar cells

**Fig. 2:**
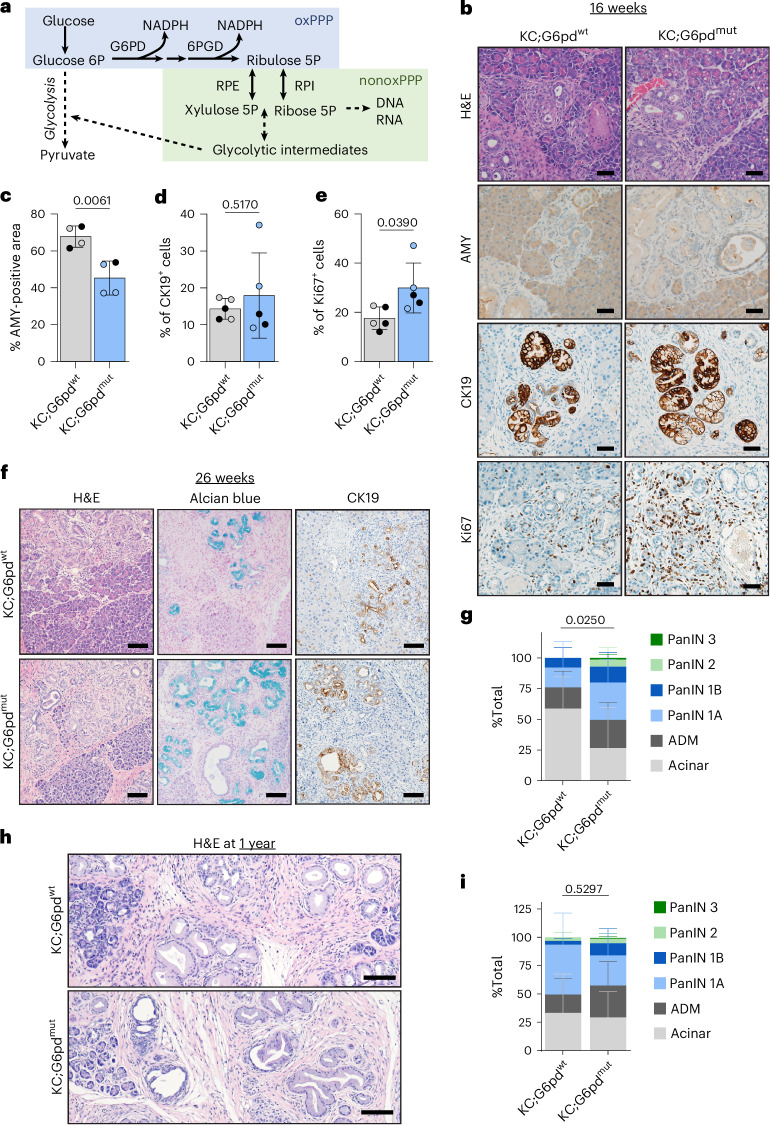
G6PD deficiency accelerates ADM and PanIN. **a**, Schematic of the PPP. In the first step of glycolysis, glucose is converted into glucose 6-phosphate (glucose 6P). G6PD, the first and rate-limiting enzyme of the oxidative PPP (oxPPP; blue), converts glucose 6P into 6-phosphogluconolactone and NADPH. Another NADPH-producing enzyme in the oxPPP, 6-phosphogluconate dehydrogenase (6PGD), generates ribulose 5-phosphate (ribulose 5P). Ribulose 5P can be used in the nonoxidative PPP (nonoxPPP; green) by the enzymes ribulose-5-phosphate 3-epimerase (RPE) and ribose-5-phosphate isomerase (RPI) to generate intermediate metabolites that enter glycolysis or nucleic acid biosynthesis, including ribose 5-phosphate (ribose 5P). **b**, H&E staining and immunostaining for amylase (AMY; acinar cells), cytokeratin 19 (CK19; ductal, metaplastic, neoplastic cells) and Ki67 (proliferation) in pancreata from 16-week-old KC;G6pd^wt^ and KC;G6pd^mut^ mice. Scale bars, 50 μm. **c**, Percentage of AMY-positive area in pancreata as quantified from male (closed circles) and female (open circles) KC;G6pd^wt^ and KC;G6pd^mut^ mice at 16 weeks; *n* = 4 mice for each genotype. Bars represent the mean values; error bars, s.d. *P* values were calculated using a Student’s *t*-test (unpaired, two-tailed). **d**,**e**, Percentage of CK19^+^ cells (**d**) and Ki67^+^ cells (**e**) in the pancreas as quantified from male (closed circles) and female (open circles) KC;G6pd^wt^ and KC;G6pd^mut^ mice at 16 weeks; *n* = 5 mice for each genotype. Bars represent the mean values; error bars, s.d. *P* values were calculated using a Student’s *t*-test (unpaired, two-tailed). **f**, H&E staining, Alcian Blue staining (for PanIN-produced mucins) and CK19 (ductal, metaplastic, neoplastic cells) immunostaining in 26-week-old KC;G6pd^wt^ and KC;G6pd^mut^ mice. Scale bars, 100 μm. **g**, Pathological grading of pancreas tissues from 26-week-old KC;G6pd^wt^ and KC;G6pd^mut^ mice, representing the percentage of total tissue area with acinar cells, ADM and PanIN lesions; *n* = 5 KC;G6pd^wt^ mice; *n* = 7 KC;G6pd^mut^ mice. Bars represent the mean values; error bars, s.d. *P* values were calculated using a two-way ANOVA with Tukey’s post hoc test. **h**, H&E staining in 1-year-old KC;G6pd^wt^ and KC;G6pd^mut^ mice. Scale bars, 100 μm. **i**, Pathological grading of pancreas tissues from 1-year-old KC;G6pd^wt^ and KC;G6pd^mut^ mice, representing the percentage of total tissue area with acinar cells, ADM and PanIN lesions; *n* = 5 mice for each genotype. Bars represent the mean values; error bars, s.d. *P* values were calculated using a two-way ANOVA with Tukey’s post hoc test. Source data

**Extended Data Fig. 7:**
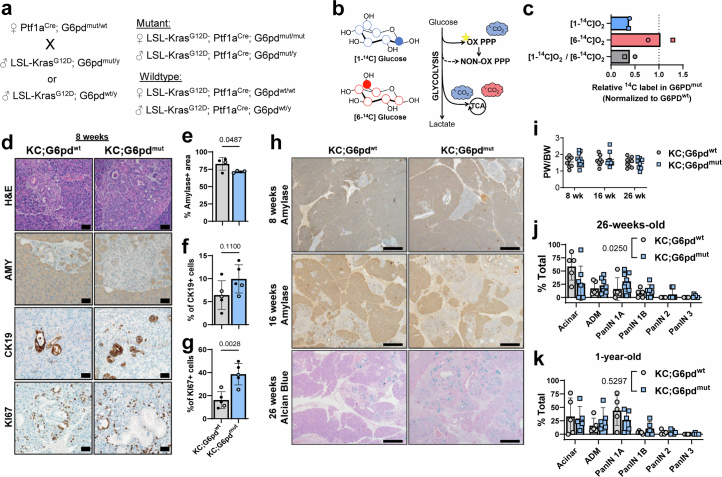
Additional description of and analyses in KC;G6pd^wt^ and KC;G6pd^mut^ mice. **a**. Mice with mutant *G6pd*, that mimics human G6PD-deficiency, were bred into the KC (*LSL-Kras*^*G12D*^*; Ptf1a*^*Cre*^*)* line. *Cre* was always maintained in female breeders. Experiments used both male and female *G6pd* mutant mice, where females were homozygous for mutant *G6pd* (*G6pd*^*mut/mut*^) and males were hemizygous for mutant *G6pd* (*G6pd*^*mut/y*^), as *G6pd* is an X-linked gene. *G6pd* wildtype mice used in experiments were obtained in the same colony and age matched littermates of *G6pd* mutant mice when possible. Female *G6pd* wildtype mice have two wildtype copies of *G6pd* (*G6pd*^*wt/wt*^) and males have one wildtype copy (their only copy) of *G6pd* (*G6pd*^*wt/y*^). In the schematics and labelling, “y” in male mice refers to the y chromosome, which does not contain a copy of *G6pd*. **b**. Schematic of ^14^C-labeling experiment. [1-^14^C]glucose (blue) can be used in both the oxidative pentose phosphate pathway (ox PPP) and the TCA cycle. [6-^14^C]glucose (red) can be used in the TCA cycle. Released CO_2_ represented as clouds; G6PD mutation represented as star. **c**. Graph depicting the relative amount of [^14^C]-labeled CO_2_ derived from glucose tracing in G6pd^mut^ mice relative to G6pd^wt^ mice (n = 2 biological replicates). Blue bar shows the relative [^14^C]-labeled CO_2_ derived from [1-^14^C]glucose, which is generated from either the oxidative pentose phosphate pathway (oxPPP) or the TCA cycle. Red bar shows the relative amount of [^14^C]-labeled CO_2_ derived from [6-^14^C]glucose, which is only generated from the TCA cycle. Grey bar shows the ratio of [^14^C]-labeled CO_2_ derived from [1-^14^C]O_2_ to [6-^14^C]O_2_ representing flux from the oxPPP. Bars represent the biological replicates’ mean. **d**. Hematoxylin & eosin (H&E) staining and immunostaining for amylase (AMY; acinar cells), cytokeratin 19 (CK19; ductal, metaplastic, neoplastic cells), and KI67 (proliferation) in pancreata from 8-week-old KC;G6pd^wt^ and KC;G6pd^mut^ mice. Scale bar = 50μm. **e**. Percent of Amylase+ area in pancreas as quantified from male (closed circle) and female (open circle) mice in KC;G6pd^wt^ (grey bar) and KC;G6pd^mut^ (blue bar) mice at 8 weeks. n = 4 mice for each genotype. Bars represent the mean with standard deviation. P-values were calculated using a Student’s t-test (unpaired, two-tailed). **f**. Percent of CK19+ cells and **g**. Ki67+ cells in the pancreas as quantified from male (closed circle) and female (open circle) KC;G6pd^wt^ (grey bar) and KC;G6pd^mut^ (blue bar) mice at 8 weeks. n = 5 mice for each genotype. Bars represent the mean with standard deviation. P-values were calculated using a Student’s t-test (unpaired, two-tailed). **h**. Immunostaining for Amylase (AMY), in 8-week and 16-week-old KC;G6pd^wt^ and KC;G6pd^mut^ pancreas. Alcian blue (PanIN-produced mucin) & nuclear fast red counterstain in pancreas of 26-week-old KC;G6pd^wt^ and KC;G6pd^mut^ mice. Scale bar = 500μm. **i**. Pancreas weight (PW) to body weight (BW) ratios in 8-, 16-, and 26-week-old KC;G6pd^wt^ and KC;G6pd^mut^ mice. Bars represent the mean with standard deviation. Ratios are not significantly different, as calculated using a Student’s t-test (unpaired, two-tailed) for each age. Sample sizes for each age are as follows: 8 week, n = 11 KC;G6pd^wt^ and n = 8 KC;G6pd^mut^; 16 week, n = 9 KC;G6pd^wt^ and n = 8 KC;G6pd^mut^; 26 week, n = 8 KC;G6pd^wt^ and n = 8 KC;G6pd^mut^. **j**. Pathological grading of 26-week-old KC;G6pd^wt^ and KC;G6pd^mut^ pancreas tissues representing the % of total tissue area with acinar cells, ADM, and PanIN lesions. n = 5 KC;G6pd^wt^ mice; n = 7 KC;G6pd^mut^ mice. Bars represent the mean with standard deviation. P-values were calculated using a two-way ANOVA with Tukey’s multiple comparisons test. **k**. Pathological grading of 1-year-old KC;G6pd^wt^ and KC;G6pd^mut^ pancreas tissues representing the % of total tissue area with acinar cells, ADM, and PanIN lesions. n = 5 mice per genotype. Bars represent the mean with standard deviation. P-values were calculated using a two-way ANOVA with Tukey’s multiple comparisons test. Source data

The PPP branches from glycolysis and consists of two arms: oxidative and nonoxidative (Fig. 2a). Flux through the oxidative arm, where G6PD resides, yields NADPH and ribose for nucleotides. Given the significant increase of *G6pdx* in ADM (Fig. 1n, Extended Data Fig. 6f,g and Supplementary Fig. 3a) and its contrast with activation of the nonoxidative PPP in PDAC, we sought to determine the role of oxidative PPP metabolism in ADM. A mouse model of human G6PD deficiency demonstrates enzyme activity of 15–20% in hemizygous mice (male *G6pd*^mut/y^), 50–60% in heterozygous mice (female *G6pd*^mut/+^) and 15–20% in homozygous mutant mice (female *G6pd*^mut/mut^)^32,33^. To determine how G6PD deficiency and decreased oxidative PPP flux alters pancreatic tumourigenesis, we generated LSL*-Kras*^G12D^; *Ptf1a*^Cre^; *G6pd*^mut^ mice (KC;G6pd^mut^) (breeding scheme in Extended Data Fig. 7a).

To determine whether oxidative PPP activity was decreased in G6pd^mut^ pancreas, we traced radioactive carbon incorporation into carbon dioxide (CO_2_) produced from oxidative decarboxylation in the PPP and TCA cycle. [1-^14^C]glucose labels CO_2_ derived from both oxidative PPP and the TCA cycle, while [6-^14^C]glucose specifically traces TCA-derived CO_2_. By following the tracers in tandem, we can determine oxidative PPP activity (Extended Data Fig. 7b). As expected, KC;G6pd^mut^ acinar cells have decreased oxidative PPP flux, as measured by [1-^14^C]O_2_, compared to G6pd^wt^ cells (Extended Data Fig. 7c). TCA cycle flux was not affected by *G6pd* status, as measured by [6-^14^C]O_2_ (Extended Data Fig. 7c). These data suggest that G6pd^mut^ acinar cells have less oxidative PPP activity, establishing a physiologically relevant model to study how G6PD deficiency affects pancreatic tumourigenesis.

### G6PD deficiency accelerates ADM and PanIN lesions in mouse models

ROS generated during pancreatic tumourigenesis is countered by flux into the PPP to provide NADPH^13,14,22^. We reasoned that decreasing oxidative PPP activity through G6PD deficiency would promote *Kras*^G12D^-driven ADM and tumourigenesis owing to increased oxidative stress. To test this hypothesis, we aged KC;G6pd^mut^ and KC;G6pd^wt^ mice to 8 weeks, when ADM is present within the pancreas of KC mice. We observed ADM in KC;G6pd^wt^ mice as noted by haematoxylin and eosin (H&E)-stained tissue sections, decreased amylase (AMY), increased cytokeratin 19 (CK19) and increased proliferation (Ki67) within ADM lesions (Extended Data Fig. 7d). In KC;G6pd^mut^ mice, there was a trend for less acinar cell area and significantly increased proliferation (Extended Data Fig. 7e–h). At 16 weeks, when ADM and early PanIN lesions are present in KC mice, KC;G6pd^mut^ mice showed more ADM and PanIN lesions, indicated by a significant decrease in AMY and significantly more proliferation compared to KC;G6pd^wt^ mice (Fig. 2b–e and Extended Data Fig. 7h). We did not find a difference in pancreas weight to body weight ratios in KC;G6pd^mut^ mice, an indirect measure of tissue transformation (Extended Data Fig. 7i).

To determine the impact of G6PD deficiency on PanIN formation, we aged mice to 26 weeks, when low-grade PanINs are present in KC models (Fig. 2f). There was no difference in pancreas weight to body weight ratios in 26-week-old mice (Extended Data Fig. 7i). We observed PanINs present in pancreata from both KC;G6pd^wt^ and KC;G6pd^mut^ mice, as assessed by CK19 expression and Alcian Blue stain for PanIN-specific mucins (Fig. 2f and Extended Data Fig. 7h). Pathological grading of H&E-stained tissues demonstrated more transformed area in KC;G6pd^mut^ mice (Fig. 2g and Extended Data Fig. 7j). Additionally, tissues from KC;G6pd^mut^ mice contained more low-grade PanIN lesions (PanIN 1A, 1B, 2) (Fig. 2g and Extended Data Fig. 7j). Interestingly, two KC;G6pd^mut^ mice displayed high-grade lesions (PanIN 3), which were absent in KC;G6pd^wt^ mice. Pancreata from KC;G6pd^mut^ mice also exhibited less acinar cell area and more ADM. We aged mice to 1 year to determine whether the high-grade PanIN lesions present in KC;G6pd^mut^ mice progress to PDAC (Fig. 2h,i and Extended Data Fig. 7k). However, we saw no significant difference in pancreata between KC;G6pd^wt^ and KC;G6pd^mut^ mice after pathological grading of H&E-stained tissues (Fig. 2i and Extended Data Fig. 7k).

Overall, these data illustrate that G6PD deficiency in *Kras*^G12D^-driven mouse models increased the number and rate of precancerous lesion formation in 16-week-old and 26-week-old animals. However, after 1 year, our results suggest that G6PD deficiency does not affect PanIN to PDAC progression.

### G6PD deficiency does not affect survival in the PDAC mouse model

We next assessed the impact on pancreatic cancer progression and survival using the more rapid KPC mouse model (LSL-*Kras*^G12D^; LSL-*Trp53*^R172H^; *Ptf1a*^Cre^)^34^. KPC mice were crossed to G6PD-deficient mice to determine whether accelerated ADM and PanIN lesions enhanced invasive PDAC and decreased survival (breeding scheme in Supplementary Fig. 4a). There was no significant difference in overall survival between KPC;G6pd^mut^ and KPC;G6pd^wt^ mice (Supplementary Fig. 4b). We assessed histology from pancreas of 90-day-old mice and observed that both genotypes had neoplasia, with significantly increased proliferation in pancreata from KPC;G6pd^mut^ mice (Supplementary Fig. 4c–e). We did not see evidence of metastasis to the liver (Supplementary Fig. 4f), and there was no difference in pancreas weight to body weight ratios (Supplementary Fig. 4g). There was also no significant difference in pathological tissue grading of pancreas from 90-day-old KPC;G6pd^mut^ and KPC;G6pd^wt^ mice (Supplementary Fig. 4h). Although G6PD deficiency promotes precancerous lesions in KC mice, these data indicate that G6PD deficiency does not affect progression to cancer or survival in KPC mice, highlighting a difference in the metabolic pathways that underlie precancer and established cancer.

### *Me1* loss accelerates ADM, PanIN and PDAC in mouse models

**Fig. 3:**
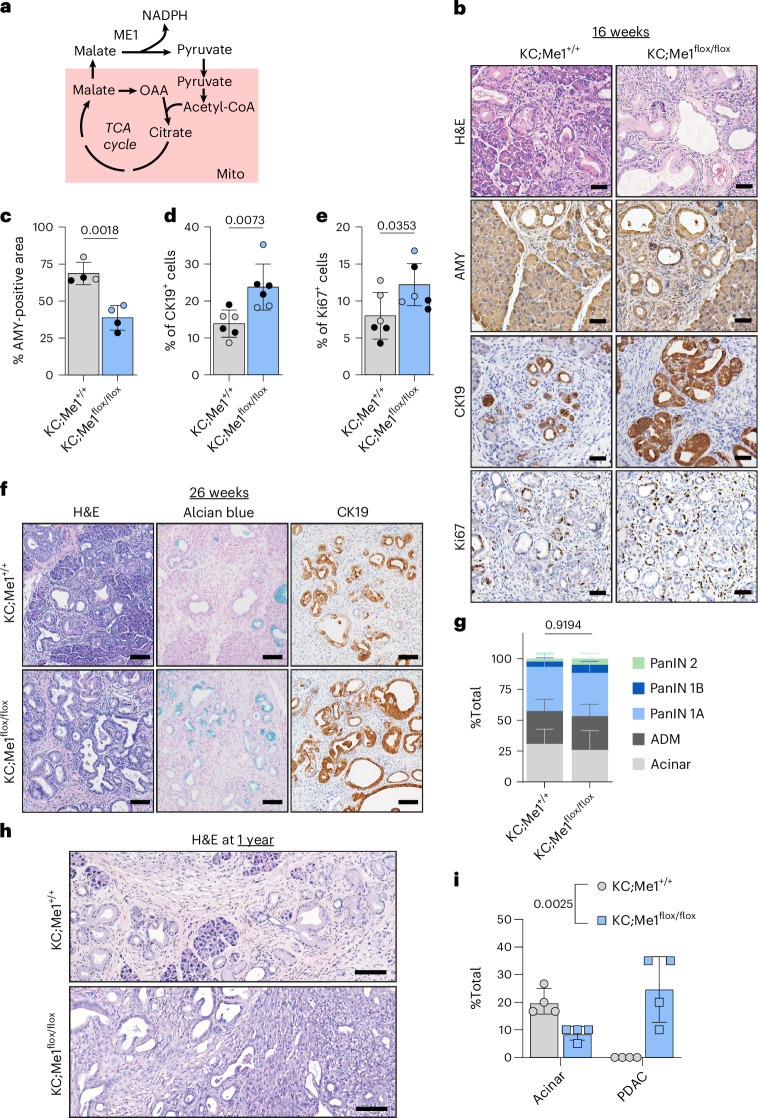
*Me1* loss accelerates the formation of early precursor lesions and PDAC progression. **a,** Schematic of malic enzyme 1 (ME1) function and related metabolic pathways. ME1 is a cytosolic enzyme that converts malate to pyruvate, generating NADPH in the process. Malate can come from the cytosol or be transported across the mitochondrial membrane from the TCA cycle. The pyruvate generated by ME1 can enter the TCA cycle. Mitochondria (Mito) presented in pink. **b**, H&E staining and immunostaining for AMY (acinar cells), CK19 (ductal, metaplastic, neoplastic cells) and Ki67 (proliferation) in pancreas of 16-week-old KC;Me1^+/+^ and KC;Me1^flox/flox^ mice. Scale bars, 50 μm. **c**, Percentage of AMY-positive area in pancreas as quantified from male (closed circles) and female (open circles) KC;Me1^+/+^ and KC;Me1^flox/flox^ mice at 16 weeks; *n* = 4 mice for each genotype. Bars represent the mean values; error bars, s.d. *P* values were calculated using a Student’s *t*-test (unpaired, two-tailed). **d**,**e**, Percentage of CK19^+^ cells (**d**) and Ki67^+^ cells (**e**) in the pancreas as quantified from male (closed circles) and female (open circles) KC;Me1^+/+^ and KC;Me1^flox/flox^ mice at 16 weeks; *n* = 6 mice for each genotype. Bars represent the mean values; error bars, s.d. *P* values were calculated using a Student’s *t*-test (unpaired, two-tailed). **f**, H&E staining, Alcian Blue staining (for PanIN-produced mucins) and CK19 (ductal, metaplastic, neoplastic cells) immunostaining in 26-week-old KC;Me1^+/+^ and KC;Me1^flox/flox^ mice. Scale bars, 100 μm. **g**, Pathological grading of pancreas tissues from 26-week-old KC;Me1^+/+^ and KC;Me1^flox/flox^ mice, representing the percentage of total tissue area with acinar cells, ADM and PanIN lesions; *n* = 4 mice for each genotype. Bars represent the mean values; error bars, s.d. *P* values were calculated using a two-way ANOVA with Tukey’s post hoc test. **h**, H&E staining in 1-year-old KC;Me1^+/+^ and KC;Me1^flox/flox^ mice. Scale bar, 100 μm. **i**, Pathological grading of pancreas tissues from 1-year-old KC;Me1^+/+^ and KC;Me1^flox/flox^ mice, representing the percentage of total tissue area with acinar cells and PDAC; *n* = 4 mice for each genotype. Bars represent the mean values; error bars, s.d. *P* values were calculated using a two-way ANOVA with Tukey’s post hoc test. Source data

**Extended Data Fig. 8:**
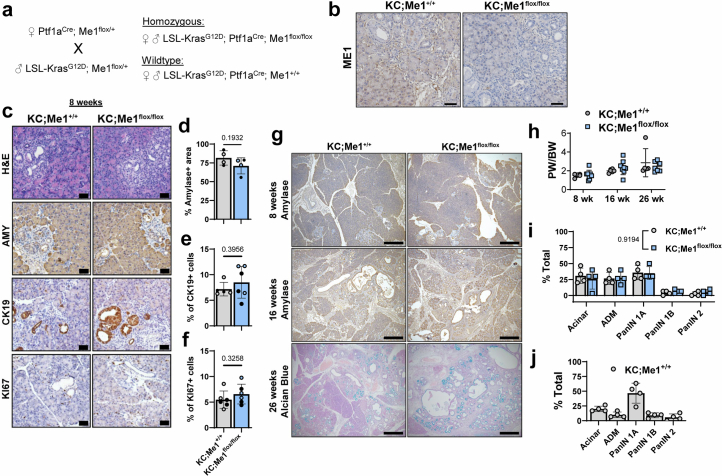
Additional description of and analyses in KC;Me1^+/+^ and KC;Me1^flox/flox^ mice. **a**. Mice with *Me1*^*flox/flox*^ alleles were bred into the KC (*LSL-Kras*^*G12D*^*; Ptf1a*^*Cre*^) line. *Cre* was always maintained in female breeders. Experiments used both male and female mice that were homozygous for the *Me1*^*flox/flox*^ allele and mice that were *Me1*^*+/+*^ (wildtype). **b**. Immunostaining for ME1 in 16-week-old KC;Me1^+/+^ and KC;Me1^flox/flox^ pancreas. Scale bar = 50μm. **c**. Hematoxylin & eosin (H&E) staining and immunostaining for amylase (AMY; acinar cells), cytokeratin 19 (CK19; ductal, metaplastic, neoplastic cells), and Ki67 (proliferation) in pancreas of 8-week-old KC;Me1^+/+^ and KC;Me1^flox/flox^ mice. Scale bar = 50μm. **d**. Percent of Amylase+ area in pancreas as quantified from male (closed circle) and female (open circle) mice in KC;Me1^+/+^ (grey bar) and KC;Me1^flox/flox^ (blue bar) mice at 8 weeks. n = 4 mice for each genotype. Bars represent the mean with standard deviation. P-values were calculated using a Student’s t-test (unpaired, two-tailed). **e**. Percent of CK19+ cells and **f**. Ki67+ cells in the pancreas as quantified from male (closed circle) and female (open circle) KC;Me1^+/+^ (grey bar) and KC;Me1^flox/flox^ (blue bar) mice at 8 weeks. In CK19 plot: n = 5 KC;Me1^+/+^ and n = 6 KC;Me1^flox/flox^ mice. In Ki67 plot: n = 6 mice for each genotype. Bars represent the mean with standard deviation. P-values were calculated using a Student’s t-test (unpaired, two-tailed). **g**. Immunostaining for Amylase (AMY), in 8-week and 16-week-old KC;Me1^+/+^ and KC;Me1^flox/flox^ pancreas. Alcian blue (PanIN-produced mucin) & nuclear fast red counterstain in pancreas of 26-week- KC;Me1^+/+^ and KC;Me1^flox/flox^ mice. Scale bar = 500μm. **h**. Pancreas weight (PW) to body weight (BW) ratios in 8-, 16-, and 26-week-old KC;Me1^+/+^ and KC;Me1^flox/flox^ mice. Bars represent the mean with standard deviation. Ratios are not significantly different as calculated using a Student’s t-test (unpaired, two-tailed) for each age. Sample sizes for each age are as follows: 8 week, n = 6 KC;Me1^+/+^ and n = 8 KC;Me1^flox/flox^; 16 week, n = 6 KC;Me1^+/+^ and n = 8 KC;Me1^flox/flox^; 26 week, n = 5 KC;Me1^+/+^ and n = 7 KC;Me1^flox/flox^. **i**. Pathological grading of 26-week-old KC;Me1^+/+^ and KC;Me1^flox/flox^ pancreas tissues representing the % of total tissue area with acinar cells, ADM, and PanIN lesions. n = 4 mice per genotype. Bars represent the mean with standard deviation. P-values were calculated using a two-way ANOVA with Tukey’s multiple comparisons test. **j**. Pathological grading of 1-year-old KC;Me1^+/+^ pancreas tissues representing the % of total tissue area with acinar cells, ADM, and PanIN lesions. n = 4 mice. Bars represent the mean with standard deviation. Source data

We next sought to determine the role of ME1 in *Kras*-driven pancreatic carcinogenesis. ME1 is a cytoplasmic enzyme that catalyses the reversible oxidative decarboxylation of malate to pyruvate, generating NADPH (schematic in Fig. 3a). Similar to *G6pdx*, *Me1* was increased in a temporal manner in the primary acinar cultures (Fig. 1n and Extended Data Fig. 6f–h). To assess the necessity of *Me1* in pancreatic tumourigenesis, we crossed *Me1*^flox/flox^ mice^35^ into the KC model, generating KC;Me1^flox/flox^ mice, in which oncogenic *Kras*^G12D^ and *Me1* loss are induced in the pancreas by *Ptf1a*^Cre^ (breeding scheme in Extended Data Fig. 8a). ME1 protein expression was reduced in pancreata from KC;Me1^flox/flox^ mice (Extended Data Fig. 8b), demonstrating knockout efficiency in the pancreas.

At 8-weeks-old, there was no significant difference in ADM lesions present in KC;Me1^flox/flox^ mice compared to KC;Me1^+/+^ controls (Extended Data Fig. 8c–g). However, at 16 weeks, more ADM and PanIN lesions were present in KC;Me1^flox/flox^ mice, as indicated by significantly decreased AMY, increased CK19 and increased Ki67 (Fig. 3b–e). No significant difference in pancreas weight to body weight ratios were observed at 8 weeks and 16 weeks (Extended Data Fig. 8h). Precancerous lesions are found in pancreata of 26-week-old KC;Me1^+/+^ and KC;Me1^flox/flox^ mice, as observed by H&E, Alcian blue and CK19 staining (Fig. 3f and Extended Data Fig. 8g). Yet there was no significant difference in pathological grading (Fig. 3g and Extended Data Fig. 8i). We also did not find significant differences in pancreas weight to body weight ratios (Extended Data Fig. 8h). To determine whether *Me1* loss influences progression to PDAC, we aged mice to 1 year and assessed pancreas histology by H&E staining and pathological grading (Fig. 3h,i). Surprisingly, all KC;Me1^flox/flox^ mice developed PDAC (Fig. 3i), while none of the KC;Me1^+/+^ controls progressed past PanIN 2 lesions (Extended Data Fig. 8j). Additionally, KC;Me1^flox/flox^ pancreata had a smaller acinar cell area (Fig. 3i).

Overall, ADM and PanIN lesions are more abundant and occur sooner (at 16 weeks) in pancreata of KC;Me1^flox/flox^ mice than in KC;Me1^+/+^ controls. These data suggest that *Me1* loss, consistent with G6PD deficiency, promotes precancerous lesions in *Kras*^G12D^-driven mouse models. However, we also observe no significant effect of *Me1* loss at 26 weeks but a unique promotion of PDAC at 1 year. Together, these results reveal common and diverging roles of NADPH-generating enzymes at distinct stages of tumourigenesis and progression.

### G6PD deficiency and *Me1* loss do not alter pancreatic endocrine and exocrine function

We sought to determine whether impaired function of NADPH-producing enzymes disrupts normal pancreas physiology, which could predispose to precancerous lesion formation^36,37^. We performed glucose tolerance tests on *Me1*^+/+^, *Me1*^−/−^, wild-type, KC;G6pd^wt^ and KC;G6pd^mut^ mice after an overnight fast (Supplementary Fig. 5a–h). There were no significant differences in glucose tolerance across genotypes, although previous studies show that hemizygous G6PD-deficient male mice have smaller islets and impaired glucose tolerance^38^. The only significant change we detected was a reduction in fasted body weight in *Me1*^−/−^ mice compared to *Me1*^+/+^ controls (Supplementary Fig. 5c). We also tested exocrine pancreas function through serum amylase activity tests, using cerulein treatment as a positive control (Supplementary Fig. 5i). We did not observe significant differences in serum amylase activity across groups compared to wild-type mice. Finally, we checked for premature carboxypeptidase A1 (CPA1) enzyme activation (Supplementary Fig. 5j). Cerulein-treated positive controls showed cleavage of the CPA1 pro-peptide^39^, but this was not observed in the other mice.

### Increased ROS is present with G6PD deficiency and *Me1* loss

We used mass spectrometry to assess NADPH, NADP^+^ and glutathione levels in 16-week-old pancreas tissue. The NADPH/NADP^+^ ratios trended toward less cellular reducing capacity in pancreata of KC;G6pd^mut^ and KC;Me1^flox/flox^ mice relative to controls but did not reach statistical significance, and there were no significant differences in glutathione levels (Supplementary Fig. 6a–l). These results demonstrate the critical requirement for tissues to maintain NADPH and antioxidants, and sufficient compensation by other metabolic pathways.

**Fig. 4:**
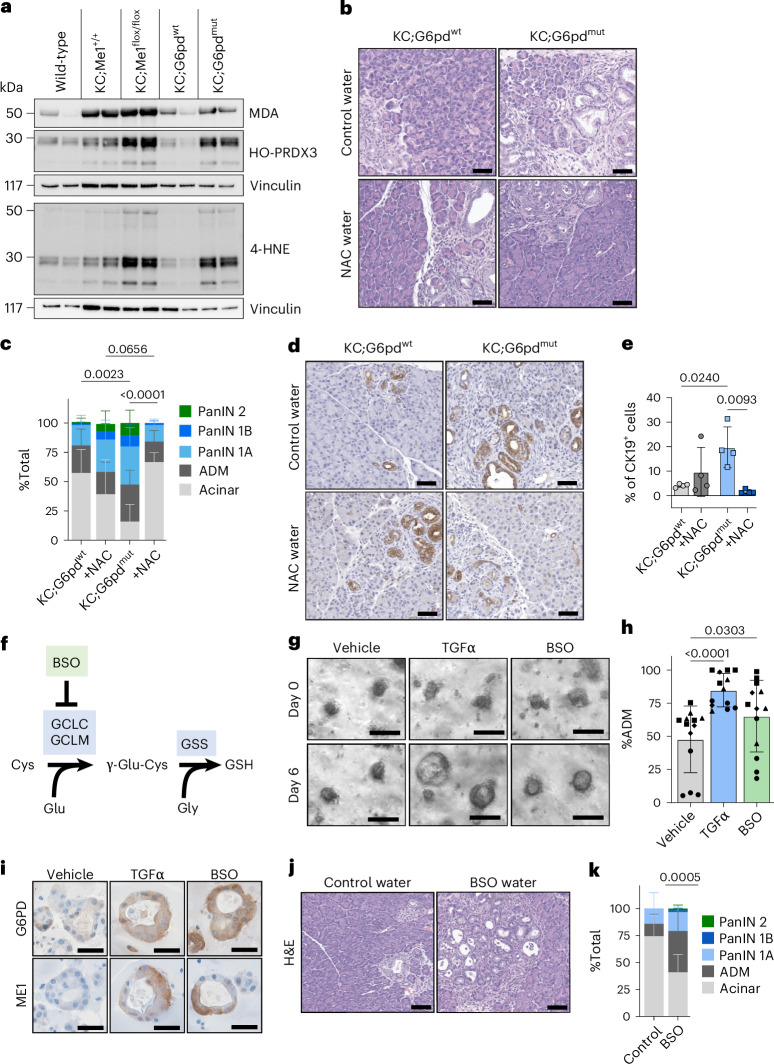
Antioxidants regulate precancerous lesion formation. Western blots for lipid peroxidation markers malondialdehyde (MDA), hyperoxidized peroxiredoxin 3 (HO-PRDX3) and 4-hydroxynonenal (4-HNE) normalized to Vinculin loading control. Samples are pancreas lysates (*n* = 2 per genotype). Western blot was repeated two independent times. **b**, H&E staining of pancreas from 11–12-week-old KC;G6pd^wt^ and KC;G6pd^mut^ mice after administration of control or *N*-acetylcysteine (NAC; 30 mM)-containing water for 5–6 weeks. Scale bars, 50 μm. **c**, Pathological grading of pancreas tissues from KC;G6pd^wt^ and KC;G6pd^mut^ mice from **b** representing the percentage of total tissue area with acinar cells, ADM and PanIN; *n* = 4 mice for each genotype. Bars represent the mean values; error bars, s.d. *P* values were calculated using a two-way ANOVA with multiple comparisons. Where *P* < 0.0001, the exact value is 1.0 × 10^−5^. **d**, Immunostaining for CK19 (ductal, metaplastic, neoplastic cells) in pancrease of 11–12-week-old KC;G6pd^wt^ and KC;G6pd^mut^ mice after administration of untreated or *N*-acetylcysteine water for 5–6 weeks. Scale bars, 50 μm. **e**, Percentage of CK19^+^ cells in the pancreas as quantified from male (closed circles) and female (open circles) KC;G6pd^wt^ and KC;G6pd^mut^ mice in **d**; *n* = 4 mice for each genotype. Bars represent the mean values; error bars, s.d. *P* values were calculated using ordinary one-way ANOVA with Tukey’s multiple comparisons test. **f**, Schematic of de novo glutathione synthesis. Intracellular cysteine (Cys) is converted to glutathione (GSH). The rate-limiting enzyme glutamate cysteine ligase (GCL) is made up of a catalytic subunit (GCLC) and a regulatory, modifier subunit (GCLM). GCL catalyses the condensation of cysteine and glutamate (Glu) to the dipeptide precursor γ-glutamylcysteine (γ-Glu-Cys). Glutathione synthetase (GSS) catalyses the condensation of γ-Glu-Cys and glycine (Gly) to GSH. Buthionine sulfoximine (BSO) inhibits GCL. **g**, Brightfield images of primary acinar cell cultures isolated from human donor pancreata at day 0 and day 6. Cells were treated with vehicle (DMSO), TGFα (50 ng ml^−1^) or BSO (100 μM). Scale bars, 100 μm. **h**, Quantification of acinar cells undergoing ADM in primary cultures at day 6 in vehicle-treated, TGFα-treated and BSO-treated cells. Each symbol shape represents technical duplicates from a unique donor (*n* = 4 donor pancreata). Bars represent the mean values; error bars, s.d. *P* values were calculated using repeated measures one-way ANOVA with Dunnett’s multiple comparisons test. Where *P* < 0.0001, the exact value is 6.1 × 10^−5^. **i**, Representative immunostaining for G6PD and ME1 from human ADM primary cultures at day 6. Scale bars, 25 μm. **j**, H&E staining in pancreata from 5–6-week-old KC mice administered control or BSO-containing water for 5 weeks. Scale bars, 50 μm. **k**, Pathological grading of pancreata from 5–6-week-old KC mice administered control or BSO-containing water for 5 weeks. Datapoints represent the percentage of total tissue area with acinar cells, ADM and PanIN lesions; *n* = 4 mice for each treatment. Bars represent the mean values; error bars, s.d. *P* values were calculated using a two-way ANOVA with Tukey’s multiple comparisons test. Source data

**Extended Data Fig. 9:**
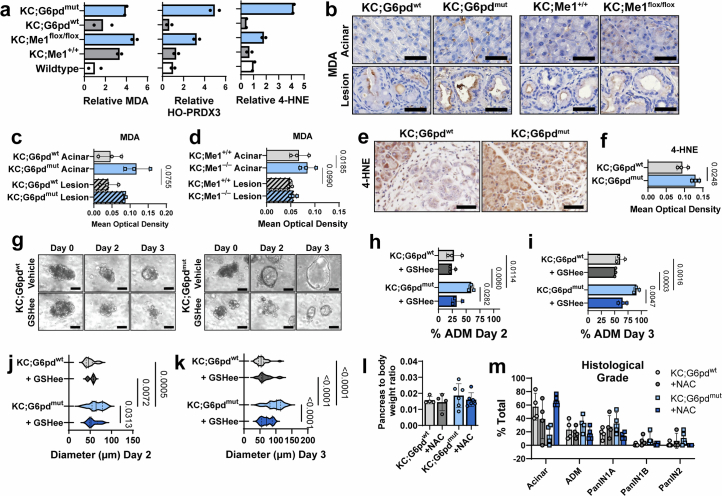
ROS markers in pancreas tissue and acinar cell cultures with antioxidant treatment. **a**. Quantification of protein levels from Fig. 4a with samples normalized to Vinculin and relative to Wildtype expression. n = 2 per genotype. The experiment was repeated twice. MDA = Malondialdehyde; HO-PRDX3 = Hyperoxidized Peroxiredoxin 3; 4-HNE = 4-hydroxynonenal. **b**. Immunostaining for MDA in pancreas of 16-week-old KC;G6pd^wt^, KC;G6pd^mut^, KC;Me1^+/+^, and KC;Me1^flox/flox^ mice, presented for acinar and lesion regions. Scale bar = 50μm. **c-d**. Quantification of mean optical density from MDA immunostaining in mouse pancreas separated by expression in acinar or lesion regions of the tissue. Each datapoint represents one mouse. n = 3 mice per genotype. Bars represent the mean with standard deviation. P-values were calculated using repeated measures one-way ANOVA with Tukey’s multiple comparisons test. **e**. Immunostaining for 4-HNE in pancreas of 16-week-old KC;G6pd^wt^ and KC;G6pd^mut^ mice Scale bar = 50μm. **f**. Quantification of mean optical density from 4-HNE immunostaining. Each datapoint represents one mouse. n = 3 mice per genotype. Bars represent the mean with standard deviation. P-values were calculated using repeated measures Student’s t-test (unpaired, two-tailed). **g**. Brightfield images of primary, *ex vivo* acinar cells from KC;G6pd^wt^ and KC;G6pd^mut^ mice grown in Matrigel for 0, 2, and 3 days. Cells were treated with vehicle (water) or glutathione ethyl ester (GSHee; 1 mM). Scale bars = 25μm. **h-i**. Quantification of acinar cells undergoing ADM in primary cultures at Day 2 (**h**.) and Day 3 (**i**). Grey bars are KC;G6pd^wt^ cells treated with vehicle (light) or GSHee (dark), blue bars are KC;G6pd^mut^ cells treated with vehicle (light) or GSHee (dark). Each datapoint represents one mouse. n = 3 mice per genotype. Bars represent the mean with standard deviation. P-values were calculated using ordinary one-way ANOVA with Tukey’s multiple comparisons test. **j-k**. Violin plots showing quantification of Diameter (µm) in ADM primary cultures at Day 2 (**J**.) and Day 3 (**k**). Grey plots are KC;G6pd^wt^ cells treated with vehicle (light) or GSHee (dark), blue plots are KC;G6pd^mut^ cells treated with vehicle (light) or GSHee (dark). Plots combine 3 mice per genotype. Lines inside of the plots represent the quartiles and median. P-values were calculated using ordinary one-way ANOVA with Tukey’s multiple comparisons test. Exact p-values are as follows: KC;G6pd^wt^ vs. KC;G6pd^mut^ = 1.54×10^−13^; KC;G6pd^mut^ vs. KC;G6pd^mut^+GSHee = 2.54×10^−7^; KC;G6pd^wt^+GSHee vs. KC;G6pd^mut^+GSHee = 6.96×10^−7^. **l**. Pancreas to body weight ratios from mice of the indicated genotypes after receiving control drinking water or drinking water supplemented with NAC for 6 weeks. n = 4 KC;G6pd^wt^; n = 4 KC;G6pd^wt^ + NAC; n = 6 KC;G6pd^mut^; n = 6 KC;G6pd^mut^ + NAC. Bars represent the mean with standard deviation. Ratios are not significantly different, as calculated by using ordinary one-way ANOVA with Tukey’s multiple comparisons test. **m**. Pathological grading of pancreas tissue of the indicated genotypes after receiving control drinking water or drinking water supplemented with NAC for 6 weeks. Datapoints represent the % of total tissue area with acinar cells, ADM, and PanIN lesions. n = 4 mice for each treatment. Bars represent the mean with standard deviation. Source data

We next asked whether reduced NADPH-producing enzyme function led to increased oxidative stress in the pancreas. To assess lipid peroxidation, we performed immunoblotting on pancreas lysates for hyperoxidized peroxiredoxin 3 (HO-PRDX3), malondialdehyde (MDA) and 4-hydroxynonenal (4-HNE) (Fig. 4a,b). Pancreata from KC;G6pd^mut^ and KC;Me1^flox/flox^ mice exhibited increased lipid peroxidation compared with their littermate controls (Fig. 4a and Extended Data Fig. 9a). There was trend toward increased MDA immunostaining in KC;G6pd^mut^ pancreas (compared to KC;G6pd^wt^) and significantly more MDA in KC;Me1^flox/flox^ pancreas (compared to KC;Me1^+/+^) (Extended Data Fig. 9b–d). Pancreata from KC;G6pd^mut^ mice also had higher levels of 4-HNE than KC;G6pd^wt^ controls (Extended Data Fig. 9e,f).

### Antioxidant treatment rescues accelerated ADM formation in primary acinar cultures

Given the established role of ROS in ADM formation^13^, we hypothesized that accelerated ADM could be rescued by antioxidant treatment. Acinar cells from KC;G6pd^mut^ and KC;G6pd^wt^ mice were treated with vehicle or the cell-permeable glutathione analogue glutathione ethyl ester (GSHee). Consistent with our in vivo findings, KC;G6pd^mut^ acinar cells underwent ADM more rapidly in ex vivo culture compared with KC;G6pd^wt^ cells, which was restored by GSHee treatment (Extended Data Fig. 9g–k). We performed similar experiments using acinar cells isolated from *Me1*^+/+^ and *Me1*^−/−^ mice (Supplementary Fig. 7a–c). Acinar cells were 3D-embedded in Matrigel and cultured for 3 days with TGFα to induce ADM. Cells were treated with vehicle, GSHee or *N*-acetylcysteine (NAC) antioxidants. We observed a significant increase in ADM formation in *Me1*^−/−^ cells compared to controls, which could be rescued with NAC treatment (Supplementary Fig. 7a–c). As with G6PD deficiency, the persistence of enhanced ADM in isolated *Me1*^−/−^ acinar cells indicates that this phenotype is redox regulated. Finally, we wanted to determine how ADM formation rates compared between *G6pd*-mutant, *Me1*-null and *Nrf2*-null acinar cells. In collagen cultures, both KC;G6pd^mut^ and *Me1*^−/−^ cells had significantly higher ADM formation rates than the respective controls (Supplementary Fig. 7d–g). Surprisingly, *Nrf2*^−/−^ cells failed to form ADM in both collagen cultures and Matrigel cultures (Supplementary Fig. 7f–h). Taken together, our experiments suggest that ROS induce NRF2-signalling to promote ADM.

### Antioxidant treatment rescues accelerated lesion formation in KC;G6pd^mut^ mice

To test whether antioxidants can rescue lesion formation in vivo, we provided KC;G6pd^wt^ and KC;G6pd^mut^ mice drinking water with or without NAC. No significant differences in pancreas to body weight ratios were observed between mice given control and NAC water (Extended Data Fig. 9l). H&E staining and histological grading showed that pancreas from KC;G6pd^mut^ mice had significantly more ADM and PanIN lesions, coincident with a decrease in per cent acinar area, compared to pancreas from KC;G6pd^wt^ mice (Fig. 4b–c and Extended Data Fig. 9m). NAC treatment in KC;G6pd^mut^ mice reduced lesions back to levels found in KC;G6pd^wt^ mice and increased per cent acinar area (Fig. 4b,c). This finding was also supported by a significant increase in the percentage of CK19^+^ cells in pancreas from KC;G6pd^mut^ mice compared to KC;G6pd^wt^ controls, which could be rescued by NAC treatment (Fig. 4d,e).

### Inhibiting glutathione biosynthesis promotes ADM in human acinar cells and PanIN formation in KC mice

**Extended Data Fig. 10:**
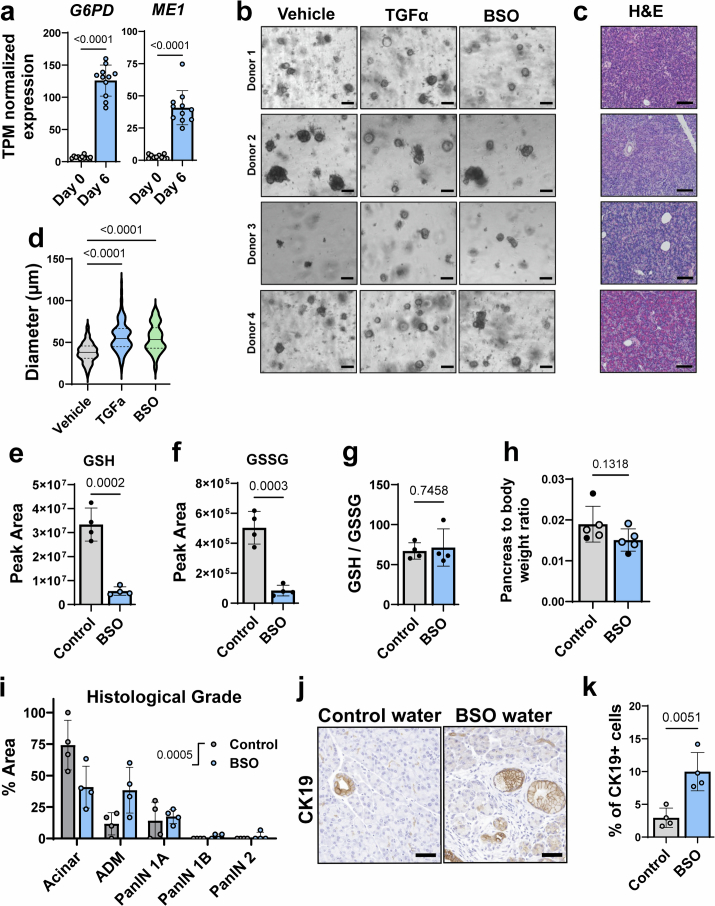
Representative cultures from donor pancreas and additional analyses in BSO-treatment experiments. **a**. Transcripts per million (TPM)-normalized expression of *G6PD* and *ME1* from RNA-sequencing of human acinar cells treated with TGFα for 6 days (n = 11). Dataset from^40^ and accessed through NCBI GEO. Bars represent the mean with standard deviation. P-values were calculated using a Student’s t-test (unpaired, two-tailed). Exact p-value for *G6PD* = 1.04×10^−8^; for *ME1* = 1.06×10^−7^. **b**. Bright field images of ex vivo acinar cell cultures isolated from human pancreata (obtained from 4 independent tissue donors) at Day 0 and Day 6. Cells were treated with Vehicle (DMSO), TGFα (50 ng/mL), or buthionine sulfoximine (BSO; 100 μM). Scale bar = 100μm. **c**. Hematoxylin & eosin (H&E)-stained human donor pancreas. Tissue was adjacent to the region selected for acinar cell isolation and culture. Scale bar = 100μm. **d**. Violin plots showing quantification of Diameter (μm) in ADM primary cultures at Day 6. Grey plots are vehicle-treated cells, blue plots are TGFα-treated cells, green plots are BSO-treated. Plots combine counts from cells isolated from all 4 donors. Lines inside of the plots represent quartiles and median. P-values were calculated using two-way ANOVA with Dunnett’s multiple comparisons test. Metabolite peak areas of GSH (**e**.) and GSSG (**f**.) from pancreas tissue as determined by mass spectrometry. **g**. Ratio of GSH to GSSG peak areas. Data in **e-g**. represent male and female KC mice that were administered control or buthionine sulfoximine (BSO; 20 mM) water for 5 weeks. n = 4 mice per treatment. Bars represent the mean with standard deviation. P-values were calculated using a Student’s t-test (unpaired, two-tailed). **h**. Pancreas to body weight ratios from mice that received control or BSO-containing drinking water for 5 weeks. Bars represent the mean with standard deviation. P-values were calculated using a Student’s t-test (unpaired, two-tailed). **i**. Pathological grading of pancreas tissue. Datapoints represent the % of total tissue area with acinar cells, ADM, and PanIN lesions. n = 4 mice for each treatment. Bars represent the mean with standard deviation. P-values were calculated using a two-way ANOVA with Tukey’s multiple comparisons test. **j**. Immunostaining for cytokeratin 19 (CK19; ductal, metaplastic, neoplastic cells) in pancreata from 5-6 week old KC mice administered control or BSO-containing water for 5 weeks. Scale bar = 50μm. **k**. Percent of CK19+ cells in the pancreas as quantified from KC mice administered control or BSO-containing water for 5 weeks. n = 4 mice for each genotype. Bars represent the mean with standard deviation. P-values were calculated using a Student’s t-test (unpaired, two-tailed). Source data

We analysed a previously published human ADM RNA-seq dataset^40^ and observed a significant upregulation of both *G6PD* and *ME1* at day 6 following TGFα treatment (Extended Data Fig. 10a). We obtained human donor pancreas through a research partnership with the Gift of Life Michigan organ and tissue donation programme^6^. Human acinar cells from four independent donors were isolated and 3D-cultured in Matrigel for 6 days (Extended Data Fig. 10b,c). To induce ADM, cells were treated with TGFα (positive control)^41^. To decrease antioxidant capacity, we depleted cellular GSH pools with buthionine sulfoximine (BSO), an inhibitor of de novo glutathione synthesis (schematic in Fig. 4f). Compared to vehicle, TGFα and BSO treatment significantly increased ADM formation (Fig. 4g,h) and significantly increased the diameter of ADM spheres (Extended Data Fig. 10d). We also saw elevated G6PD and ME1 protein expression in human ADM by immunostaining (Fig. 4i).

Based on the experiments in human cultures, we reasoned that decreasing glutathione would be sufficient to drive more metaplastic and neoplastic lesions in vivo. We provided KC mice control drinking water or water that was supplemented with BSO for 5 weeks. BSO treatment significantly reduced glutathione levels in the pancreas (Extended Data Fig. 10e–g) and did not significantly change pancreas weight to body weight ratios (Extended Data Fig. 10h). Histological grading of H&E-stained pancreas tissue showed that BSO treatment significantly increased ADM and PanIN lesions, concurrent with a decrease in acinar cell area (Fig. 4j,k and Extended Data Fig. 10i). This finding was also supported by a significant increase of CK19^+^ cells in BSO-treated KC mice (Extended Data Fig. 10j,k). These data suggest that inhibiting glutathione synthesis is sufficient to accelerate lesion formation in KC mice, overall pointing to redox regulation as a key factor in pancreatic precancer formation.

## Discussion

Metabolic dependencies in advanced cancers have been extensively studied and targeted therapeutically, but metabolic alterations in precancer are not well characterized. Previous work has demonstrated that *Kras*^G12D^ drives ROS production and antioxidant pathways during ADM and pancreatic tumourigenesis^12–14,42–44^. Yet the function of ROS in tumourigenesis is complex, with studies showing pro-tumour and anti-tumour roles. This duality stems, in part, from ROS-dependent cell toxicity versus mitogenic signalling. Our study and others show that antioxidant treatment dampens ADM formation ex vivo and tumourigenesis in vivo by lowering ROS levels^13,45^. By contrast, abrogating antioxidant defences through genetic deletion of *Nrf2*, a primary antioxidant regulator, results in high levels of ROS that drive cellular senescence and reduce PanIN formation^12^. NRF2-target genes also have dynamic roles in tumourigenesis, as seen in the lung, where *Gsr* loss impairs lung tumour initiation, while *Txnrd1* loss impairs tumour progression^17^. Our work complements these studies and shows that NRF2-target genes have distinct roles during each step in cancer initiation and progression, as demonstrated by the promotion of early lesions with G6PD deficiency or *Me1* loss, but the promotion of PDAC only with *Me1* loss.

We interrogated the role of NADPH-producing enzymes G6PD and ME1 in pancreatic precancer formation. G6PD deficiency accelerated the rate of *Kras*^G12D^-driven ADM in primary, ex vivo acinar cultures and increased the rate and number of ADM and PanIN lesions in KC mice. Similarly, *Me1* loss accelerated ADM in cultures and increased the rate and number of ADM and PanIN lesions in KC mice. Our data suggest that this acceleration is a result of increased oxidative stress from decreased NADPH production, as antioxidant treatment can rescue metaplasia and neoplasia acceleration in cultures and in vivo. Additionally, depleting antioxidant levels by glutathione synthesis inhibition promotes ADM and PanIN formation in KC mice. These findings are consistent with a recent study showing increased precancerous lesions with the loss of *Aldh1l2*, a gene that encodes a mitochondrial NADPH-producing enzyme^46^. Together, this highlights the interconnection of NADPH metabolism and how it functions to maintain cellular reducing potential as a barrier against precancer formation.

Although both G6PD deficiency and *Me1* loss promote early tissue transformation, *Me1* loss uniquely accelerates malignant progression to PDAC. This highlights clear differences in the necessity of these metabolic enzymes in precancer and advanced disease. Outside of their shared roles in NADPH production, G6PD and ME1 appear to impose distinct metabolic constraints on evolving lesions, which may only become limiting as cells have more demands in malignant progression. Our data suggest a conserved function of NADPH regulation during early tissue transformation, but NADPH-independent metabolic activities for PanIN to PDAC transformation.

Recent studies show a surprisingly high frequency of precancerous lesions present in the healthy human pancreas^6,7^. These findings raise several questions on how lesions form, which lesions progress to cancer and what pathways underlie formation and progression. Changes in cell and systemic metabolism during lesion development and progression pose attractive targets for biomarkers and dietary interventions. Indeed, new research suggests that circulating formate levels may serve as a biomarker for PDAC progression^46^. Furthermore, this supports a role of oxidative stress in regulating cancer initiation in the pancreas^12–14,18,29^. However, a careful and comprehensive understanding of precancer metabolic changes, especially in antioxidant pathways, is required before translation to humans. As a cautionary example, clinical trials of antioxidant supplementation in individuals who were at high risk for lung cancer increased cancer incidence and mortality^47,48^. Therefore, additional studies in precancer across tissues and in diverse patient populations are necessary to improve human health outcomes.

## Methods

### Animal use

Mouse experiments were conducted under the guidelines of the Office of Laboratory Animal Welfare and approved by the Institutional Animal Care and Use Committees at the University of Michigan under protocol PRO00010606. Animal experiments complied with all relevant ethical regulations. Mice were housed in ventilated racks in a pathogen-free animal facility with a 12 h light, 12 h dark cycle, 30–70% humidity and 20–23 °C temperatures maintained in the animal facility at Rogel Cancer, University of Michigan. Mice were overseen by the Unit for Laboratory Animal Medicine. Mice were fed ad libitum (5L0D, PicoLab Laboratory Rodent Diet). Both male and female mice were used in experiments unless specifically indicated. Tumour monitoring was performed to ensure that the maximal tumour size and burden was not exceeded. In line with ethical guidelines, a tumour diameter of 2 cm in any single dimension required that the mice be killed. All mice in this study were killed at the experimental endpoint, and the maximum tumour size was never exceeded.

### Mouse strains

LSL*-Kras*^G12D^ mice (Jackson Laboratory, strain no. 008179) were crossed with *Ptf1a*^CreERTM^ mice^49^ to generate LSL-*Kras*^G12D^; *Ptf1a*^CreERTM^ mice and maintained on a C57BL/6J background. G6PD-deficient mice^32,33,50^ were originally generated in the C3H strain. They have been crossed to other strains, including C57BL/6J. G6PD-deficient mice were maintained on a mixed background and compared to age-matched littermate controls. LSL-*Kras*^G12D^; *Ptf1a*^Cre^; *G6pd*^mut^ or *G6pd*^wt^ mice were maintained on a mixed background and were confirmed *Nnt* wild-type by genotyping. LSL-*Kras*^G12D^; *Ptf1a*^Cre^ mice^51^ were crossed to *Me1*^flox/flox^ mice^35^ to generate LSL*-Kras*^G12D^; *Ptf1a*^Cre/+^; *Me1*^flox/flox^ mice and maintained on a C57BL/6J background. All the LSL-*Kras*^G12D^; *Ptf1a*^Cre^; *Me1*^flox/flox^ mice presented in these studies were *Nnt*-null. LSL-*Kras*^G12D^; *Ptf1a*^Cre^; *Me1*^flox/flox^ mice in these studies were compared to LSL-*Kras*^G12D^; *Ptf1a*^Cre^; *Me1*^+/+^ littermates and LSL-*Kras*^G12D^; *Ptf1a*^Cre^ age-matched, historic controls. Germline, whole-body *Me1-*null mice (*Me1*^−/−^)^35^ were maintained on a C57BL/6J background and compared to wild-type littermates for experiments. *Nrf2*^−/−^ mice^52^ (*Nfe2l2*^*tm1Ywk*^; Jackson Laboratory, strain no. 017009) were maintained on a C57BL/6J background.

### Genotyping

Genotyping was performed through a commercial vendor (Transnetyx) or in house. Genotyping performed by Transnetyx used real-time PCR with specific probes designed for each target. In-house genotyping was performed using DNA extracted from tail or ear tissue using DirectPCR Lysis Reagent (Viagen Biotech). Genotyping primer sequences are included in Supplementary Table 1. Thermal cycling was performed using GoTaq Green Master Mix (Promega). Gel electrophoresis was performed on 2% agarose gel with 10,000X SYBR Safe DNA gel stain (Invitrogen). DNA Ladders (1 kb and 100 bp) (New England Biolabs) were used to measure amplicon size. Gels were run at 140 V for 50 min and imaged using the ChemiDoc Imaging System (Bio-Rad). G6pd mouse PCR primers were made between exon 1 and intron 1, around the reported mutation site. The PCR product was digested using the DdeI restriction enzyme and run on a 3% agarose gel. *G6pd* wild-type mice have two bands (214 and 55 bp), *G6pd*-mutant homozygotes have one band (269 bp) and *G6pd*-mutant heterozygotes have three bands (269, 214 and 55 bp).

### Treatment procedures in mice

#### NAC

NAC (*N*-acetyl-L-cysteine; Cayman Chemical, 20261) was added to drinking water at a concentration of 30 mM with pH adjusted to 7, matching control water. Water was changed weekly. Male and female LSL-*Kras*^G12D^; *Ptf1a*^Cre^; *G6pd*^mut^ or *G6pd*^wt^ mice aged 5–6 weeks were randomly selected to receive NAC water or normal water for 5–6 weeks.

#### BSO

BSO (L-Buthionine-(S,R)­-sulfoximine; Cayman Chemical, 14484) was added to drinking water at a concentration of 20 mM^53^ and changed weekly. Male and female LSL-*Kras*^G12D^; *Ptf1a*^CreERTM^ mice aged 5–6 weeks were randomly selected to receive BSO water or normal water for 5 weeks.

#### Cerulein

Serum and pancreas from cerulein-treated wild-type mice (C57BL/6J) were used as positive controls for serum amylase tests and anti-CPA1 western blots, respectively. Two male and two female mice were administered 75 µg kg^−1^ cerulein (dissolved in sterile PBS) by intraperitoneal injection once every hour for 8 h, then administered two additional hourly injections the following day before blood and tissue collection.

### Acinar cell isolation from mice

Pancreata from 8–10 week-old mice were dissected and rinsed twice in 5 ml cold Hanks’ Balanced Salt Solution (HBSS; Gibco). Tissue was minced with sterile scissors into 1–5 mm-sized pieces, then centrifuged for 2 min at 300*g* and 4 °C. Media was aspirated, and minced tissue was digested with 5 mg of Collagenase P (Roche, Sigma, 11213857001) dissolved in 5 ml cold HBSS. Tissue was digested at 37 °C for 15–20 min with gentle agitation on an orbital shaker. Collagenase P was inhibited by the addition of 5 ml cold 5% FBS (Corning, 35-010-CV) in HBSS. Cells were centrifuged for 2 min at 300*g* and 4 °C, then washed with 5 ml cold 5% FBS in HBSS. This process was repeated twice. Cells were passed through a 500 μm strainer (Pluriselect, Fisher, NC0822591), followed by a 100 μm strainer (Fisherbrand, 22363549) and then pelleted through 10 ml of 30% FBS gradient. Cells were resuspended in media and incubated at 37 °C for at least 4 h before plating. Cells were cultured in 1× Waymouth’s media (Gibco, 11220035) supplemented with 0.4 mg ml^−1^ soybean trypsin inhibitor (Gibco, 17075-029), 1 μg ml^−1^ dexamethasone (Sigma-Aldrich, D4902) and 0.5% gentamicin (Lonza, 17-519L). All media was sterilized through a 0.22 μm PVDF membrane (Millipore, Stericup Filter Unit, SCGVU01RE; Steriflip Filter Unit, SE1M179M6).

### 3D acinar cell culture

For measuring transdifferentiation rates, acinar cells were embedded in Matrigel (Corning, 354234) domes or Collagen gel (Gibco, A1048301; as described in previous work^54^) and plated in 48-well plates. Culture media was added on top of the solidified matrix and changed on day 2 after plating. Cells were treated with vehicle (water), 1 mM GSHee (Cayman Chemical, 14953) or 500 µM NAC (Cayman Chemical, 20261; pH adjusted to 7) dissolved in water. Cells were imaged using the EVOS FL Auto Imaging System (ThermoFisher Scientific). Isolated acinar cells were seeded in triplicate, and per cent ADM quantifications were counted from 4× optical fields per well. The diameter of ADM spheres was measured using the line tool in ImageJ (v.1.51n) from imported 4× images. Statistics for per cent ADM and diameter were calculated using GraphPad Prism 10 and analysed using ordinary one-way ANOVA with Dunnett’s multiple comparisons test.

For *Kras*^G12D^ induction in acinar cell cultures, acinar cells were isolated from three independent male LSL-*Kras*^G12D^; *Ptf1a*^CreERTM^ littermates at 11 weeks old. Cells were treated with either vehicle (ethanol) or 2 μM 4-OHT (Cayman Chemical, 19519) to induce expression of mutant *Kras*^G12D^. RNA and metabolites were isolated 24 h after plating vehicle-treated acinar cells (vehicle) and 24, 48 and 72 h after plating 4-OHT-treated cells (days 1, 2 and 3, respectively).

### RNA isolation and RNA-seq from acinar cell cultures

Acinar cells were grown free-floating in non-tissue culture-treated six-well plates for RNA isolation. Primary acinar cell cultures were collected by centrifugation for 2 min at 300*g*. Half of the cell pellet was lysed in RLT buffer containing 1% β-mercaptoethanol (Sigma-Aldrich, M6250), flash-frozen in liquid nitrogen and stored at −80 °C for brief storage. Samples were quickly thawed and passed through a Qiashredder column (Qiagen, 79654). RNA was purified using a RNeasy Plus Mini Kit (Qiagen, 74136) and analysed on a Nanodrop 2000c (Thermo Scientific) spectrophotometer for quantification. RNA was further assessed with a Qubit Assay to determine concentration and quality assessment with an Agilent TapeStation through the University of Michigan Advanced Genomics Core. All samples for sequencing had an RNA integrity number greater than 7.9 and a DV200 greater than 90. Samples were submitted to GENEWIZ from Azenta Life Sciences for standard RNA-seq using Illumina NovaSeq X Plus with 20 million reads per sample and ribosomal RNA removal using PolyA selection for messenger RNA (mRNA) species. ERCC RNA Spike-In Mix (ThermoFisher Scientific, 4456740) was added to normalized total RNA before library preparation using the manufacturer’s protocol. RNA-seq libraries were prepared using the NEBNext Ultra II RNA Library Prep Kit for Illumina following the manufacturer’s instructions (NEB). In brief, mRNAs were initially enriched with Oligod(T) beads. Enriched mRNAs were fragmented for 15 min at 94 °C. cDNA fragments were end-repaired and adenylated at 3′ ends, and universal adaptors were ligated to cDNA fragments, followed by index addition and library enrichment by PCR with limited cycles. The sequencing library was validated on the Agilent TapeStation (Agilent Technologies) and quantified by a Qubit 3.0 Fluorometer (Invitrogen) and qPCR (KAPA Biosystems). Data are publicly available from NCBI’s Gene Expression Omnibus (GEO) database (GSE313699).

### RNA-seq data analysis

RNA-seq fastq reads were transferred to the University of Michigan Great Lakes computational cluster for pseudoalignment to the mouse genome (Gencode version M35) using Salmon (v.1.9.0)^55^ to generate a counts per sample matrix. RNA-seq count matrices were analysed in edgeR to identify differentially expressed genes between experimental conditions. The expression data were filtered to remove genes with low counts using the edgeR filterByExpr() function with default settings. Normalization factors and dispersion parameters were then calculated before generating a log_2_-transformed counts per million matrix for analysis. Overall gene expression patterns per sample were first visualized with a multidimensional scaling plot. Differential gene expression between each timepoint (days 1, 2 and 3) and vehicle control was estimated using the quasi-likelihood negative binomial generalized log-linear approach in edgeR. Genes were considered differentially expressed between a treatment and control at a false discovery rate-adjusted *P* value of <0.05. Enriched differentially expressed genes per treatment were identified using gene set enrichment analysis in FGSEA^56^. To identify enriched transcription factor binding, the top 500 overexpressed or underexpressed genes within a condition were uploaded to the Enrichr web tool^26–28^ to quantify enrichment for ENCODE and ChEA consensus transcription factor targets. We visualized gene expression patterns using pheatmap() in R or using GraphPad Prism. Analyses were conducted using R software (v.4.2.2).

### ^14^C glucose incorporation into CO_2_

Cells were treated with 1 μCi 1-^14^C (Perkin Elmer, NEC043X050UC) or 6-^14^C glucose (Perkin Elmer, NEC045X050UC) and incubated at 37 °C for 4 h. To release ^14^CO_2_, 150 µl of 3 M perchloric acid (Sigma-Aldrich, 244252) was added to each well and immediately covered with Whatman paper saturated with phenylethylamine (Sigma-Aldrich, P6513) and then incubated at room temperature (25 °C) overnight. The Whatman paper was then analysed by scintillation counting (Beckman, LS6500) and normalized to surrogate protein quantification.

### Real-time qPCR

RNA was extracted from acinar cell cultures using the RNeasy Mini Kit (Qiagen, 74104) according to the manufacturer’s instructions. RQ1 RNase-Free DNase (Promega, M6101) was added to samples, and then cDNA was generated using iScript Reverse Transcription Supermix (Bio-Rad, 1708840). Quantitative PCR was performed using Fast SYBR Green Master Mix (Applied Biosystems, 4385612) on a QuantStudio 5 PCR System (Applied Biosystems). Gene expression levels were normalized to the expression of the endogenous control *Mrpl*. Relative expression was calculated using the 2^−ΔΔCT^ method^57^. Experiments were performed in technical triplicates. Primer sequences are included in Supplementary Table 2.

### Sample processing for histology

Mice were killed by CO_2_ asphyxiation or isoflurane overdose, followed by cervical dislocation. Tissue was quickly dissected and fixed overnight at room temperature with zinc formalin fixative (Z-Fix; Anatech). Fixed tissues were transferred to 70% ethanol and embedded in paraffin. Primary acinar cells cultured in Matrigel were rinsed in cold PBS, pelleted and fixed in 10% formalin at 4 °C for 1 h. Cells were rinsed in PBS, moved to 70% ethanol, pelleted, mounted in 2% agar (Fisher, BP1423) and embedded in paraffin. Samples were processed using a Leica ASP300S Tissue Processor (Leica Microsystems) and cut into 5 μm-thick sections.

### Immunohistochemistry

Tissue sections were deparaffinized with Histo-Clear (National Diagnostics) and rehydrated with graded ethanol and water. Samples were quenched with 1.5% hydrogen peroxide in 100% methanol for 15 min at room temperature. Antigen retrieval was performed in sodium citrate buffer (2.94 g sodium citrate, 500 μl Tween 20, pH 6.0) using a pressure cooker on high or by maintaining slides at a rolling boil for 20 min. Slides were blocked in PBS containing 2.5% BSA (Sigma-Aldrich) and 0.2% Triton X-100 for 45 min to 1 h at room temperature. The slides were then incubated overnight at 4 °C in primary antibodies, with the exception of the anti-malondialdehyde primary antibody, which was incubated for 1 h at room temperature. After rinsing with PBS, slides were incubated with secondary antibodies for 1 h at room temperature. Blocking of endogenous biotin, biotin receptors and avidin binding sites was performed using the Avidin/Biotin Blocking Kit (Vector Laboratories) per the manufacturer’s protocol. Substrate reaction and detection was performed using DAB Peroxidase (HRP) Substrate Kit (With Nickel), 3,3′-diaminobenzidine (Vector Laboratories, SK4100) as detailed per the manufacturer’s protocol. Slides were counterstained, mounted in Permount Mounting Medium (Fisher Scientific), coverslipped and allowed to dry overnight before imaging. Antibody information is included in Supplementary Table 3.

### Counterstains and special stains

H&E staining was performed using Mayer’s haematoxylin solution and Eosin Y (Thermo Fisher Scientific). Blue and Nuclear Fast Red staining was performed using the Alcian Blue (pH 2.5) Stain Kit (Vector Laboratories, H-3501) per the manufacturer’s protocol.

### Imaging and quantification of immunostaining

Imaging was performed automatically using a whole-slide scanner (Vectra Polaris, Akoya Biosciences) or manually using an Olympus BX53F microscope. Whole-slide scanning was performed by the University of Michigan Tissue and Molecular Pathology Shared Resource. To quantify staining from slide scans, three to five regions of interest at ×10 magnification were randomly selected using QuPath^58^ (v.0.6.0) and the positive DAB signal was quantified. Manual imaging was performed on an Olympus BX53F microscope fitted with an Olympus DP80 digital camera (Olympus Life Science), using CellSens Standard software. To quantify staining from manual images, three to five images were taken per slide at ×10 magnification. Images were anonymized and randomized, and a positive DAB signal was quantified using QuPath^58^ (v.0.6.0). For tissue grading, at least three ×20 fields were reviewed by a pathologist and graded in a blinded manner.

### Polar metabolite isolation

#### From primary ex vivo acinar cell cultures

Floating cells from primary cultures were collected by centrifugation for 2 min at 300*g*. Half of the cell pellet was used to isolate intracellular metabolites by lysis in dry ice-cold 80% methanol with repeated vortexing for 10 min. Media from cultures was used to isolate extracellular metabolites by adding 0.2 ml of media to 0.8 ml dry ice-cold 100% methanol. The solution was repeatedly vortexed and incubated on dry ice for 10 min. Both intracellular and extracellular samples were centrifuged at 15,000*g* for 10 min at 4 °C to pellet insoluble material. The metabolite supernatant was transferred to a fresh tube and dried by vacuum centrifugation. The dried pellet was resuspended in a 50:50 methanol:water mixture for metabolomics.

#### From pancreas tissue

Upon collection, pancreas tissue was flash-frozen in liquid nitrogen and stored at −80 °C until metabolite isolation. Frozen tissue samples were weighed and added to 2 ml Eppendorf tubes with 1 ml of ice-cold 80% methanol (diluted in 20% ultrapure water). Then, samples were immediately disrupted on a Qiagen TissueLyser II with metallic beads (Qiagen, 69989) at 30 s intervals until fully homogenized. Samples were centrifuged at 15,000*g* for 10 min at 4 °C to pellet insoluble material. Metabolite supernatant volume was normalized by pancreas tissue weight, transferred to a fresh tube and dried by nitrogen purging or vacuum centrifugation. The dried pellet was resuspended in a 50:50 methanol:water mixture. To detect reduced glutathione in samples, pancreas tissue was dissociated in ice-cold 80% methanol (diluted in 20% ultrapure water) containing 25 mM *N*-ethylmaleimide (Cayman Chemical, 19938, CAS 128-53-0). After homogenization, samples were incubated at room temperature for 30 min, allowing for derivatization of thiols. The remainder of sample preparation followed the same procedure described above.

### Metabolomics methods

General LC–MS-grade chemicals and solutions used included acetonitrile (Fisher, A955-4), methanol (Sigma-Aldrich, A456-4), isopropanol (Fisher, A461-4), ammonia acetate (Sigma-Aldrich, 73594-25G-F), ammonia solution (Sigma-Aldrich, 5438300100) and formic acid (Fisher, A117-50). The water was purified to 18 MΩ·cm using a Millipore filtration system.

#### Targeted metabolomics from primary acinar cell cultures

Steady state LC–MS/MS-based targeted metabolomics was performed according to published methods^59,60^ and described here. Samples were run on an Agilent 1290 Infinity II LC system coupled to a 6470 Triple Quadrupole (QqQ) mass spectrometer. The LC–MS/MS platform comprised a 1290 Infinity II LC Flexible Pump (Quaternary Pump), a 1290 Infinity II Multisampler, a 1290 Infinity II Multicolumn Thermostat with six-port valves and the 6470 triple quad mass spectrometer. Compound optimization, calibration and data acquisition were performed using the Agilent MassHunter Workstation Software LC–MS Data Acquisition for 6400 Series Triple Quadrupole MS (software version 10). A total of 145 metabolites were detected in the samples from a 242-metabolite reference library.

The QqQ data were pre-processed with Agilent MassHunter Workstation QqQ Quantitative Analysis Software (B0700). Raw metabolite abundance levels from day 1, day 2 and day 3 samples were divided by the median of raw metabolite abundance levels in vehicle samples to allow for metabolite comparison across days, dependent on their change compared to vehicle. Values were then log_2_ transformed for visualization in volcano plots and heatmaps. Median normalized values were analysed by a two-tailed *t*-test to determine statistical significance, with a *P* < 0.1 cutoff to indicate metabolites of interest. Heatmaps and volcano plots were generated using GraphPad Prism 10. Principal component analysis plots were generated using MetaboAnalyst (https://www.metaboanalyst.ca). Metabolomics source data for this experiment are included in Supplementary Tables 4–6 (intracellular metabolites) and Supplementary Tables 7–9 (extracellular metabolites).

#### NADP^+^ and NADPH detection in pancreas tissue

Samples were analysed on an Agilent 1290 Infinity II LC system coupled to an Agilent 6470 triple quadrupole (QqQ) mass spectrometer, according to previously published methods^59,60^ and described here. An additional method for the quantification of NAD, NADH, NADP and NADPH was performed on the same hardware with the following parameters.

Solvent A comprised 97% water and 3% methanol, with 15 mM acetic acid and 10 mM tributylamine at a pH of 5. Solvent C consisted of 15 mM acetic acid and 10 mM tributylamine in methanol. Solvent D (acetonitrile) was used as the wash solvent. The LC system seal wash consisted of 90% water and 10% isopropanol, and the needle wash solvent was 75% methanol and 25% water. Tributylamine (GC-grade, 99%; ACROS Organics), acetic acid (LC–MS-grade Optima; Fisher Chemical), InfinityLab Deactivator additive and ESI–L Low Concentration Tuning mix (Agilent Technologies) and LC–MS-grade water, acetonitrile, methanol (Millipore) and isopropanol (Fisher Chemical) were used.

An Agilent ZORBAX RRHD Eclipse Plus C18 column (2.1 × 50 mm, 1.8 µm particle size) was used. The gradient profile was 90% solvent A, 10% solvent B for 0–0.8 min; 80% solvent A, 20% solvent B for 0.8–2.5 min; 1% solvent A, 99% solvent B for 2.5–6.5 min; 1% solvent A, 99% solvent B for 6.5–7.5 min; 90% solvent A, 10% solvent B for 7.5–7.6 min; and 90% solvent A, 10% solvent B for 7.6–8.5 min. The flow rate was set at 0.5 ml min^−1^ with an injection volume of 3 µl at a temperature of 35 °C.

Analytical standards were used to optimize compounds, and a dynamic multiple reaction monitoring (dMRM) strategy was used. Samples were also run alongside standards for NADPH (Cayman Chemical, 9000743, CAS 2646-71-1) and NADP^+^ (Cayman Chemical, 10004675, CAS 698999-85-8) dissolved in 50% methanol. Data were pre-processed in Agilent MassHunter Quantitative Analysis Software (B0700). Metabolomics source data for this experiment are included in Supplementary Table 10.

#### Glutathione detection in pancreas tissue

The Thermo Scientific Orbitrap IQ-X LC–MS system was used to detect glutathione. The system consisted of two Vanquish Horizon UHPLC binary pumps, a dual Vanquish autosampler, two Vanquish column compartments with a switch valve for a two-column setup configuration, a Thermo Multiwavelength Detector (CG) and an Orbitrap IQ-X high-resolution mass spectrometer. Data acquisition, instrument tuning, calibration and optimization were performed using the Thermo Scientific Xcalibur data system (v.4.6.67.17) on a Dell Opex-EX3 computer running Windows 10 Professional.

For targeted and untargeted applications, the Waters Acquity UPLC BEH amide column (2.1 × 100 mm, 1.7 µm particle size) with a Waters UPLC BEH Amide VanGuard pre-column (1.7 µm) was used for high-throughput acquisition. Solvent A consisted of 10 mM ammonium acetate in water with 10 mM ammonia (pH 8.0). Solvent B consisted of acetonitrile. The pump seal wash and autosampler wash comprised 50% isopropanol and 50% water with 0.1% formic acid, and the needle wash solvent consisted of 80% acetonitrile and 20% water. The LC profile was a 0.2 ml per minute flow rate of of 85% B for 0–0.5 min, 15% B for 0.5–15 min, 15% B for 15–17 min, 85% B for 17.1 min and 85% B for 22 min. The column compartment temperature was maintained at 30 °C, and the autosampler was held at 5 °C; the injection volume was 3 µl.

The Thermo Scientific Orbitrap IQ-X mass spectrometer was calibrated weekly using Pierce FlexMix Calibration Solution (A39329), delivered by infusion into the micro-ESI probe at 5 µl per minute, with 2 ppm accuracy for all calibrations. Source parameters included H-ESI positive static voltage 4,000 V; negative static voltage, 3,500 V; sheath gas, 40 arbitrary units (a.u.); auxiliary gas, 5 a.u.; sweep gas, 1 a.u.; ion transfer tube temperature, 300 °C and vapourizer temperature, 350 °C. Orbitrap resolution was 60 K, and the mass scanning range was 50–1,000 Da. The data type was centroid, using polarity-switching data acquisition mode.

Data processing was performed by Skyline (v.12.1) and Thermo Compound Discoverer (v.3.4).

MS/MS analysis was performed using the Thermo AcqX acquisition method, applying the same LC method on both the MS1 scan and MS/MS, with collision energies of 20, 30 and 50 eV in positive and negative modes separately. One blank, one scan and five MS/MS injections were used from the pooled quality control samples. All MS1 and MS2 datasets were combined to build a data workflow, using Compound Discoverer (v.3.4) for the final output.

Metabolomics source data for this experiment are included in Supplementary Table 11.

### Donor sample procurement and processing

The process of acquiring donor pancreas tissue for research purposes through the Gift of Life Michigan has been previously published^6^ and is described in brief here. Donor pancreata that were ineligible for transplant or for which there were no eligible recipients were collected at the Gift of Life Michigan Donor Care Center and transported to the University of Michigan. Written consent from family members for the use of tissues for research was obtained by the Gift of Life Michigan. Upon arrival, the pancreas was dissected into head, body and tail regions by a pancreatobiliary surgeon. Tissue was used for primary, ex vivo acinar cultures. An adjacent region was fixed overnight in Z-fix (Anatech), rinsed with PBS and 70% ethanol and then paraffin-embedded for histology. Acquisition and use of donor pancreas for research purposes was approved by the Gift of Life Michigan research review group and the University of Michigan Institutional Review Board (HUM00025339). We obtained informed consent, and the studies complied with all relevant ethical regulations.

### Primary acinar cell culture from donor pancreas

Donor mouse pancreata were prepared for primary, ex vivo acinar culture using a similar procedure as described above. In brief, tissue was minced with sterile scissors in cold HBSS (Gibco) into 1–5 mm-sized pieces, centrifuged, rinsed and then digested with Collagenase P (Roche, Sigma, 11213857001) dissolved in HBSS. Tissue was digested at 37 °C for 20–30 min with gentle agitation on an orbital shaker. HBSS containing 5% FBS (Corning, 35-010-CV) was added, and cells were centrifuged and rinsed with 5 ml cold 5% FBS in HBSS three times. Cells were passed through a 500 μm strainer (Pluriselect, Fisher, NC0822591), then through a 100 μm strainer (Fisherbrand, 22363549) and then pelleted through a 30% FBS gradient. Cells were resuspended in 1× Waymouth’s media (Gibco, 11220035) supplemented with 0.4 mg ml^−1^ soybean trypsin inhibitor (Gibco, 17075-029), 1 μg ml^−1^ dexamethasone (Sigma-Aldrich, D4902) and 0.5% gentamicin (Lonza, 17-519 L). Cells were cultured at 37 °C overnight in a non-tissue culture-treated six-well plate. The following day, floating cells were pelleted, embedded in Matrigel (Corning, 354234) and plated in 24-well plates for measuring ADM transdifferentiation rates. Culture media was added on top of the solidified matrix and changed on days 1, 3 and 5 after plating. Cells were with vehicle (PBS), 50 ng ml^−1^ TGFα (R&D Systems, 239A100) or BSO (Cayman Chemical, 14484; dissolved in PBS). Cells were imaged daily using the EVOS FL Auto Imaging System (ThermoFisher Scientific). Isolated acinar cells were seeded in triplicates, and per cent ADM quantifications were counted from 4× optical fields per well. The diameter of ADM spheres was measured using the line tool in ImageJ (v.1.51n) from imported 4× images.

### Protein detection by western blot

Pancreas tissue was flash-frozen in liquid nitrogen upon collection and stored at −80 °C. Frozen pancreas tissue was added to RIPA buffer (Sigma-Aldrich, R0278) containing protease (Roche, 04693132001) and phosphatase (Sigma-Aldrich, P5726) inhibitors. Then, samples were immediately homogenized on a Retsch TissueLyser II (129251128) with metallic beads at intervals of 30 s. Samples were centrifuged at 4 °C, and the supernatant was collected to measure protein concentration using the Pierce BCA Protein Assay Kit (ThermoFisher, 23227), according to the manufacturer’s protocol. Equal amounts of protein (10–20 µg) were subjected to separation at 135 V for 85 min on SDS–PAGE and then transferred to a methanol-activated PVDF membrane at 20 V for 60 min. Membranes were blocked with 5% milk in TBST (Tris-buffered saline containing 0.1% Tween 20) followed by incubation with primary antibodies diluted in 5% milk at 4 °C overnight. Following incubation, the membranes were washed three times with TBST and incubated with the species-appropriate secondary antibody conjugated to horseradish peroxidase for 1 h at room temperature. Membranes were then washed three times for 5 min each with TBST. Chemiluminescence was detected using Clarity ECL substrate (Bio-Rad, 170-5060), and the signal was captured with a Bio-Rad ChemiDoc Imaging System (Image Lab Touch Software v.2.4.0.03). Densitometric analysis of western blot bands was carried out using ImageJ (v.1.51n). The resulting values were normalized to the intensity of the loading control from the same membrane. Additional proteins of interest were probed on the same membrane after incubation in stripping buffer (ThermoFisher, 46430) and re-blocking.

### Glucose tolerance tests

Female mice aged 12–15 weeks were fasted for 15 h, and fasting blood glucose was measured using a Contour Next EZ Glucometer (Ascensia Diabetes Care, 90007941) with Contour Next Test strips (Ascensia Diabetes Care, 85701231). Afterwards, animals were given an intraperitoneal injection of 1.5 g kg^−1^ glucose (Sigma-Aldrich, G7021-100G) in sterile saline solution (Braun, R5201-01). Blood glucose was measured 30, 60, 90 and 120 min after injection by tail vein bleeding. Animals were restrained for less than 1 min each time blood was collected. Basal blood glucose measurements were taken from the same mice 10 days after glucose tolerance tests were performed.

### Serum amylase activity

Blood was collected from 16–18-week-old mice, and serum was separated using BD Diagnostic Solutions Serum Separator Tubes (Fisher Scientific, 02-675-185). Serum was frozen and stored until testing. Quantitative kinetic determination of α-amylase activity in serum was measured using Pointe Scientific Amylase Reagent (Fisher 23-666-111) following the manufacturer’s instructions. In brief, 10 μl of serum was added to 400 μl of reagent and read on a plate reader at 409 nm three times over 2 min. To calculate amylase activity in U l^−1^ , the average absorbance per minute was multiplied by the assay volume, then by 1,000. This product was divided by the millimolar absorptivity of 2-chloro-*p*-nitrophenol times the sample volume times the light path.

### Statistics and reproducibility

Statistics were calculated using GraphPad Prism 10. A two-tailed Student’s *t*-test was used when comparing two groups; an unpaired test was used for independent groups and a paired test was performed for matched or repeated measurements. An ordinary one-way ANOVA was used to compare the means of groups with more than 2 single-variable and unpaired or unmatched measurements. A repeated measures one-way ANOVA was used to compare the means of more than 2 groups with matched or paired measurements. A two-way ANOVA was used to compare groups with more than two variables. Tukey’s multiple comparisons test was performed to compare every mean with every other mean. Dunnett’s multiple comparison test was performed to compare every mean to a control mean. Data distribution was assumed to be normal, but this was not formally tested. Survival analysis was performed using the log-rank (Mantel–Cox) test. No statistical methods were used to pre-determine sample sizes, but our sample sizes are similar to those reported in previous publications^12–14^. Sample sizes were selected based on standard experimental group sizes to achieve acceptable power, taking into account the increased variability of animal models (three to four replicates for ex vivo experiments; four to 12 replicates for in vivo experiments). No animals were excluded from analyses. No datapoints were excluded from analyses, except in Extended Data Fig. 10d, in which two outlier datapoints were removed. Outliers were identified by analysing the raw dataset in GraphPad Prism using the ROUT (Robust Regression and Outlier Removal) method, with *Q* = 1%. Experiments were repeated at least once and results were replicated, except for the RNA-seq and metabolomics experiments, which were performed once with independent biological replicates. Samples from animals were collected on several different days, from different litters and handled by multiple investigators. Staining was performed by at least two independent investigators and replicated on separate days. RNA-seq and metabolomics experiments were performed once using samples prepared from three biological replicates. Where applicable, the sample size (*n*) of biological replicates and technical replicates is indicated in the figure legends. Animals with the relevant genotypes were randomly placed into groups to collect tissue at each timepoint, with representation from approximately equal numbers of each sex. Staining, analysis and quantification were performed on samples from each group at random. For staining from mouse tissues, an investigator imaged samples and the genotypes were known. The images were then de-identified and randomized, and the positive signal was quantified using QuPath software by a separate investigator in a blinded manner. For tissue grading, slides were de-identified, imaged and graded by a pathologist in a blinded manner.

### Availability of biological materials

All unique materials used will be promptly available from companies/suppliers or from the authors upon reasonable request, without undue qualifications. Antibodies can be purchased from corresponding suppliers (Developmental Studies Hybridoma Bank, Abcam, Sigma-Aldrich and Vector Laboratories). Mouse strains can be purchased from Jackson Laboratories (where applicable) or provided by the authors.

### Reporting summary

Further information on research design is available in the Nature Portfolio Reporting Summary linked to this article.

## Supplementary information

Supplementary Information Supplementary Figs. 1–7; Source Data Blot for Supplementary Fig. 5j

Reporting Summary

Supplementary Tables 1–3 Supplementary Tables 1–3 referenced in the Methods section

Supplementary Tables 4–11 Metabolomics datasets

Supplementary Data 1–7 Statistical Source Data for Supplementary Figs. 1–7

## Source data

Source Data Fig. 1 Statistical Source Data

Source Data Fig. 2 Statistical Source Data

Source Data Fig. 3 Statistical Source Data

Source Data Fig. 4 Statistical Source Data

Source Data Fig. 4 Unprocessed western blots

Source Data Extended Data Fig./Table 1 Statistical Source Data

Source Data Extended Data Fig./Table 2 Statistical Source Data

Source Data Extended Data Fig./Table 3 Statistical Source Data

Source Data Extended Data Fig./Table 4 Statistical Source Data

Source Data Extended Data Fig./Table 5 Statistical Source Data

Source Data Extended Data Fig./Table 6 Statistical Source Data

Source Data Extended Data Fig./Table 7 Statistical Source Data

Source Data Extended Data Fig./Table 8 Statistical Source Data

Source Data Extended Data Fig./Table 9 Statistical Source Data

Source Data Extended Data Fig./Table 10 Statistical Source Data

## Data Availability

RNA-seq data from this study, referenced in Fig. 1, are publicly available from NCBI’s Gene Expression Omnibus (GEO) database (GSE313699). RNA-seq data referenced in Extended Data Fig. 6 are publicly available from the University of Michigan’s Deep Blue Data Repository (10.7302/nd65-zg69)^31^. Human ADM RNA-seq data referenced in Extended Data Fig. 10 were obtained from GEO (GSE179248)^40^. All metabolomics datasets are available in Supplementary Tables 4–11. Source data are provided with this paper.
