# Supplementary material for: NADPH-producing enzymes restrict the formation of pancreatic precancerous lesions
Source: Nat Metab. 2026 Apr 1;8(4):824–39. doi: 10.1038/s42255-026-01496-x (PMC13121030; doi:10.1038/s42255-026-01496-x)
Supplement: Supplementary file 1 [file 42255_2026_1496_MOESM10_ESM.pdf]

Chemiluminescence

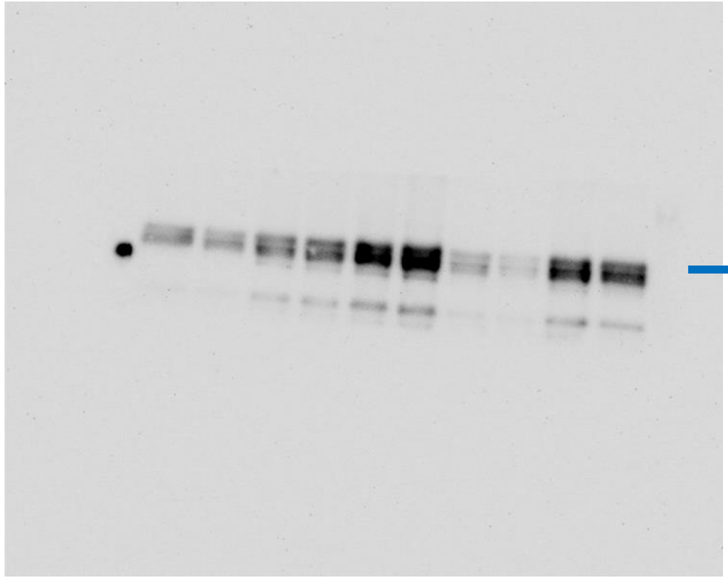

Composite

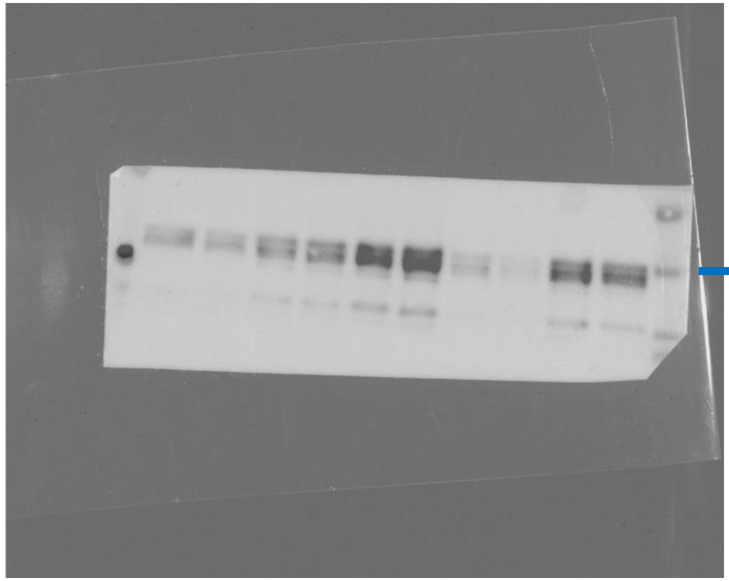

Colorimetric

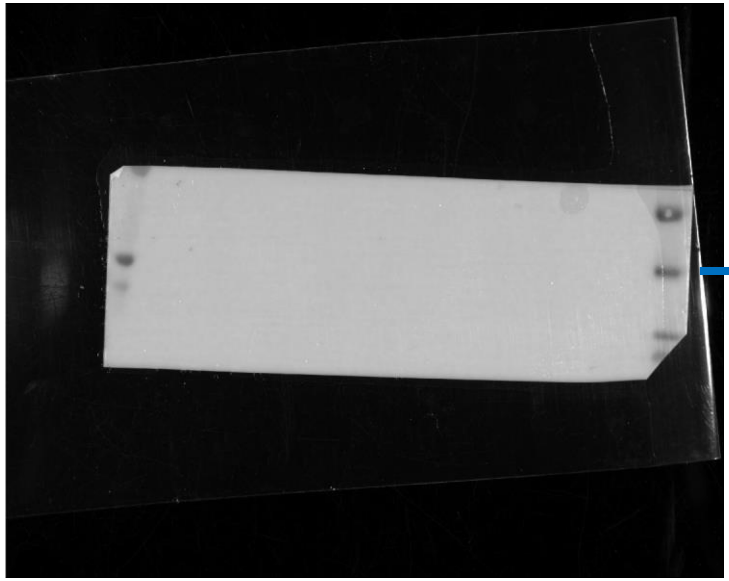

Figure 4a HO-PRDX3

Left ladder = Kaleidoscope Prestained Protein Standards, BioRad, Cat No. 1610375
Right ladder = SeeBlue™ Plus2 Pre-stained Protein Standard, ThermoFisher, Cat No. LC5925

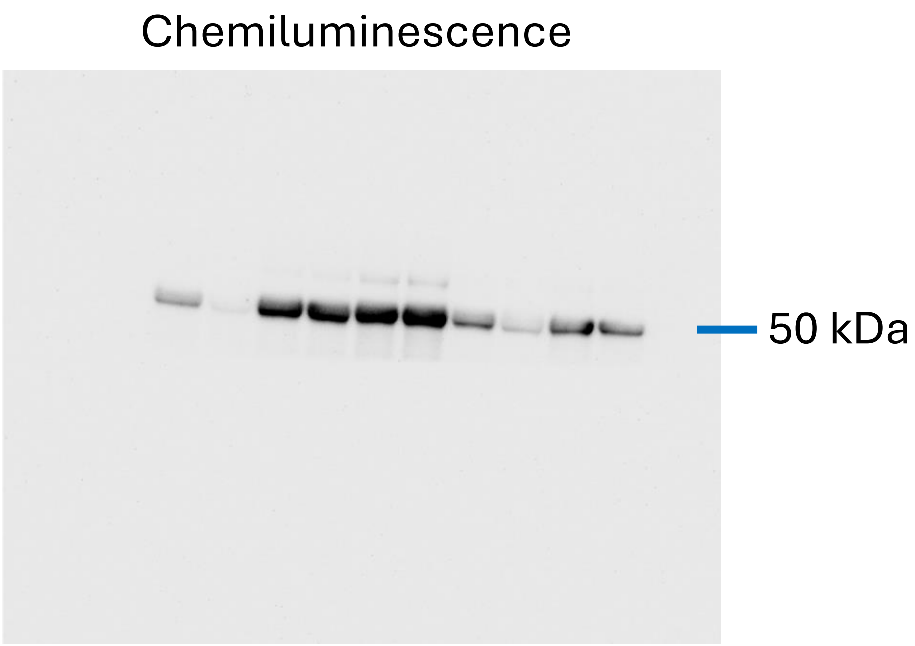

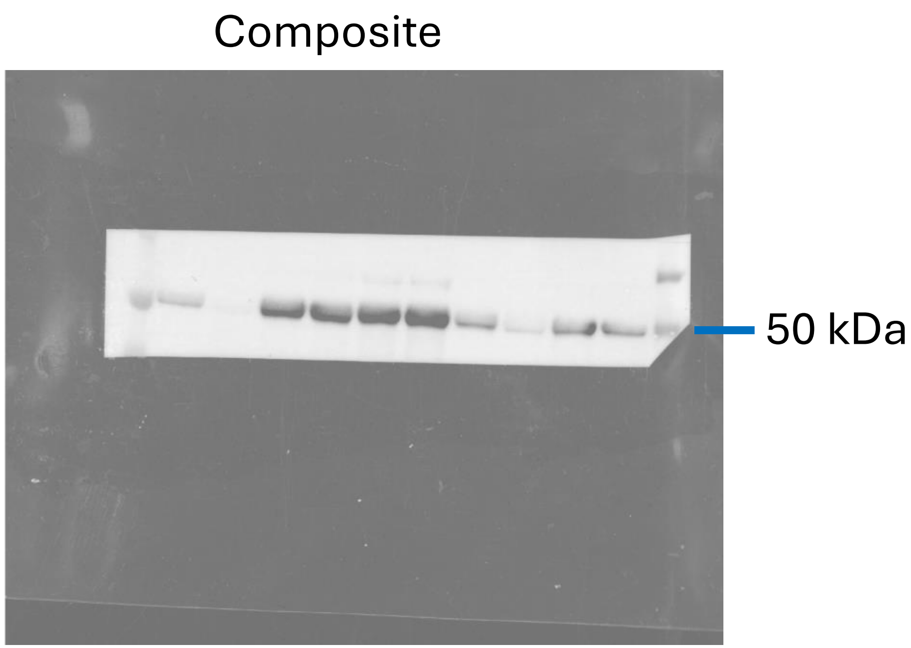

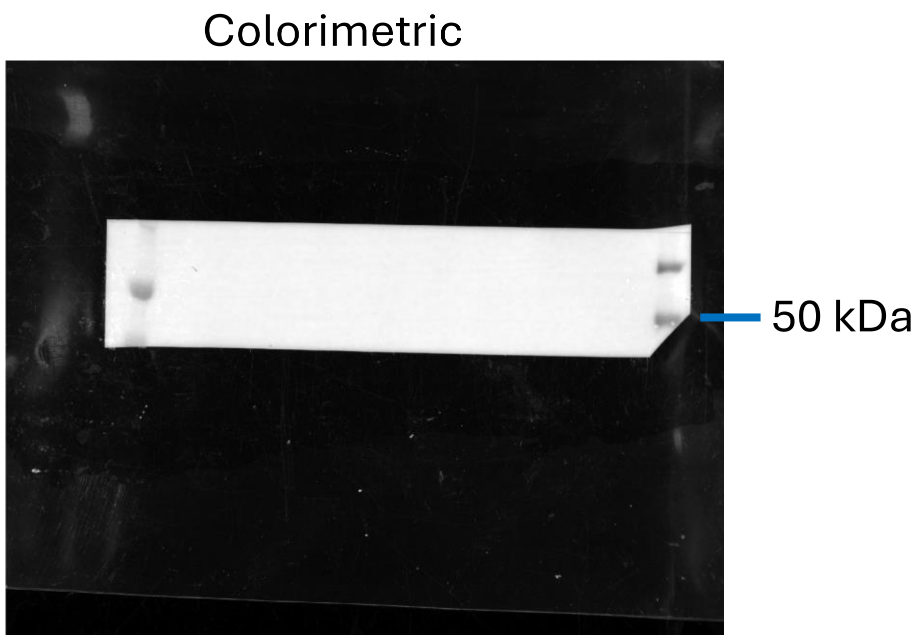

Figure 4a MDA

Left ladder = Kaleidoscope Prestained Protein Standards, BioRad, Cat No. 1610375
Right ladder = SeeBlue™ Plus2 Pre-stained Protein Standard, ThermoFisher, Cat No. LC5925

Chemiluminescence

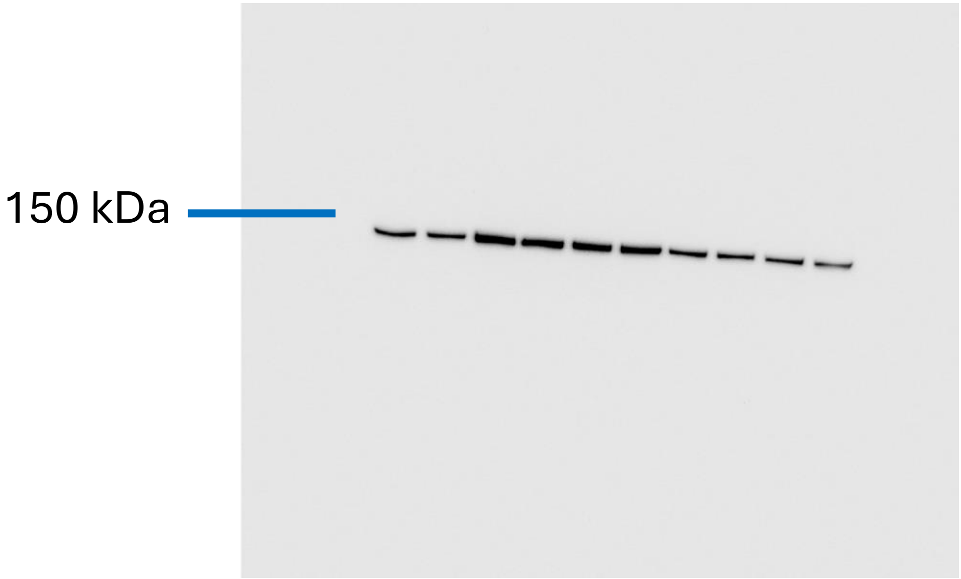

Composite

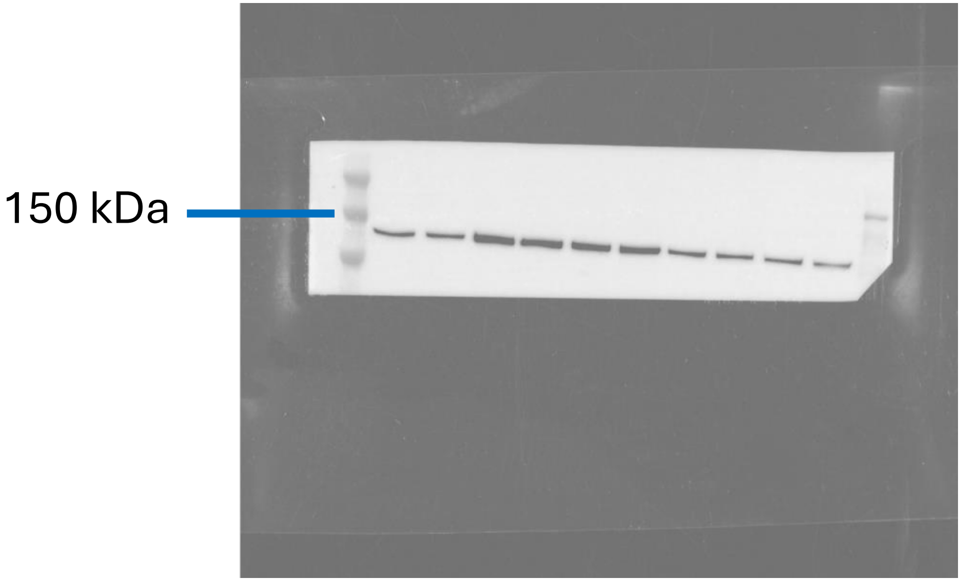

Colorimetric

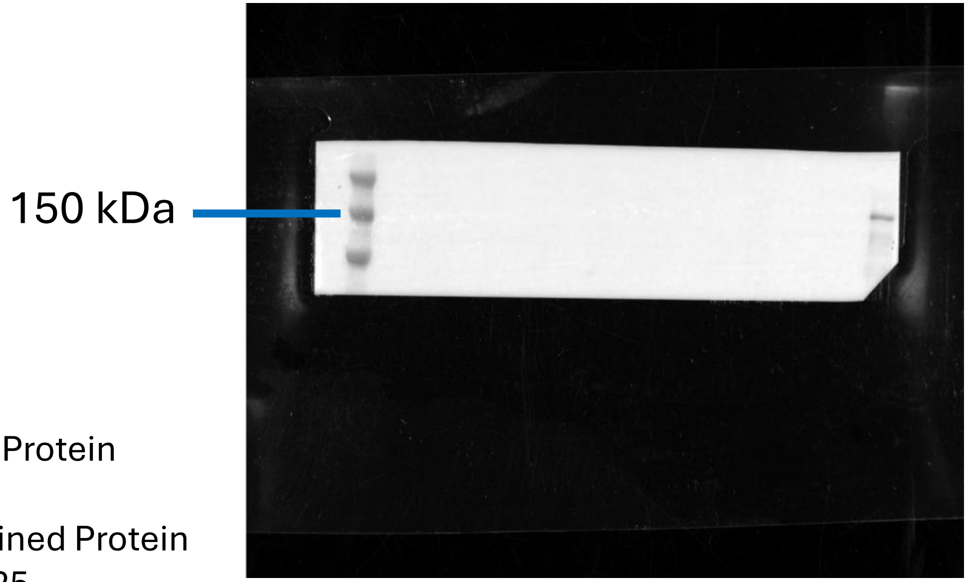

Figure 4a Vinculin

(for MDA, HO-PRDX3)

Left ladder = Kaleidoscope Prestained Protein Standards, BioRad, Cat No. 1610375
Right ladder = SeeBlue™ Plus2 Pre-stained Protein Standard, ThermoFisher, Cat No. LC5925

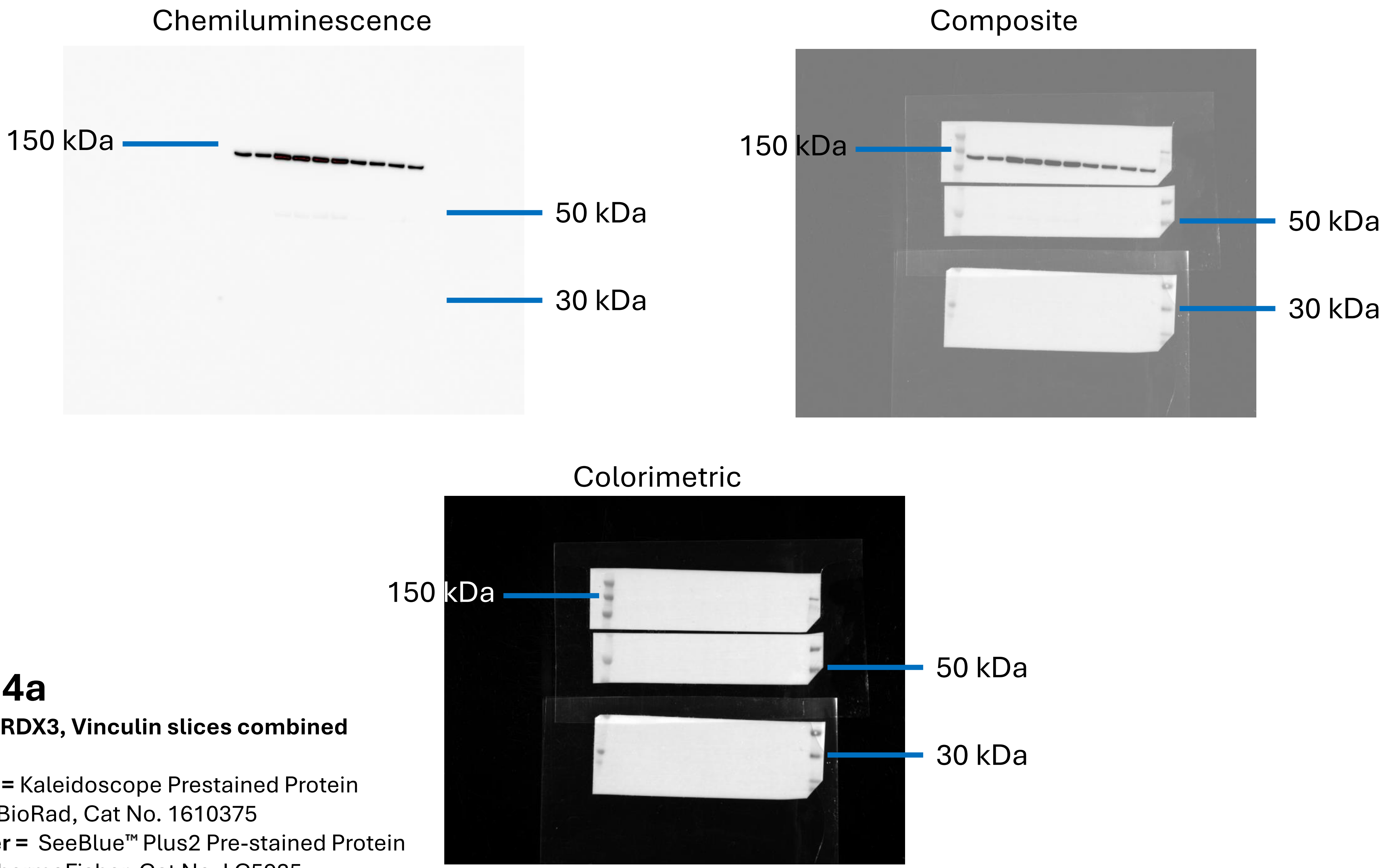

Figure 4a

MDA, HO-PRDX3, Vinculin slices combined

Left ladder = Kaleidoscope Prestained Protein Standards, BioRad, Cat No. 1610375
Right ladder = SeeBlue™ Plus2 Pre-stained Protein Standard, ThermoFisher, Cat No. LC5925

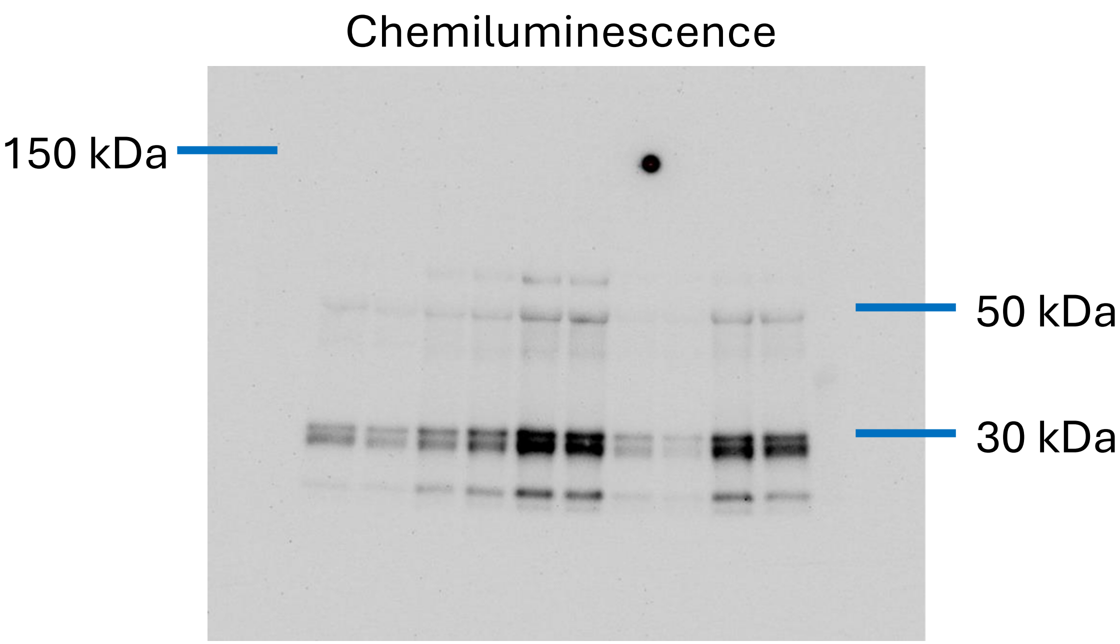

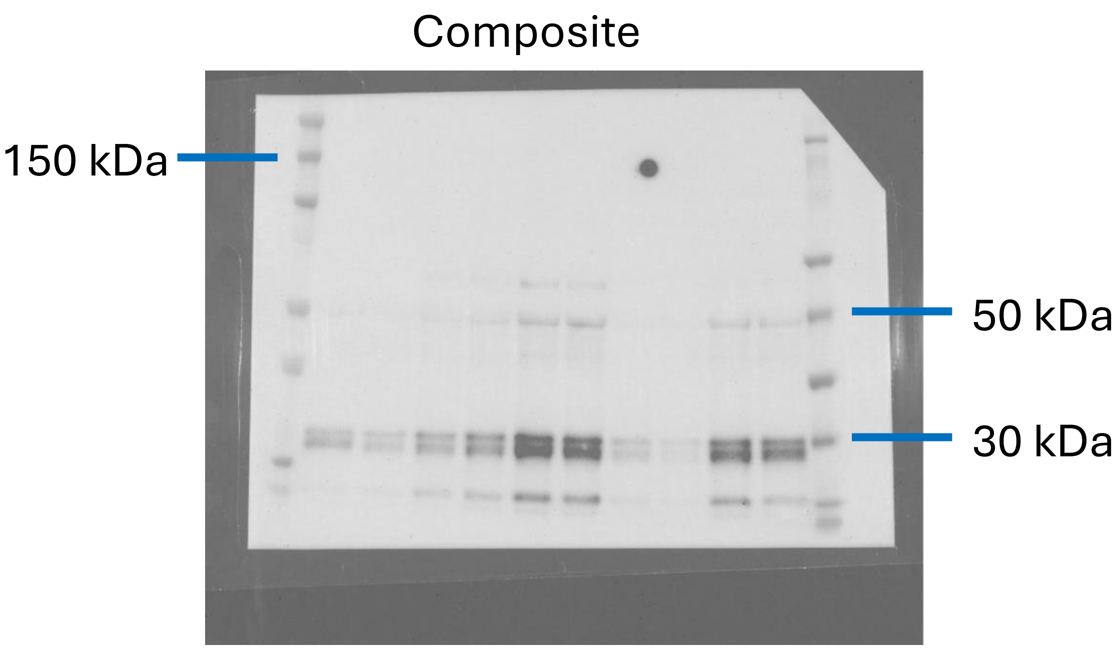

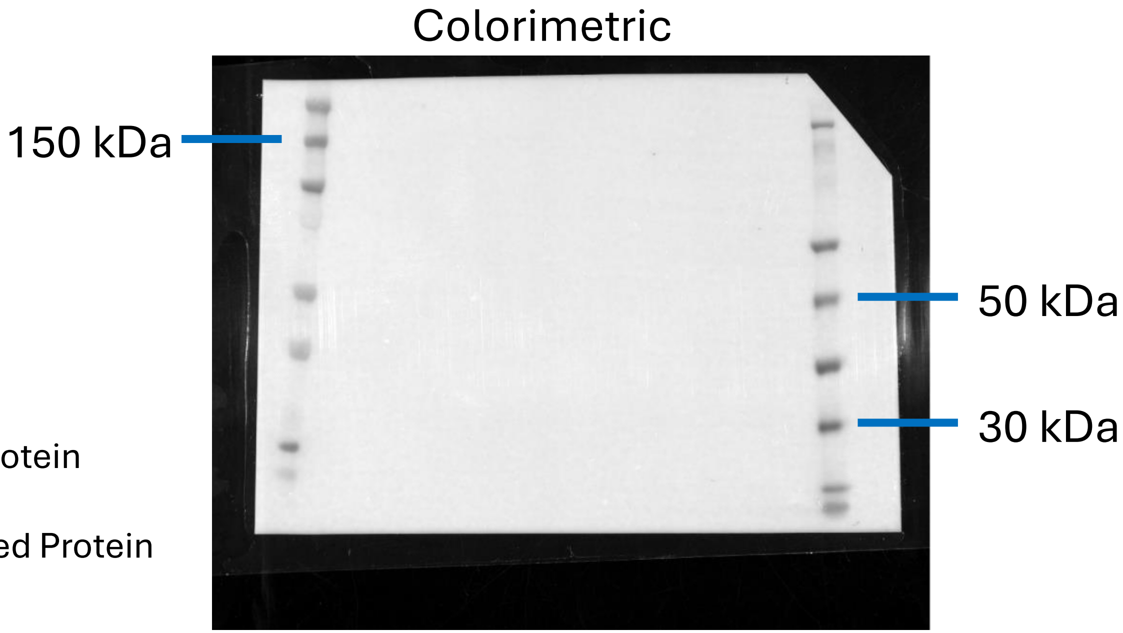

Figure 4a 4-HNE

Left ladder = Kaleidoscope Prestained Protein Standards, BioRad, Cat No. 1610375
Right ladder = SeeBlue™ Plus2 Pre-stained Protein Standard, ThermoFisher, Cat No. LC5925

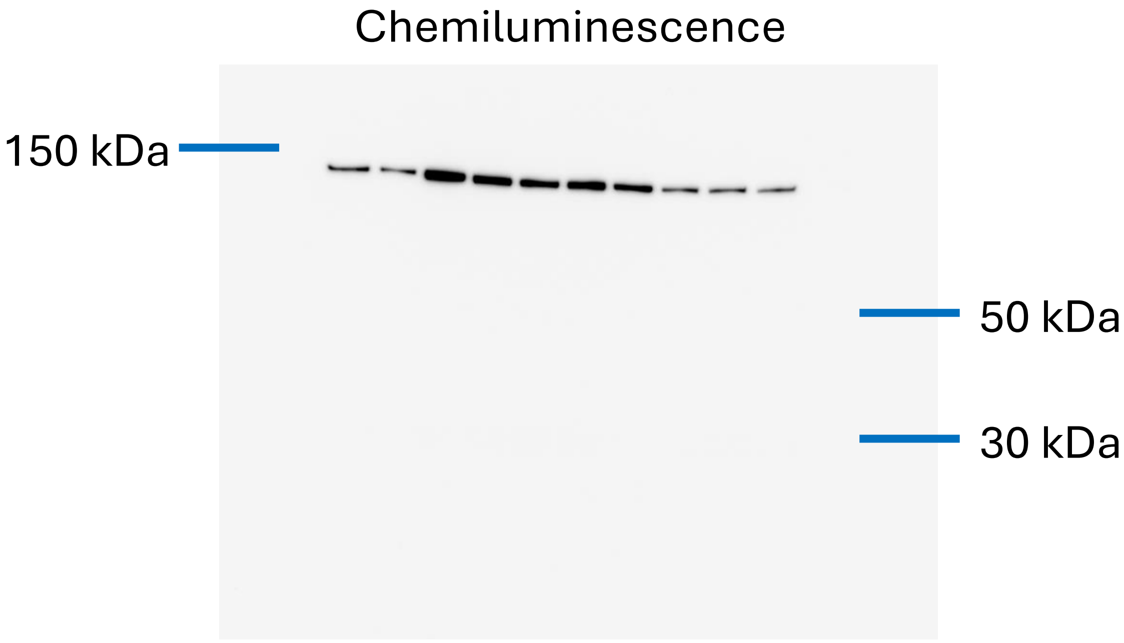

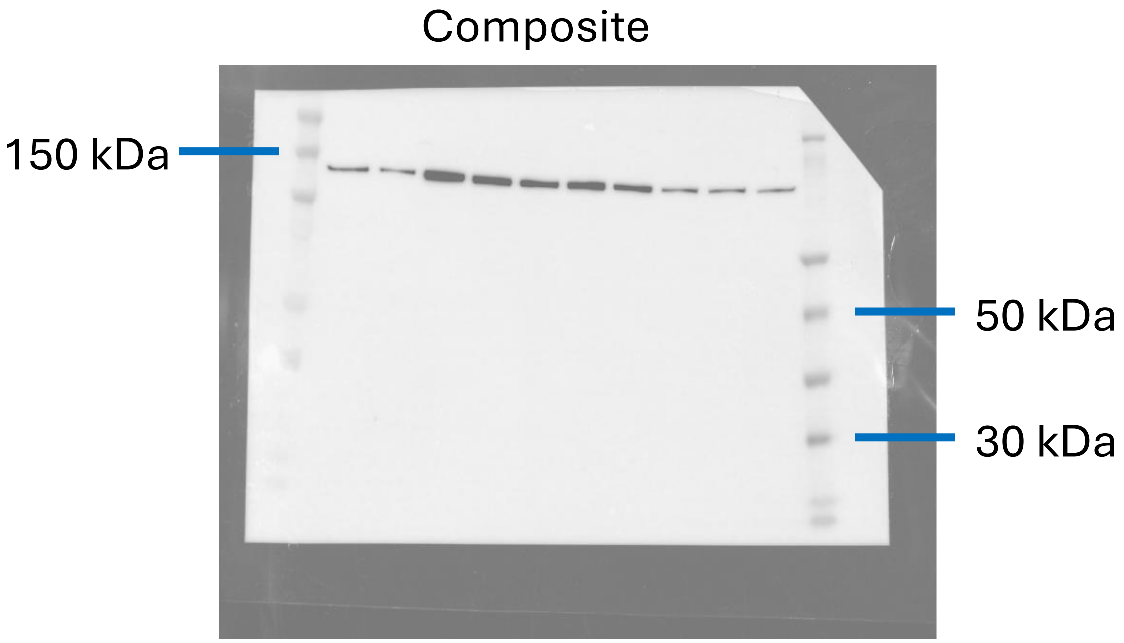

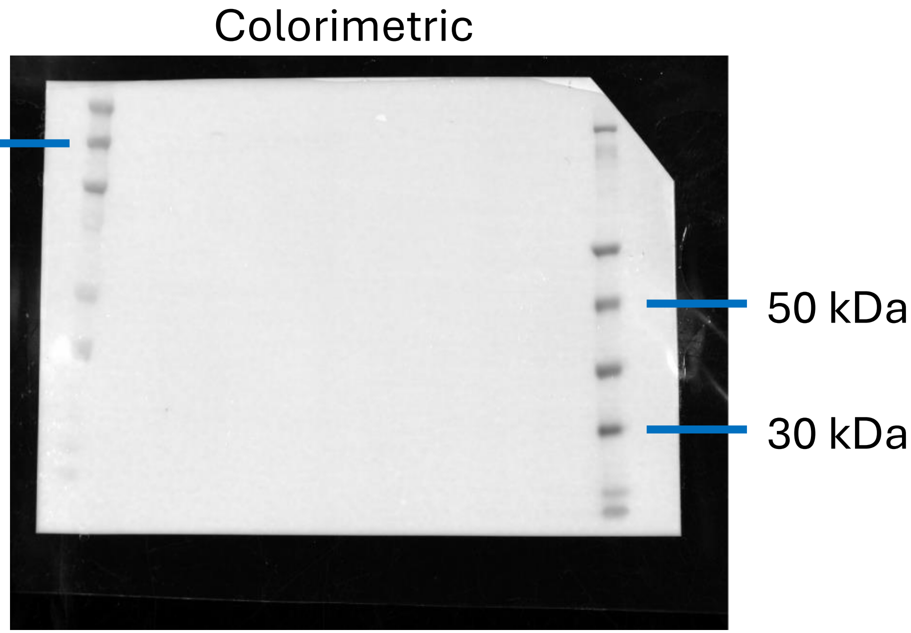

Figure 4a Vinculin

(for 4-HNE)

Left ladder = Kaleidoscope Prestained Protein Standards, BioRad, Cat No. 1610375

Right ladder = SeeBlue™ Plus2 Pre-stained Protein Standard, ThermoFisher, Cat No. LC5925
