# Supplementary material for: NADPH-producing enzymes restrict the formation of pancreatic precancerous lesions
Source: Nat Metab. 2026 Apr 1;8(4):824–39. doi: 10.1038/s42255-026-01496-x (PMC13121030; doi:10.1038/s42255-026-01496-x)
Supplement: Supplementary file 2 [file 42255_2026_1496_MOESM1_ESM.pdf]

nature metabolism

Supplementary information https://doi.org/10.1038/s42255-026-01496-x

NADPH-producing enzymes restrict the formation of pancreatic precancerous lesions

In the format provided by the authors and unedited

Contents

Supplementary Figure 1 2
Supplementary Figure 2 3
Supplementary Figure 3 4
Supplementary Figure 4 5
Supplementary Figure 5 7
Supplementary Figure 6 8
Supplementary Figure 7 9
Uncropped/unprocessed blots from Supplementary Figure 5j 10

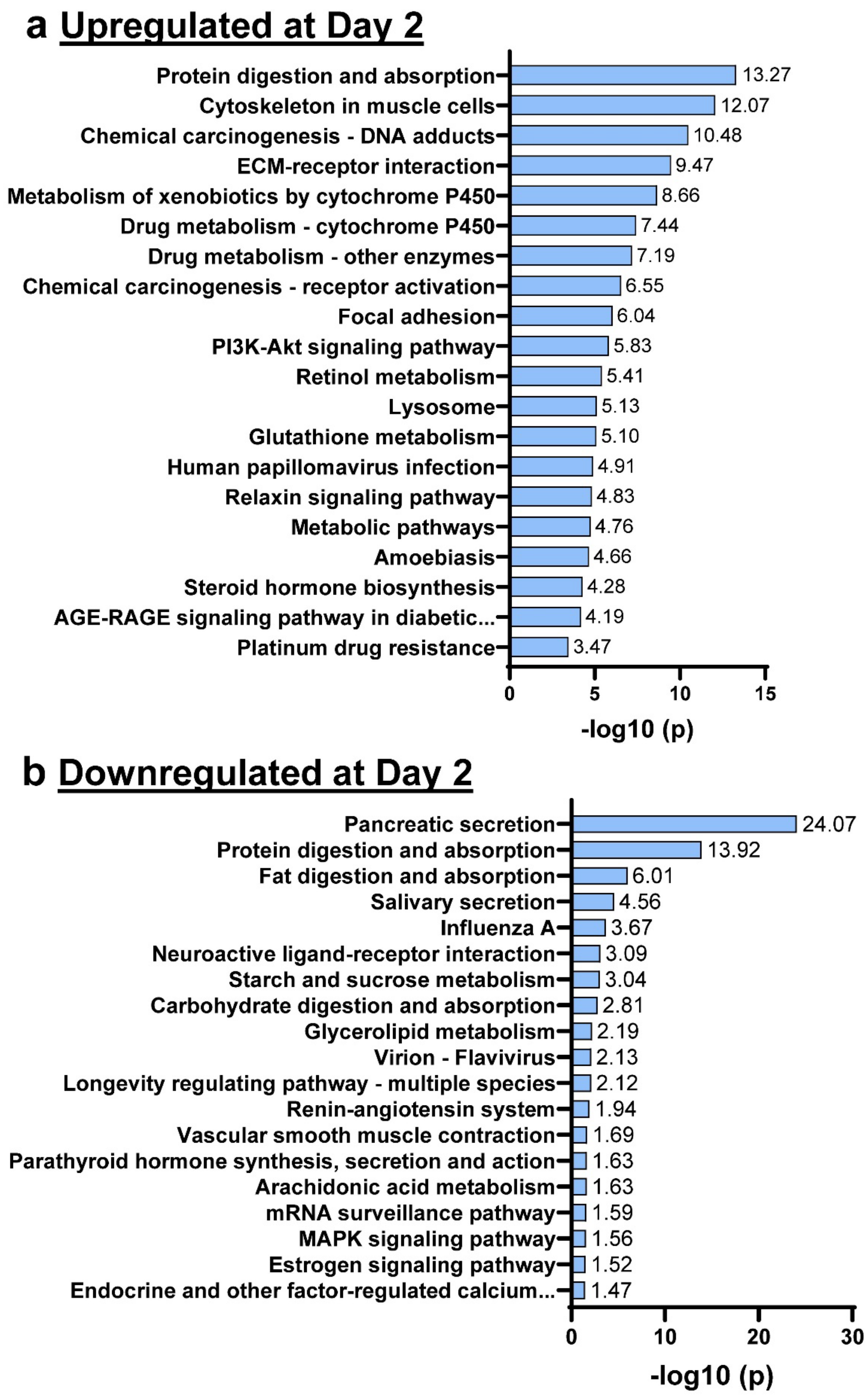

Supplementary Figure 1: Significant KEGG Pathways at Day 2
a. The top 20 significantly upregulated pathways at Day 2 identified by gene set enrichment analysis from KEGG pathways. **b.** The top 20 significantly downregulated pathways at Day 2 identified by gene set enrichment analysis from KEGG pathways.

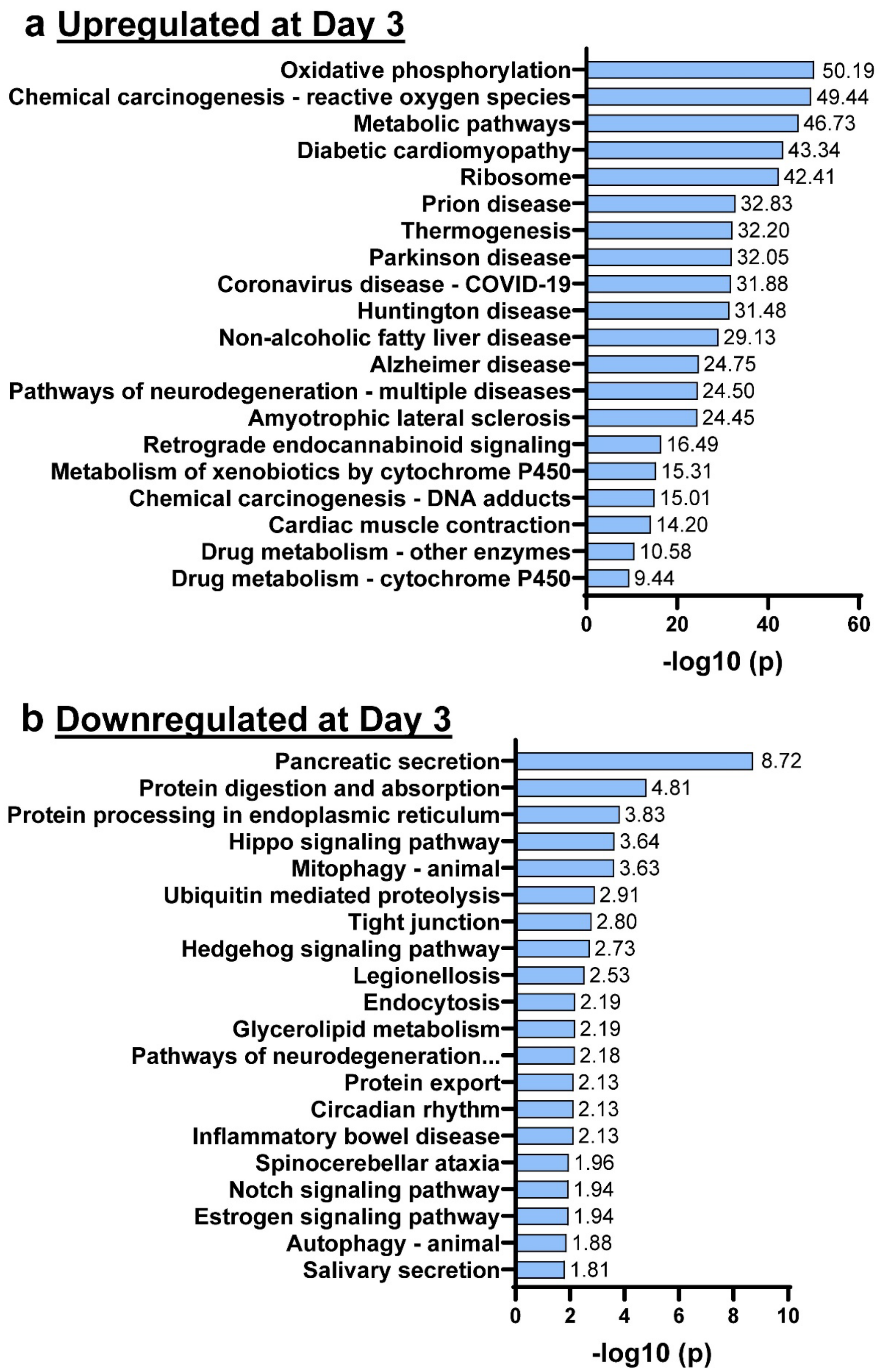

Supplementary Figure 2: Significant KEGG Pathways at Day 3
a. The top 20 significantly upregulated pathways at Day 3 identified by gene set enrichment analysis from KEGG pathways. **b.** The top 20 significantly downregulated pathways at Day 3 identified by gene set enrichment analysis from KEGG pathways.

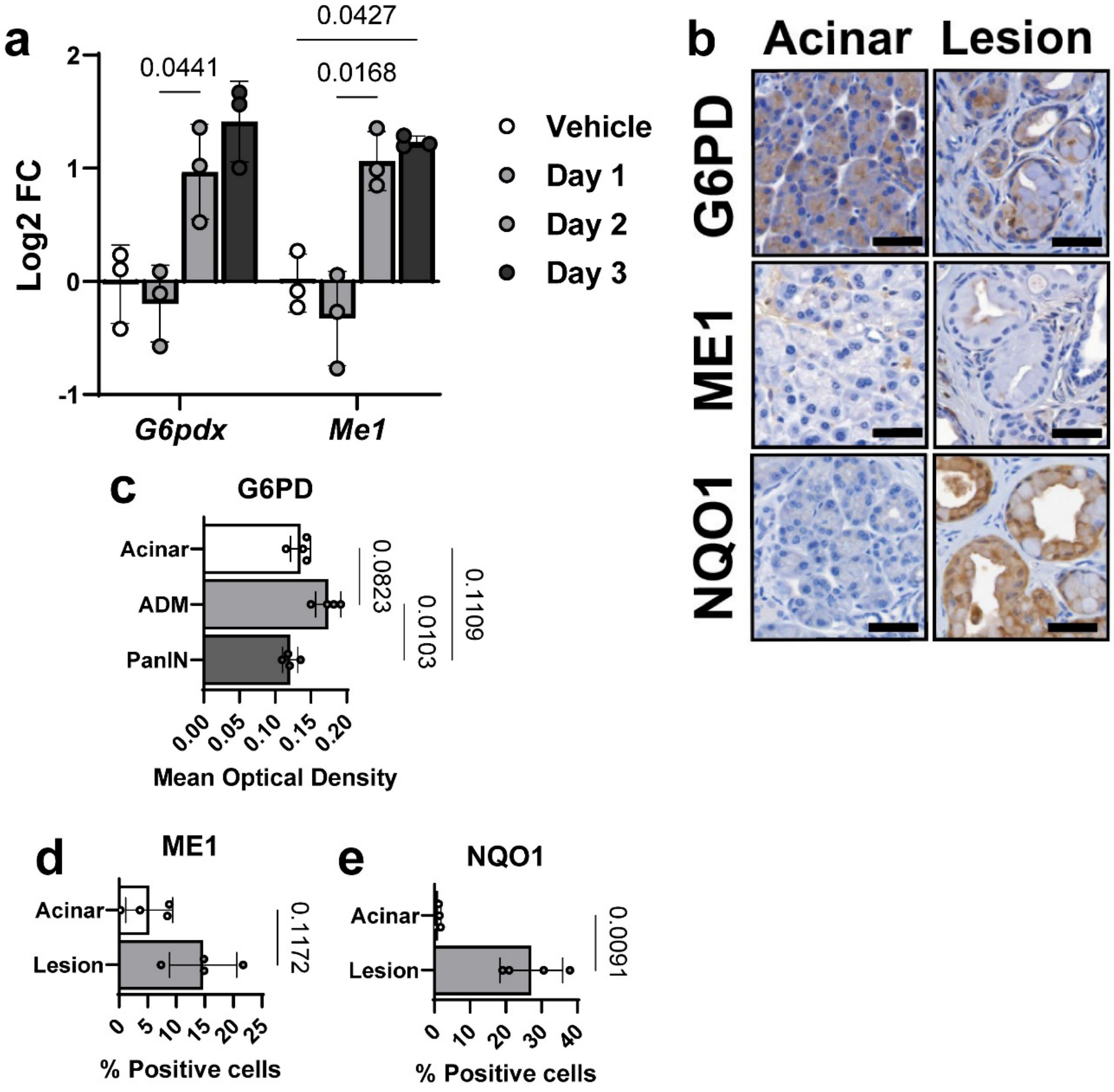

Supplementary Figure 3: Additional analysis of target genes in ADM
a. Individual datapoints representing log2FC of *G6pdx* and *Me1* gene expression from ADM cultures from each mouse after vehicle treatment or 4-OHT treatment for 3 days. n = 3 biological replicates per timepoint. Bars represent the mean with standard deviation. P-values were calculated using two-way ANOVA with Tukey's multiple comparisons tests per for each gene. **B.** Immunostaining of G6PD, ME1, and NQO1 (an NRF2-target) in 16-week-old KC mouse pancreas. Scale bar = 50µm. **C.** Quantification of mean optical density from G6PD immunostaining in KC mouse pancreas separated by expression in acinar, ADM, or PanIN regions of the tissue. n = 3 mice. Bars represent the mean with standard deviation. P-values were calculated using repeated measures one-way ANOVA with Tukey's multiple comparisons test. **D.** Quantification of percent ME1+ immunostaining in KC mouse pancreas separated by expression in acinar and lesion regions of the tissue. n = 3 mice. Bars represent the mean with standard deviation. P-values were calculated using a Student's t-test (paired, two-tailed). **E.** Quantification of percent NQO1+ immunostaining in KC mouse pancreas separated by expression in acinar and lesion regions of the tissue. n = 3 mice. Bars represent the mean with standard deviation. P-values were calculated using a Student's t-test (paired, two-tailed).

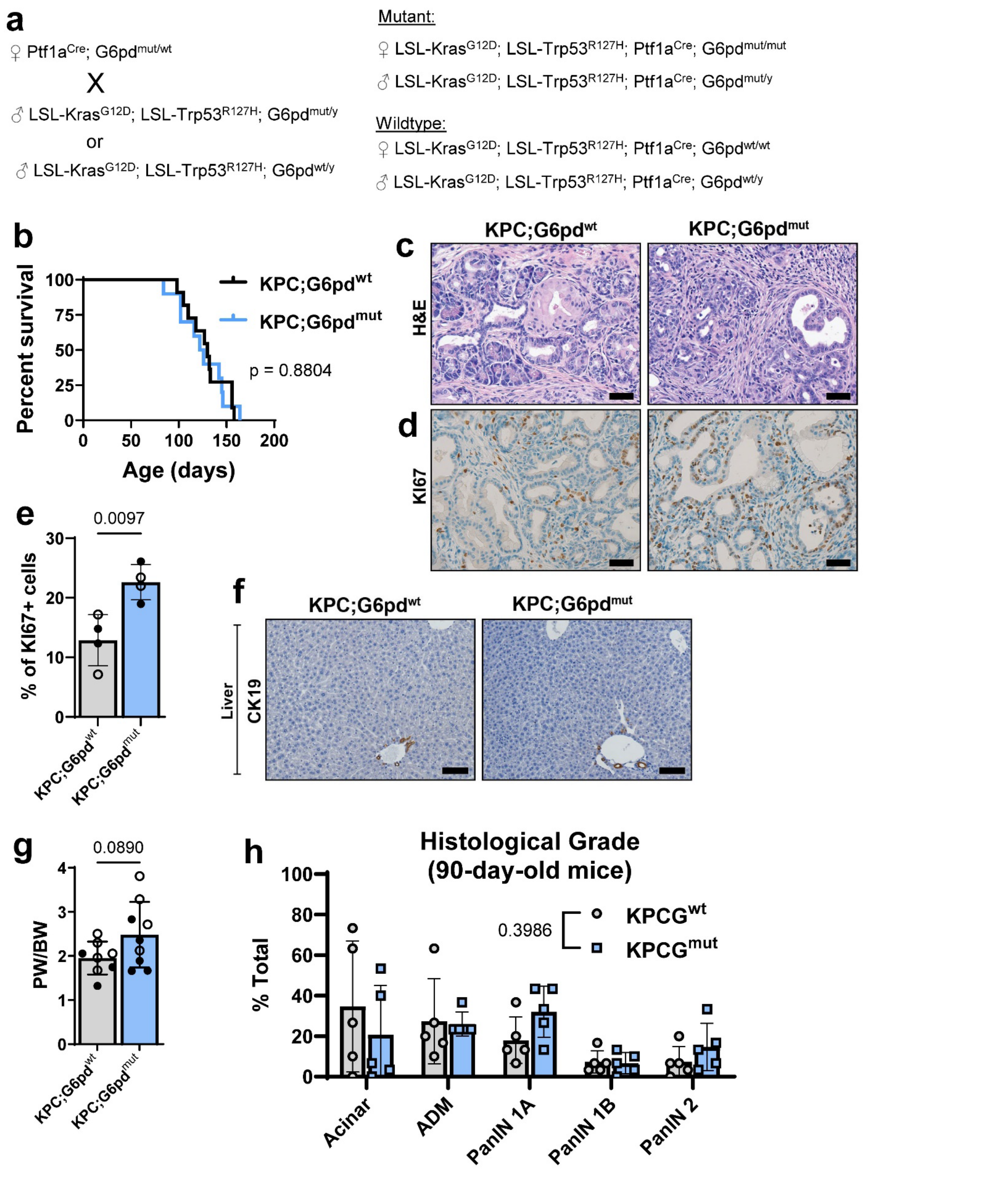

Supplementary Figure 4: Analysis of KPC;G6pd^{wt} and KPC;G6pd^{mut} mice
a. Example breeding schematic. Mice with mutant *G6pd*, that mimics human G6PD-deficiency, were bred into the KPC (*LSL-Kras^{G12D}; LSL-Trp53^{R127H}; Ptf1a^{Cre}*) model. *Cre* was always maintained in female breeders. Experiments used both male and female *G6pd* mutant mice, where females were homozygous for mutant *G6pd* (*G6pd$^{mut/mut}$*) and males were hemizygous for mutant *G6pd* (*G6pd$^{mut/y}$*), as *G6pd* is an X-linked gene.

G6pd wildtype mice used in experiments were age matched littermates obtained in the same colony of *G6pd* mutant mice when, possible. Female *G6pd* wildtype mice have two wildtype copies of *G6pd* ($G6pd^{wt/wt}$) and males have one wildtype copy (their only copy) of *G6pd* ($G6pd^{wt/y}$). In the schematics and labelling, "y" in male mice refers to the y chromosome, which does not contain a copy of *G6pd*. **b.** Kaplan–Meier survival curve of KPC;$G6pd^{wt}$ and KPC;$G6pd^{mut}$ mice. n = 11 KPC;$G6pd^{wt}$ mice; n = 10 KPC;$G6pd^{mut}$ mice. Survival analysis was performed using log-rank (Mantel–Cox) test. Bars are presented as mean with standard deviation. **c.** Hematoxylin & eosin (H&E) staining in 90-day-old KPC;$G6pd^{wt}$ and KPC;$G6pd^{mut}$ pancreas. Scale bar = 50μm. **d.** Immunostaining for Ki67 (proliferation) in 90-day-old KPC;$G6pd^{wt}$ and KPC;$G6pd^{mut}$ pancreas. Scale bar = 50μm. **e.** Percent of Ki67+ cells in pancreas tissue as quantified from male (closed circle) and female (open circle) mice in 90-day-old KPC;$G6pd^{wt}$ (grey bar) and KPC;$G6pd^{mut}$ (blue bar) mice. n = 4 for each genotype. Bars represent the mean with standard deviation. P-values were calculated using a Student's t-test (unpaired, two-tailed). **f.** Immunostaining for CK19 in 90-day-old KPC;$G6pd^{wt}$ and KPC;$G6pd^{mut}$ liver. Scale bar = 100μm. In healthy adult liver, CK19 is only expressed in cholangiocytes. **g.** Pancreas weight (PW) to body weight (BW) ratios in 90-day-old KPC;$G6pd^{wt}$ and KPC;$G6pd^{mut}$ mice. Bars represent the mean with standard deviation as quantified from male (closed circle) and female (open circle) mice. n = 8 KPC;$G6pd^{wt}$ mice; n = 9 KPC;$G6pd^{mut}$ mice. Ratios are not significantly different as calculated using a Student's t-test (unpaired, two-tailed). **h.** Pathological grading of 1-year-old pancreas tissues representing the % of total tissue area with acinar cells, ADM, and PanIN lesions. n = 5 mice per genotype. Bars represent the mean with standard deviation. P-values were calculated using a two-way ANOVA with Tukey post hoc test.

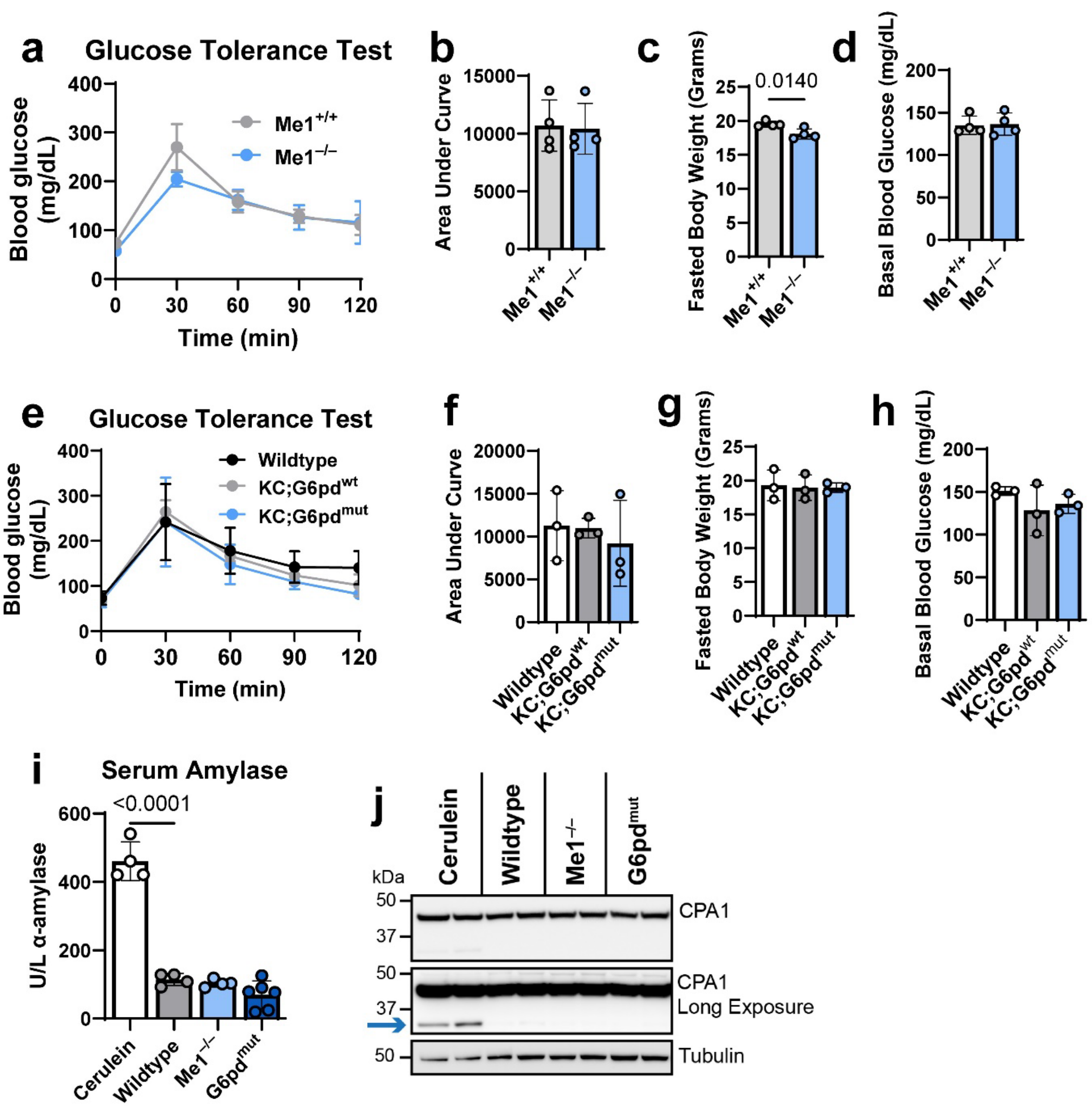

Supplementary Figure 5: Exocrine and endocrine function

a. Glucose tolerance tests performed in female $Me1^{+/+}$ and $Me1^{-/-}$ mice. Each datapoint represents the mean value at each timepoint with standard deviation. **b.** The area under the curve (AUC) from **a** was calculated for each individual mouse. **c.** Fasted body weight measurements in grams. **d.** Basal, resting glucose measurements in mice. **b-d**. n = 4 female mice per genotype aged 12-13 weeks old. Each bar represents the mean with standard deviation. P-values were calculated using a Student's t-test (unpaired, two-tailed). **e.** Glucose tolerance tests performed in female Wildtype, $KC;G6pd^{wt}$, and $KC;G6pd^{mut}$ mice. Each datapoint represents the mean value at each timepoint with standard deviation. **f.** The area under the curve (AUC) from **e** was calculated for each individual mouse. **g.** Fasted body weight measurements in grams. **h.** Basal, resting glucose measurements in mice. **f-h**. n = 3 female mice per genotype aged 14-15 weeks old. Each bar represents the mean with standard deviation. P-values were calculated using ordinary one-way ANOVA with Tukey's multiple comparisons test. **i.** Serum amylase activity measured in cerulein-treated wildtype mice (n = 4; 2 male, 2 female), Wildtype mice (n = 4 female), $Me1^{-/-}$ mice (n = 4 female), and $G6pd^{mut}$ mice (n = 6; 3 male, 3 female). Mice were 12-15 weeks old. Each bar represents the mean with standard deviation. P-values were calculated using ordinary one-way ANOVA with Dunnett's multiple comparisons test and compared to Wildtype. Where p<0.0001, the exact value = 7.4×10^{-9}. **j.** Western blots for protein levels of Carboxypeptidase A1 (CPA1) with Tubulin used as a loading control. Short and long exposures are shown for CPA1. CPA1 is primarily present in the pro-peptide form and the arrow points to the cleaved peptide, resulting from premature enzyme activation. Samples are pancreas lysates and biological duplicates, representative of each genotype. Western blot was repeated two independent times**.**

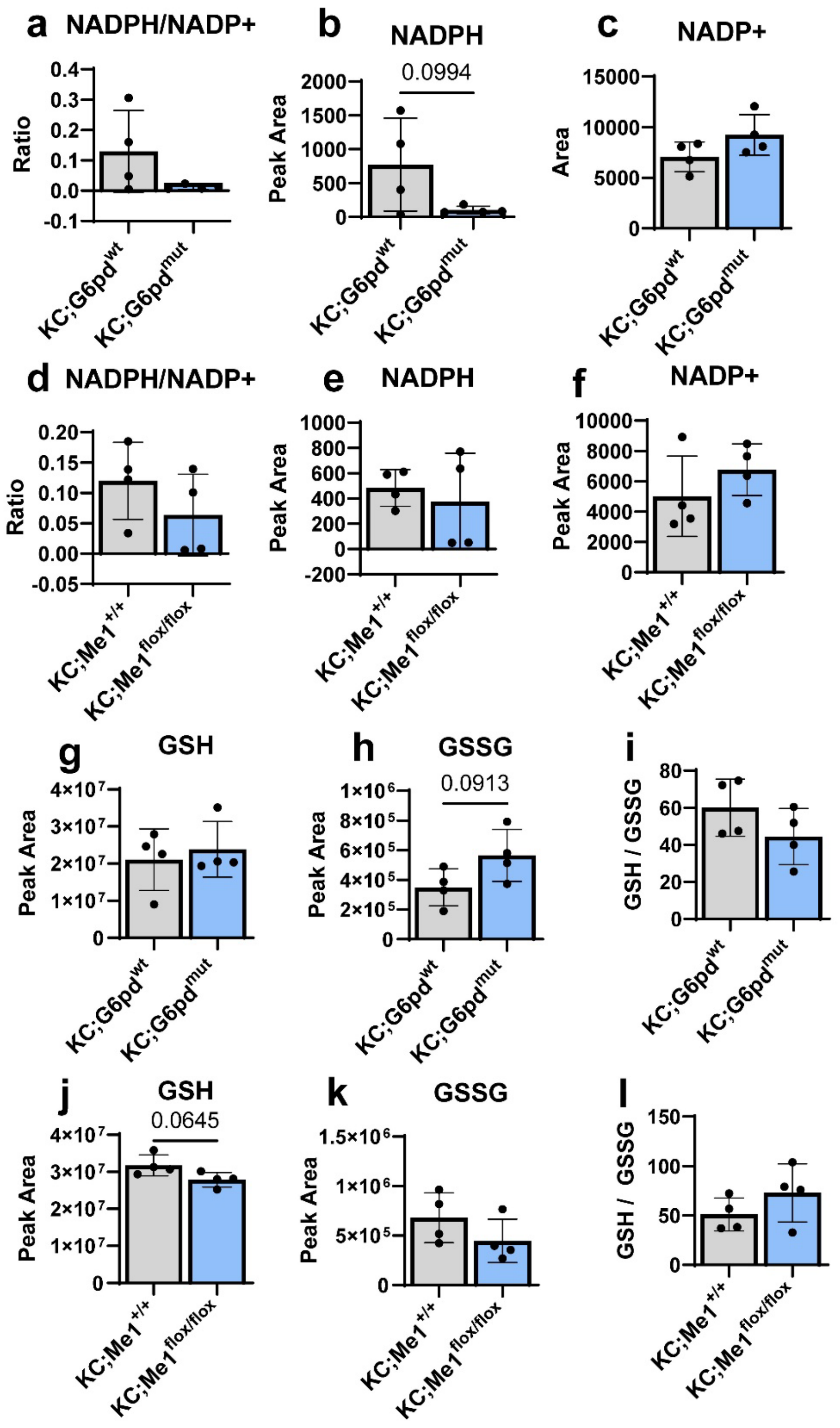

Supplementary Figure 6: NADPH, NADP+, GSH, and GSSG measurements in pancreas tissue
Mass spectrometry was used to measure NADPH, NADP+, GSH, and GSSG in whole pancreas tissue from 16-week-old mice of the indicated genotypes. n = 4 mice per genotype in each experiment. Each datapoint represents one mouse, each bar represents the mean of the biological replicates with standard deviation. P-values were calculated using a Student's t-test (unpaired, two-tailed). **a.** Ratio of NADPH/NADP+ peak areas from KC;G6pd^{wt} (grey bar) and KC;G6pd^{mut} (blue bar) mice. **b.** Peak area of NADPH and **c.** NADP+ from KC;G6pd^{wt} (grey bar) and KC;G6pd^{mut} (blue bar) mice. **d.** Ratio of NADPH/NADP+ peak areas from KC;Me1$^{+/+}$ (grey bar) and KC;Me1$^{flox/flox}$ (blue bar) mice. **e.** Peak area of NADPH and **f.** NADP+ from KC;Me1$^{+/+}$ (grey bar) and KC;Me1$^{flox/flox}$ (blue bar) mice. **g.** Metabolite peak areas of GSH and **h.** GSSG from KC;G6pd^{wt} (grey bar) and KC;G6pd^{mut} (blue bar) mice. **i.** Ratio of GSH to GSSG peak areas from **g** & **h**. **j.** Metabolite peak areas from GSH and **k.** GSSG from KC;Me1$^{+/+}$ (grey bar) and KC;Me1$^{flox/flox}$ (blue bar) mice. **l.** Ratio of GSH to GSSG peak areas from **j** & **k**.

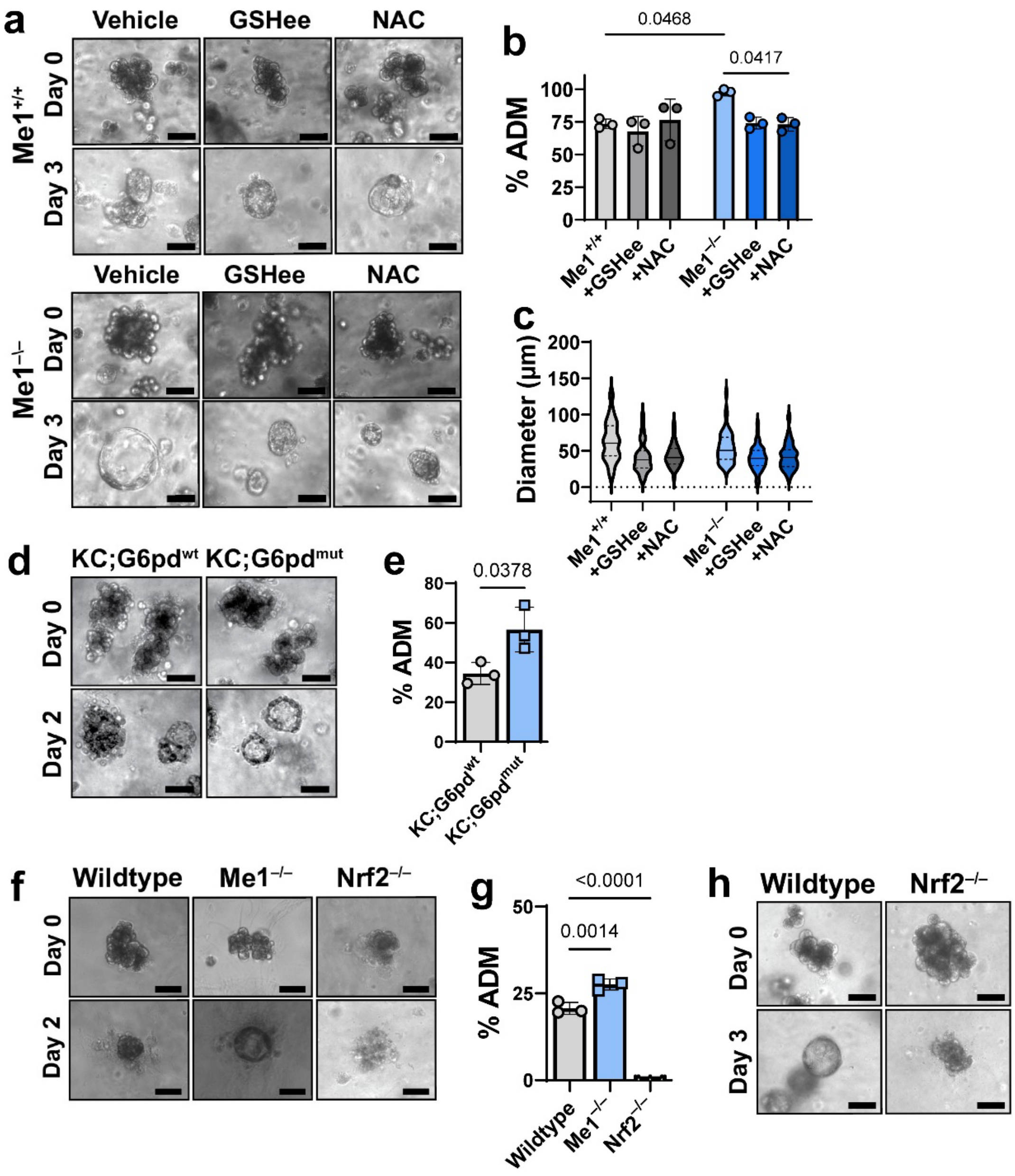

Supplementary Figure 7: Additional ADM culture experiments

a. Brightfield images of primary, *ex vivo* acinar cells from $Me1^{+/+}$ and $Me1^{-/-}$ mice grown in Matrigel for 3 days. Cells were treated with vehicle (water), glutathione ethyl ester (GSHee; 1mM), or N-acetylcysteine (NAC; 500µM). Scale bars = 25µm. **b.** Quantification of acinar cells undergoing ADM in primary cultures at Day 3. Each datapoint represents one mouse. n = 3 mice per genotype. Bars represent the mean with standard deviation. P-values were calculated using ordinary one-way ANOVA with Tukey's multiple comparisons test. **c.** Violin plots showing quantification of Diameter (µm) in ADM primary cultures at Day 3 from 1 biological replicate. Lines inside of the plots represent the quartiles and median. **d.** Brightfield images of primary, *ex vivo* acinar cells from KC;$G6pd^{wt}$ and KC;$G6pd^{mut}$ mice grown in collagen for 3 days. Scale bars = 25µm. **e.** Quantification of the % of acinar cells undergoing ADM in collagen cultures. n = 3 mice per genotype. Bars represent the mean with standard deviation. P-values were calculated using a Student's t-test (unpaired, two-tailed). **f.** Brightfield images of primary, *ex vivo* acinar cells from Wildtype, $Me1^{-/-}$, and $Nrf2^{-/-}$ mice grown in collagen for 2 days with TGFα treatment to induce ADM. Scale bars = 25µm. **g.** Quantification of the % of acinar cells undergoing ADM in collagen cultures from Wildtype (grey), $Me1^{-/-}$ (blue), and $Nrf2^{-/-}$ (green) mice. n = 3 mice per genotype. Bars represent the mean with standard deviation. P-values were calculated using ordinary one-way ANOVA with Dunnett's multiple comparisons test, comparing to the Wildtype mean. Where p<0.0001 the exact p-value is $3x10^{-6}$. **h.** Brightfield images of primary, *ex vivo* acinar cells from Wildtype and $Nrf2^{-/-}$ grown in Matrigel for 3 days with TGFα to induce ADM. Scale bars = 25µm.

Uncropped/unprocessed blots from Supplementary Figure 5j

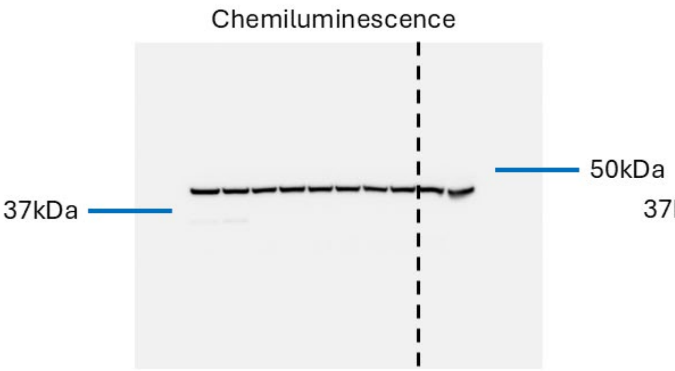

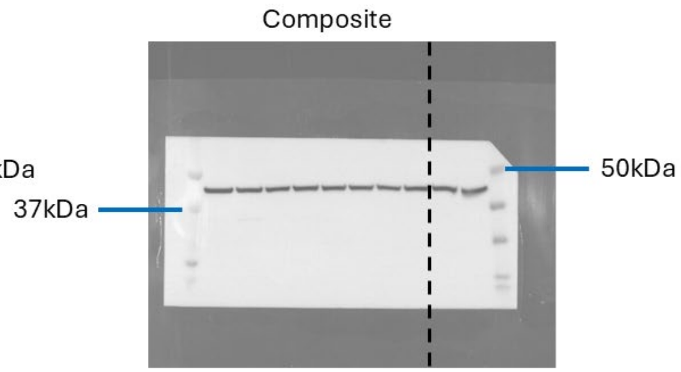

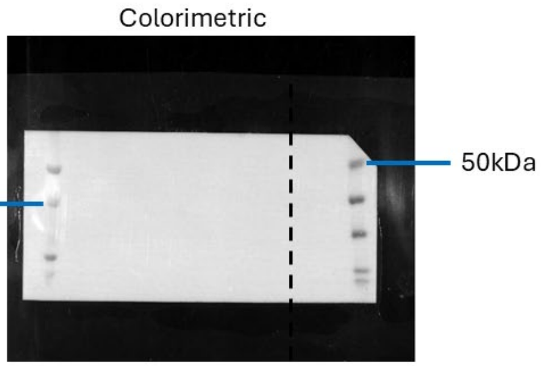

Supp. Fig 5j CPA1
(short exposure)

Left ladder = Kaleidoscope Prestained Protein Standards, BioRad, Cat No. 1610375
Right ladder = SeeBlue™ Plus2 Pre-stained Protein Standard, ThermoFisher, Cat No. LC5925

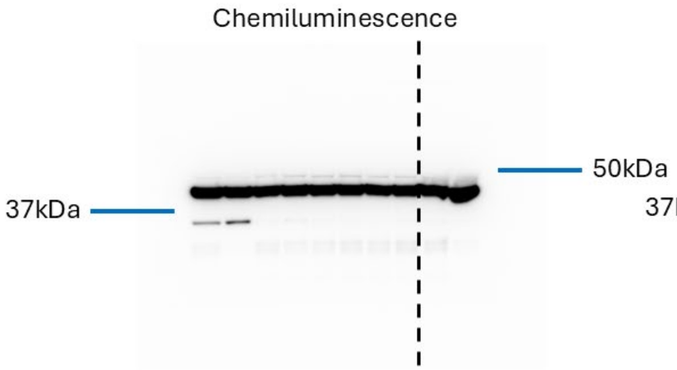

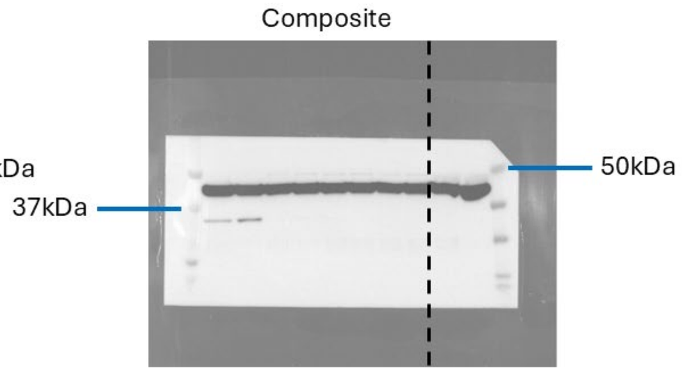

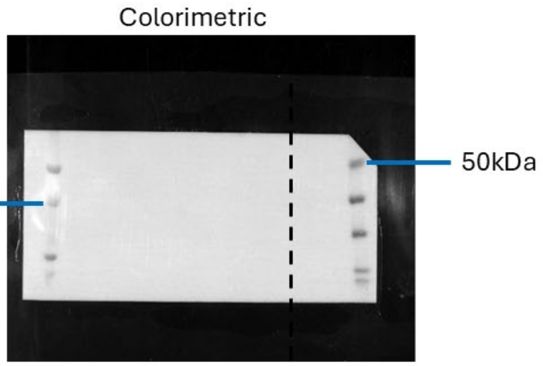

Supp. Fig 5j CPA1
(long exposure)

Left ladder = Kaleidoscope Prestained Protein Standards, BioRad, Cat No. 1610375
Right ladder = SeeBlue™ Plus2 Pre-stained Protein Standard, ThermoFisher, Cat No. LC5925

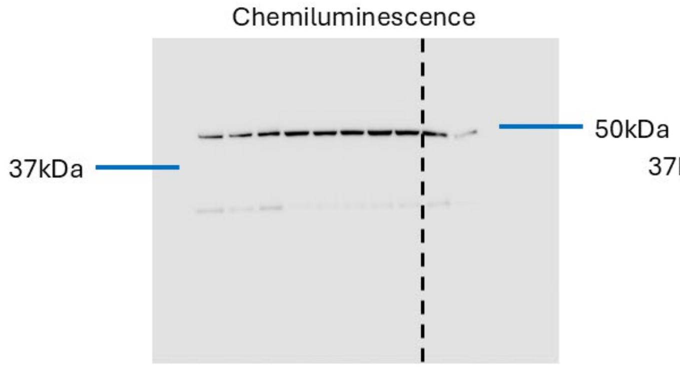

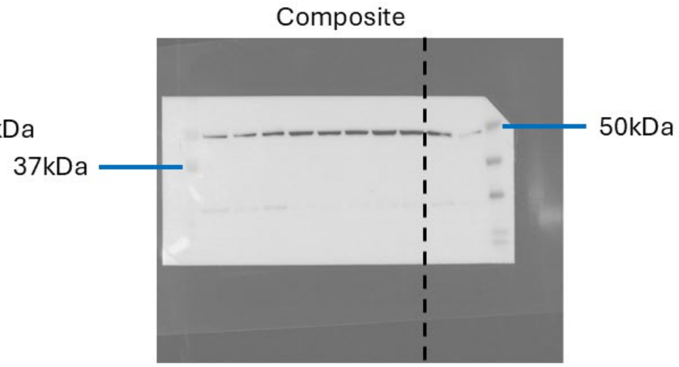

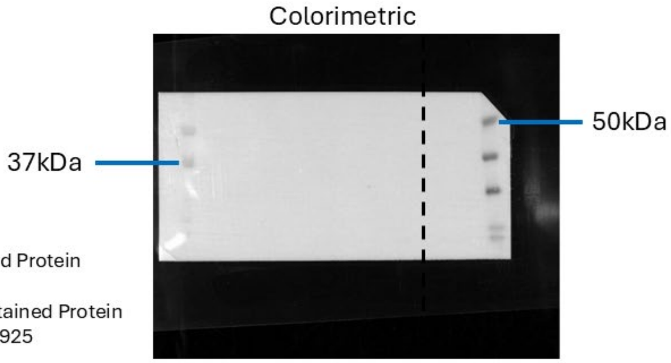

Supp. Fig 5j Tubulin

Left ladder = Kaleidoscope Prestained Protein Standards, BioRad, Cat No. 1610375
Right ladder = SeeBlue™ Plus2 Pre-stained Protein Standard, ThermoFisher, Cat No. LC5925
