# Supplementary material for: NADPH-producing enzymes restrict the formation of pancreatic precancerous lesions
Source: Nat Metab. 2026 Apr 1;8(4):824–39. doi: 10.1038/s42255-026-01496-x (PMC13121030; doi:10.1038/s42255-026-01496-x)
Supplement: Supplementary file 3 [file 42255_2026_1496_MOESM2_ESM.pdf]

nature portfolio

Corresponding author(s): Costas A Lyssiotis

Last updated by author(s): 2/27/2026

Reporting Summary

Nature Portfolio wishes to improve the reproducibility of the work that we publish. This form provides structure for consistency and transparency in reporting. For further information on Nature Portfolio policies, see our Editorial Policies and the Editorial Policy Checklist.

Statistics

For all statistical analyses, confirm that the following items are present in the figure legend, table legend, main text, or Methods section.

n/a	Confirmed	
☐	☒	The exact sample size (*n*) for each experimental group/condition, given as a discrete number and unit of measurement
☐	☒	A statement on whether measurements were taken from distinct samples or whether the same sample was measured repeatedly
☐	☒	The statistical test(s) used AND whether they are one- or two-sided<br>*Only common tests should be described solely by name; describe more complex techniques in the Methods section.*
☒	☐	A description of all covariates tested
☐	☒	A description of any assumptions or corrections, such as tests of normality and adjustment for multiple comparisons
☐	☒	A full description of the statistical parameters including central tendency (e.g. means) or other basic estimates (e.g. regression coefficient) AND variation (e.g. standard deviation) or associated estimates of uncertainty (e.g. confidence intervals)
☐	☒	For null hypothesis testing, the test statistic (e.g. *F*, *t*, *r*) with confidence intervals, effect sizes, degrees of freedom and *P* value noted<br>*Give P values as exact values whenever suitable.*
☒	☐	For Bayesian analysis, information on the choice of priors and Markov chain Monte Carlo settings
☒	☐	For hierarchical and complex designs, identification of the appropriate level for tests and full reporting of outcomes
☒	☐	Estimates of effect sizes (e.g. Cohen's *d*, Pearson's *r*), indicating how they were calculated

Our web collection on statistics for biologists contains articles on many of the points above.

Software and code

Policy information about availability of computer code

Data collection	RNA isolation and RNA-sequencing from acinar cell cultures: Acinar cells were grown free floating in non-tissue culture treated 6-well plates for RNA isolation. Primary acinar cell cultures were harvested by centrifugation for 2 minutes at 300 g. Half of the cell pellet was lysed in RLT buffer containing 1% β-mercaptoethanol (Sigma, M6250), flash frozen in liquid nitrogen, and stored at -80°C for brief storage. Samples were quickly thawed and passed through a Qiashredder column (Qiagen, 79654). RNA was purified using a RNeasy Plus Mini Kit (Qiagen, 74136) and analyzed on a Nanodrop 2000c (Thermo Scientific) spectrophotometer for quantification. RNA was further assessed with a Qubit Assay to determine concentration and quality assessment with an Agilent TapeStation through the University of Michigan Advanced Genomics Core. All samples for sequencing had a RIN greater than 7.9 and a DV200 greater than 90. Samples were submitted to GENEWIZ from Azenta Life Sciences for standard RNA-sequencing using Illumina NovaSeq X Plus with 20 million reads per sample and rRNA removal using PolyA selection for mRNA species. ERCC RNA Spike-In Mix (Cat: #4456740) from ThermoFisher Scientific, was added to normalized total RNA prior to library preparation following manufacturer's protocol. RNA sequencing libraries were prepared using the NEBNext Ultra II RNA Library Prep Kit for Illumina using manufacturer's instructions (NEB, Ipswich, MA, USA). Briefly, mRNAs were initially enriched with Oligod(T) beads. Enriched mRNAs were fragmented for 15 minutes at 94°C. cDNA fragments were end repaired and adenylated at 3'ends, and universal adapters were ligated to cDNA fragments, followed by index addition and library enrichment by PCR with limited cycles. The sequencing library was validated on the Agilent TapeStation (Agilent Technologies, Palo Alto, CA, USA) and quantified by Qubit 3.0 Fluorometer (Invitrogen, Carlsbad, CA) and quantitative PCR (KAPA Biosystems, Wilmington, MA, USA).
Data analysis	RNA-sequencing: RNA-sequencing fastq reads were transferred to the University of Michigan Great Lakes computational cluster for pseudoalignment to the mouse genome (Gencode version M35) using Salmon v1.9.043 to generate a counts per sample matrix. RNA-sequencing counts matrices were analyzed in edgeR to identify differentially expressed genes between experimental conditions. The expression data were filtered to remove genes with low counts using the edgeR filterByExpr() function with default settings. Normalization factors and dispersion parameters were then calculated prior to generating a log2-transformed counts per million (cpm) matrix for analysis.

Overall gene expression patterns per sample were first visualized with a multidimensional scaling plot. Differential gene expression between each timepoint (Day1/2/3) and Vehicle control was estimated using the quasi-likelihood negative binomial generalized log-linear approach in edgeR. Genes were considered differentially expressed between a treatment and control at a false discovery rate (FDR) adjusted p-value less than 0.05. Enriched differentially expressed genes per treatment were identified using Gene Set Enrichment Analysis via FGSEA44. To identify enriched transcription factor binding in differentially expressed gene lists, the top 500 overexpressed or under-expressed genes within a condition were uploaded to the Enrichr web tool25-27 to quantify enrichment for ENCODE and ChEA consensus transcription factor targets. We visualized gene expression patterns using pheatmap() in R or using GraphPad Prism. Analyses were conducted using R software version 4.2.2.

For manuscripts utilizing custom algorithms or software that are central to the research but not yet described in published literature, software must be made available to editors and reviewers. We strongly encourage code deposition in a community repository (e.g. GitHub). See the Nature Portfolio guidelines for submitting code & software for further information.

Data

Policy information about availability of data

All manuscripts must include a data availability statement. This statement should provide the following information, where applicable:

- Accession codes, unique identifiers, or web links for publicly available datasets
- A description of any restrictions on data availability
- For clinical datasets or third party data, please ensure that the statement adheres to our policy

RNA-sequencing from this study, referenced in Figure 1, is publicly available from NCBI's Gene Expression Omnibus (GEO) database (GSE313699). RNA-sequencing data referenced in Extended Data Figure 6 is publicly available from University of Michigan's Deep Blue Data Repository (10.7302/nd65-zg69). Human ADM RNA-sequencing data referenced in Extended Data Figure 10 was obtained from GEO (GSE179248). All metabolomics datasets are available in Supplementary Datasets 1-8.

Research involving human participants, their data, or biological material

Policy information about studies with human participants or human data. See also policy information about sex, gender (identity/presentation), and sexual orientation and race, ethnicity and racism.

Reporting on sex and gender	Sex and gender were not factored into analyses. Experiments were performed using donor pancreas from pancreas from 1 female and 3 males.
Reporting on race, ethnicity, or other socially relevant groupings	Race, ethnicity, or other socially relevant groupings were not factored into analyses. Experiments were performed using pancreas from 3 White patients and 1 African American patient.
Population characteristics	Donors were between the ages of 49-66
Recruitment	Acquisition of donor pancreas for research purposes through the Gift of Life Michigan (Ann Arbor, MI) has been described (PMID: 37021392). Donor pancreata that were ineligible for transplant or for which there were no eligible recipients were collected at the Gift of Life Michigan Donor Care Center and transported to the University of Michigan. Written consent from family members for utilization of tissues for research was obtained by the Gift of Life Michigan. Upon arrival, the pancreas was dissected into head, body, and tail regions by a pancreatobiliary surgeon. Tissue was used for primary, ex vivo acinar cultures. An adjacent region was fixed overnight in Z-fix (Anatech Ltd), rinsed with PBS and 70% ethanol, and paraffin-embedded for histology. Acquisition and use of donor pancreas for research purposes was approved by the Gift of Life Michigan research review group and the University of Michigan Institutional Review Board (HUM00025339). We have obtained informed consent and the studies complied with all relevant ethical regulations.
Ethics oversight	Acquisition and use of donor pancreas for research purposes was approved by the Gift of Life Michigan research review group and the University of Michigan Institutional Review Board (HUM00025339).

Note that full information on the approval of the study protocol must also be provided in the manuscript.

Field-specific reporting

Please select the one below that is the best fit for your research. If you are not sure, read the appropriate sections before making your selection.

☒ Life sciences ☐ Behavioural & social sciences ☐ Ecological, evolutionary & environmental sciences

For a reference copy of the document with all sections, see nature.com/documents/nr-reporting-summary-flat.pdf

Life sciences study design

All studies must disclose on these points even when the disclosure is negative.

Sample size	No statistical methods were used to pre-determine sample sizes but our sample sizes are similar to those reported in previous publications (PMID: 26947075, 31983610, 21734707). Sample sizes were selected based on standard experimental group sizes to achieve acceptable power taking into account the increased variability of animal models (3-4 replicates for ex vivo experiments, and 4-12 replicates for in vivo experiments).

Data exclusions	No animals were excluded from analyses. No datapoints were excluded from analyses, except in Extended Data Figure 10d where two outlier datapoints were removed. Outliers were identified by analyzing the raw dataset in GraphPad Prism using the ROUT (Robust Regression and Outlier Removal) method, Q=1%.
Replication	Experiments were repeated at least once and results were replicated, except for RNA-seq and metabolomics experiments which were performed once with independent biological replicates. Samples from animals were collected on several different days, from different litters, and handled by multiple investigators. Staining was performed by at least two independent investigators and replicated on separate days. RNA-seq and metabolomics experiments were run once using samples prepared from 3 biological replicates. Where applicable, sample size (n) of biological replicates and technical replicates are indicated in figure legends.
Randomization	Animals with the relevant genotypes were randomly placed into groups to collect tissue at each timepoint. Staining, analysis, and quantification wwere performed on samples from each group at random. However, for each timepoint and analysis, both male and female samples in approximately equal ratios were included.
Blinding	For staining from mouse tissues: an investigator imaged samples and genotypes were known. The images were then de-identified, randomized, and positive signal was quantified using QuPath software by a separate investigator in a blinded manner.<br>For tissue grading, slides were de-identified, imaged, and graded by a pathologist in a blinded manner.

Reporting for specific materials, systems and methods

We require information from authors about some types of materials, experimental systems and methods used in many studies. Here, indicate whether each material, system or method listed is relevant to your study. If you are not sure if a list item applies to your research, read the appropriate section before selecting a response.

Materials & experimental systems

n/a	Involved in the study	
☐	☒	Antibodies
☒	☐	Eukaryotic cell lines
☒	☐	Palaeontology and archaeology
☐	☒	Animals and other organisms
☒	☐	Clinical data
☒	☐	Dual use research of concern
☒	☐	Plants

Methods

n/a	Involved in the study	
☒	☐	ChIP-seq
☒	☐	Flow cytometry
☒	☐	MRI-based neuroimaging

Antibodies

Antibodies used	Goat anti-Carboxypeptidase A1 (CPA1), R & D Systems Cat# AF2765, Lot: WOD0124121, RRID:AB_2085841<br>Mouse anti-Malondialdehyde (MDA), Abcam Cat# ab243066, Lot: 1105129-13, RRID:AB_3662056<br>Rabbit anti-Hyperoxidized Peroxiredoxin-3 (PRDX3), Cayman Chemicals Cat# 39888, Batch: 0807961-1<br>Rabbit anti-NADPH:quinone oxidoreductase 1(NQO1), Atlas Antibodies Cat# HPA007308, Lot: C117281, RRID:AB_1079501<br>Rabbit anti-Glucose-6-phosphate dehydrogenase (G6PD), Abcam Cat# ab993, Lot: GR274589-51, RRID:AB_296714<br>Rabbit anti-Malic enzyme 1 (ME1), Proteintech Cat# 16619-1-AP, RRID:AB_2143821<br>Rabbit anti-Malic enzyme 1 (ME1), Santa Cruz Biotechnology Cat# sc-100569, Lot: L1821, RRID:AB_2143832<br>Rat anti-Cytokeratin 19 (CK19; TROMA-III), Developmental Studies Hybridoma Bank, TROMA-III-c, RRID:AB_2133570<br>Rabbit anti-KI67, Abcam, ab15580, Lot:1063776-1, RRID:AB_443209<br>Rabbit anti-Amylase, Sigma-Aldrich, A8273, Lot: 0000121533, RRID:AB_258380<br>Rabbit anti-4-Hydroxynonenal (4-HNE), Abcam, ab46545, Lot: GR3342496-2, RRID:AB_722490<br>Rabbit anti-Vinculin, Cell Signaling Technology Cat# 13901, Lot: 10, RRID:AB_2728768<br>Mouse anti-alpha-Tubulin, Cell Signaling Technology Cat# 3873, Lot: 19, RRID:AB_1904178<br>Horse anti-Mouse biotinylated secondary, Vector Laboratories Cat# BA-2000, Lot: ZH1018, RRID:AB_2313581<br>Horse anti-Rabbit biotinylated secondary, Vector Laboratories, Lot: ZH0421, BA-1100, RRID:AB_2336201<br>Goat anti-Rat biotinylated secondary, Vector Laboratories, Lot:ZJ0607, A-9400, RRID:AB_2336202<br>Rabbit anti-Goat HRP-conjugated secondary, R & D Systems Cat# HAF017, RRID:AB_562588<br>Horse anti-Mouse HRP-conjugated secondary, Cell Signaling Technology Cat# 7076, RRID:AB_330924<br>Goat anti-Rabbit HRP-conjugated secondary, Cell Signaling Technology Cat# 7074, RRID:AB_2099233
Validation	CPA1 - From the vendor, "Detects mouse Carboxypeptidase A1/CPA1 in direct ELISAs and Western blots. In direct ELISAs, approximately 20% cross-reactivity with recombinant human (rh) CPA1 is observed and approximately 10% cross-reactivity with rhCPA2, rhCPB1, and recombinant mouse CPA4 is observed"<br><br>MDA - From the vendor, "Specific for MDA conjugated proteins. Does not detect free MDA. Does not cross-react with Acrolein, Crotonaldehyde, Hexanoyl Lysine, 4-HHE, 4-HNE, or Methylglyoxal modified proteins. Anti-Malondialdehyde antibody [11E3] (ab243066) is a mouse monoclonal antibody and is validated for use in Western Blot (WB), Immunohistochemistry (IHC-P)." Relevant citation: PMID: 31983610<br><br>NQO1 - From the registry, "Independent validation by the NYU Lagone was performed for: IHC. This antibody was found to have the following characteristics: Functional in human:TRUE, NonFunctional in human:FALSE, Functional in animal:TRUE, NonFunctional in animal:FALSE." From the vendor, "Anti-NQO1 antibody produced in rabbit, a Prestige Antibody, is developed and validated by the

Human Protein Atlas (HPA) project . Each antibody is tested by immunohistochemistry against hundreds of normal and disease tissues. These images can be viewed on the Human Protein Atlas (HPA) site. The antibodies are also tested using immunofluorescence and western blotting." Relevant citations: PMID: 21734707, 40588523.

G6PD - From the vendor, "Suitable for IP, WB, ICC/IF and reacts with Mouse, Human samples. Cited in 65 publications. Immunogen corresponding to Synthetic Peptide within Human Glucose-6-phosphate 1-dehydrogenase aa 50-100."

ME1 (Proteintech) - From the vendor, "KD/KO Validated. Positive WB detected in HeLa cells, mouse liver tissue, mouse placenta tissue, MCF-7 cells, rat liver tissue. Positive IP detected in mouse liver tissue. Positive IHC detected in human liver tissue, human placenta tissue. Note: suggested antigen retrieval with TE buffer pH 9.0; Alternatively, antigen retrieval may be performed with citrate buffer pH 6.0. Positive IF/ICC detected in HeLa cells."

ME1 (Santa Cruz) - From the vendor, "ME1 Antibody (99.1) is a mouse monoclonal IgG1 κ, cited in 10 publications, provided at 100 µg/ml. Raised against recombinant ME1 of human origin recommended for detection of ME1 of mouse, rat and human origin by WB, IP, IF, IHC(P) and ELISA."

CK19 - From the registry, "Independent validation by the NYU Langone was performed for: IHC." Relevant citations: PMID: 6933460, 36727849

KI67 - KO validated by Abcam. From the vendor "Ab15580 is batch tested in ICC/IHC. A variability in IHC-Fr performance can occur with this antibody but we can guarantee consistency in IHC-P." From the registry, "Independent validation by the NYU Langone was performed for: IHC."

Amylase - From the registry, "Used for cytometry time of flight assay by the Human Islet Research Network community. PMIDs: 27732837." Relevant citations: PMID:32768422, 29153842

4-Hydroxynoneal - From the vendor " Suitable for WB and reacts with Chemical samples. Cited in 658 publications. Immunogen corresponding to Chemical / Small Molecule corresponding to 4-Hydroxynonenal." From the registry, "Independent validation by the NYU Langone was performed for: IHC."

Vinculin - From the vendor, "Vinculin (E1E9V) Rabbit Monoclonal Antibody recognizes endogenous levels of total vinculin protein. This antibody also reacts with metavinculin, a 145 kDa splice variant of vinculin. Species Reactivity: Human, Mouse, Rat, Monkey. Approved Applications: Flow Cytometry (Fixed/Permeabilized), IHC Leica Bond, Immunohistochemistry (Paraffin), Simple Western, Western Blotting. 685 citations."

alpha-Tubulin - From the vendor, "alpha-Tubulin (DM1A) Mouse Monoclonal Antibody detects endogenous levels of total α-tubulin protein. Species Reactivity: Human, Mouse, Rat, Monkey. Approved Applications: Flow Cytometry (Fixed/Permeabilized), Immunofluorescence (Immunocytochemistry), Immunohistochemistry (Paraffin), Simple Western, Western Blotting. 1136 citations."

Animals and other research organisms

Policy information about studies involving animals; ARRIVE guidelines recommended for reporting animal research, and Sex and Gender in Research

Laboratory animals	LSL-KrasG12D used to derive breeding pairs were obtained from Jackson Laboratory (Strain #008179)<br><br>LSL-KrasG12D; Ptf1aCreERTM mice were maintained on a C57BL/6J background and used for acinar culture experiments at 8-10 weeks of age.<br><br>LSL-KrasG12D; Ptf1aCre; G6pdmut or G6pdwt mice were maintained on a mixed background and were Nnt wild-type. They were assessed at 8 weeks, 16 weeks, 26 weeks, and 1 year of age. The mice used for acinar culture were 8-10 weeks of age. G6pdmut mice (previously described in doi:10.1007/BF00555491) were originally generated in the C3H strain. They have been crossed to other strains, including C57BL/6J. G6PD-deficient mice were maintained on a mixed background and compared to age-matched littermate controls. LSL-KrasG12D; Ptf1aCre; G6pdmut or G6pdwt mice were confirmed Nnt wild-type by genotyping.<br><br>LSL-KrasG12D; Ptf1aCre; Me1flox/flox mice and LSL-KrasG12D; Ptf1aCre; Me1+/+ mice were maintained on a C57BL/6J background and assessed at 8 weeks, 16 weeks, 26 weeks, and 1 year of age. These mice were all Nnt-null. Me1flox/flox mice previously described in doi:10.1371/journal.pone.0303577.<br><br>LSL-Tp53R172H; Ptf1aCre; G6pdmut or G6pdwt mice were maintained on a mixed background and were Nnt wild-type. These mice were assessed at 90 days old and endpoint (up to 164 days old).<br><br>Wildtype mice (C57BL/6J) aged 10 weeks old used as positive controls for serum amylase tests and anti-CPA1 western blots. Wildtype mice (C57BL/6J) aged 8-10 weeks old were used for acinar culture.<br><br>Germline, whole-body Me1-null mice (Me1–/–) were maintained on a C57BL/6J background and used for acinar culture at 8-10 weeks old. These mice were previously described in doi:10.1371/journal.pone.0303577.<br><br>Nrf2–/– mice (Jackson Laboratory Strain #017009) were maintained on a C57BL/6J background and used for acinar culture at 8-10 weeks old. These mice have been described in doi:10.2131/jts.28.455.
Wild animals	The study did not involve wild animals
Reporting on sex	The majority of experiments used both male and female mice, with two exceptions. Only male mice were used for RNA-sequencing

Reporting on sex	and only female mice were used for glucose tolerance tests, which were reported in the manuscript methods. Sex was determined by the Unit for Laboratory Animal Medicine (ULAM) mouse husbandry technicians based on anogenital distance upon weaning. Sex was carefully followed throughout the experiments and indicated in graphs as open/closed dots were appropriate. No significant sex differences were observed.
Field-collected samples	The study did not involve samples taken from the field
Ethics oversight	Animal experiments were conducted under the guidelines of the Office of Laboratory Animal Welfare and approved by the Institutional Animal Care and Use Committees (IACUC) at the University of Michigan under Lyssiotis protocol PRO00012367 (Approval 02/06/2025 - 02/06/2028), Shah protocol PRO00011805 (Approval 02/22/2024 - 02/22/2027), and formerly under Lyssiotis protocol PRO00010606 (Approval 02/09/2022 - 02/09/2025). Animal experiments complied with all relevant ethical regulations.

Note that full information on the approval of the study protocol must also be provided in the manuscript.

Plants

Seed stocks	N/A
Novel plant genotypes	N/A
Authentication	N/A
